# Bis-Rhodamines Bridged with a Diazoketone Linker: Synthesis, Structure, and Photolysis

**DOI:** 10.1021/acs.joc.1c01721

**Published:** 2021-12-17

**Authors:** Heydar Shojaei, Mariano L. Bossi, Vladimir N. Belov, Stefan W. Hell

**Affiliations:** † Department of NanoBiophotonics, Max Planck Institute for Biophysical Chemistry (MPIBPC), 37077 Göttingen, Germany; ‡ Department of Optical Nanoscopy, Max Planck Institute for Medical Research, 69120 Heidelberg, Germany

## Abstract

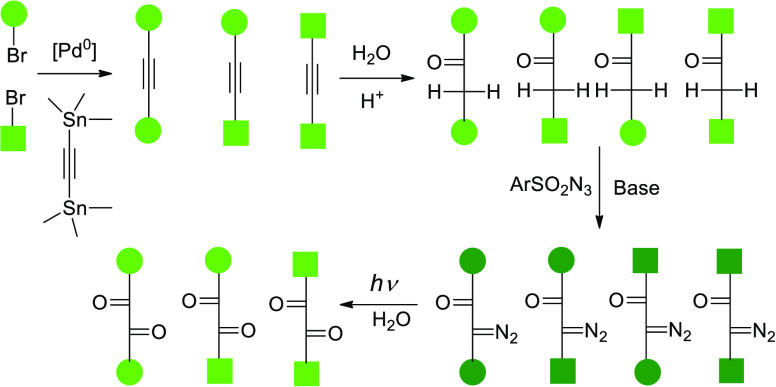

Two fluorophores bound with a short photoreactive bridge are fascinating structures and remained unexplored. To investigate the synthesis and photolysis of such dyes, we linked two rhodamine dyes via a diazoketone bridge (−COCN_2_−) attached to position 5′ or 6′ of the pendant phenyl rings. For that, the mixture of 5′- or 6′-bromo derivatives of the parent dye was prepared, transformed into 1,2-diarylacetylenes, hydrated to 1,2-diarylethanones, and converted to diazoketones Ar^1^COCN_2_Ar^2^. The high performance liquid chromatography (HPLC) separation gave four individual regioisomers of Ar^1^COCN_2_Ar^2^. Photolysis of the model compound—C_6_H_5_COCN_2_C_6_H_5_—in aqueous acetonitrile at pH 7.3 and under irradiation with 365 nm light provided diphenylacetic acid amide (Wolff rearrangement). However, under the same conditions, Ar^1^COCN_2_Ar^2^ gave mainly α-diketones Ar^1^COCOAr^2^. The migration ability of the very bulky dye residues was low, and the Wolff rearrangement did not occur. We observed only moderate fluorescence increase, which may be explained by the insufficient quenching ability of diazoketone bridge (−COCN_2_−) and its transformation into another (weaker) quencher, 1,2-diarylethane-1,2-dione.

## Introduction

**Scheme 1:**
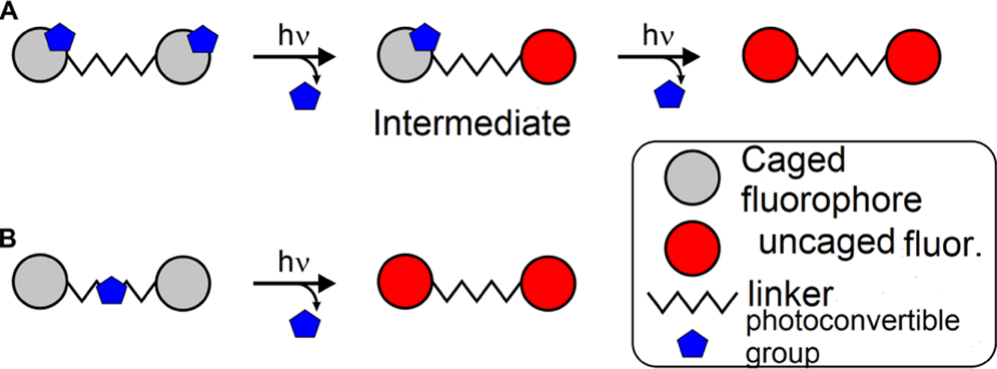
Combination of Two Caged Fluorophores Bound with a Linker (A) and an Alternative Based on a Single Photoreactive Caging Group Incorporated into a Linker (B)

The possibility to modify two fluorophores (and change the emission parameters of two dye residues) in the course of one photochemical reaction is intriguing and remained unexplored. If we consider two masked (caged) fluorophores bound with a linker (Scheme 1), the assembly may include two photoconvertible caging groups, one for each fluorophore (Scheme 1A). In this case, the photoactivation is stepwise, and the whole structure represents only a bare aggregate of two caged dyes. Alternatively, if a single photoreactive group efficiently suppresses the emission of the whole compound, and this group can be transformed into a nonquenching state, then both fluorophores may be activated in one step (Scheme 1B). This option is particularly challenging, as the quenching efficiencies of energy or electron transfer strongly depend on the distance. Therefore, we have chosen a potential fluorescence quencher and used it as a linker directly connecting two (identical) fluorophores.

**Figure 1:**
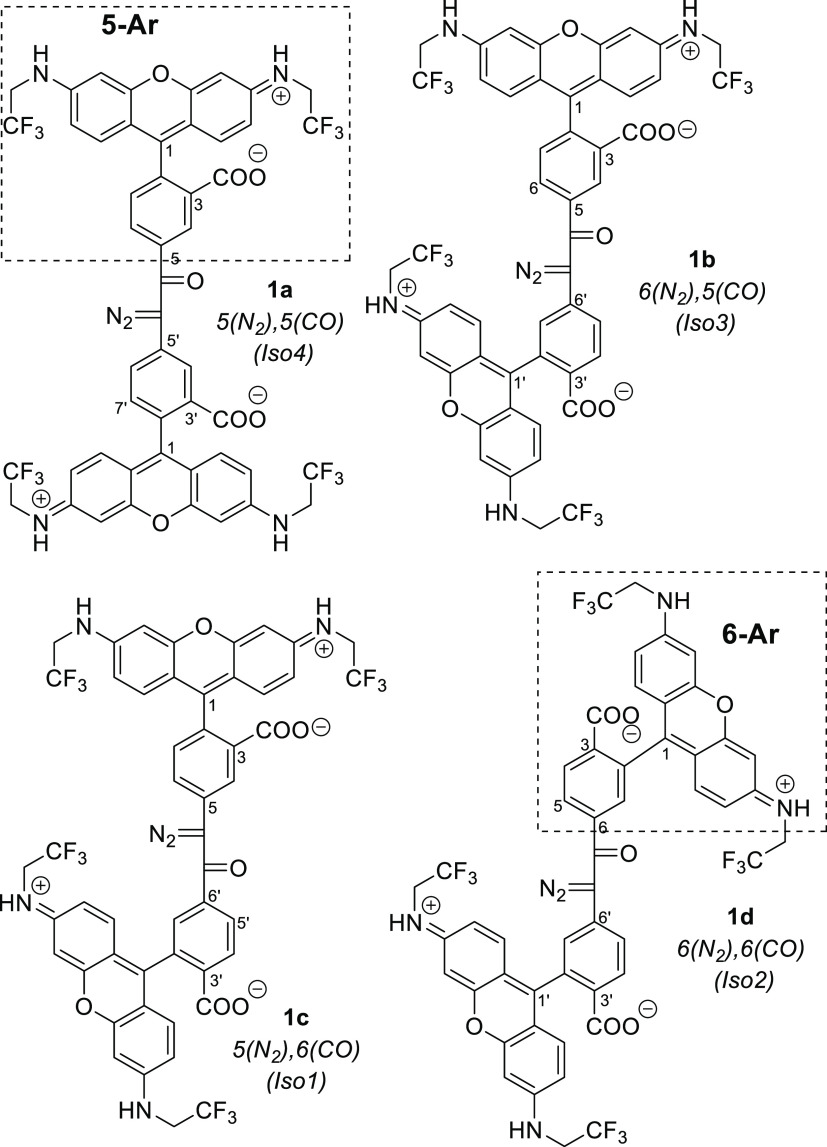
Diazoketone linkers −COCN_2_– connect two *N,N*′-bis(2,2,2-trifluorethyl)rhodamine residues via positions 5′ and 6′ of the pendant phenyl rings: four possible regioisomers **1a**–**d** and their designations Iso4, Iso3, Iso1, and Iso2 (for isomers 1–4, respectively) according to high performance liquid chromatography (HPLC) retention times.

The literature survey revealed that the fluorescein derivatives incorporating benzil fragments (Ar^1^COCOAr^2^) are essentially nonfluorescent (due to photoinduced electron transfer).^1−3^ Therefore, we applied photoconvertible 2-diazo-1,2-diarylethanones Ar^1^COCN_2_Ar^2^ closely related to Ar^1^COCOAr^2^, prepared bis-fluorophores bridged with a diazoketone linker, and studied their photolysis. Our motivation was to clarify whether the short diazoketone bridge (COCN_2_) incorporated between two dyes will suppress their emission, and whether a Wolff rearrangement will take place. As fluorophores, we have used *N,N*′-bis(2,2,2-trifluorethyl)-substituted rhodamines,^4^ which have absorption and emission spectra very similar to those of fluorescein. The structures of newly prepared compounds are given in Figure 1.

## Results and Discussion

### Synthesis

**Scheme 2:**
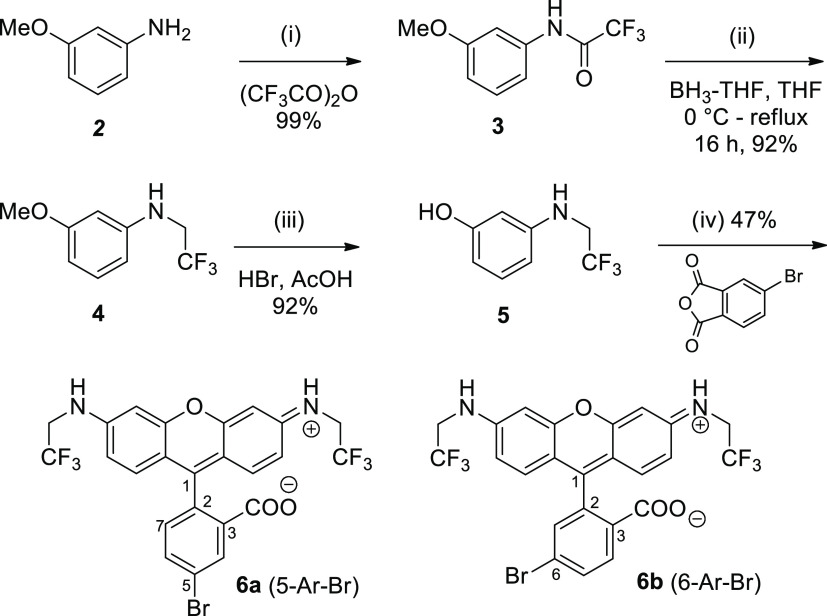
Synthesis of Regioisomeric Bromorhodamines **6a** and **6b** Containing *N,N*′-Bis(2,2,2-trifluoroethyl) Groups Conditions: (i) pyridine, CH_2_Cl_2_, rt, overnight; (iii) 48% aq. HBr, AcOH, reflux, 6 h; (iv) method A: 160 °C, 3 h; addition of **5** (2nd equiv), 85% aq. H_3_PO_4_, 160 °C, 3 h (47%); method B: 1,2-dichlorobenzene, 160 °C, 3 h, addition of **5** (2nd equiv), 160 °C, 3 h (31%).

**Scheme 3:**
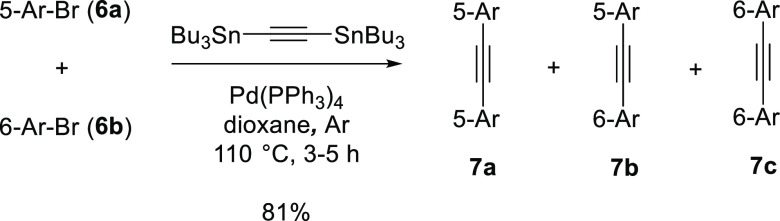
Bromides **6a**,**b** and 1,2-Bis(tributystannyl)acetylene in the Synthesis of Bis(rhodamine) Acetylenes **7a**–**c** as a Mixture of 5,5-, 5,6-, and 6,6-Regioisomers

The synthesis of bromorhodamines **6a,b** from aminophenol **5**^4^ is given in Scheme 2. In the condensation reaction leading to compounds **6a,b**, we compared two sets of conditions (see legend to Scheme 2). Higher yields (43–47%) were achieved when the first step was carried out without a solvent. Due to high temperature (160 °C) and the presence of water in the gas phase, the partial cleavage of the 2,2,2-trifluoroethyl amino group and the formation of the rhodol byproduct—a dye with the hydroxyl group instead of one CF_3_CH_2_NH residue—were observed. Under drastic condensation conditions, the undesired reaction was inevitable; it decreased the yields of the target compounds and complicated the isolation of pure dyes **6a,b**. For isolation of compounds **6a,b**, we applied chromatography on reversed-phase (C_18_ silica gel) because crystallization or chromatography on regular silica was not successful. The mixture of bromides **6a** and **6b** was stable by storing at −18 °C but slowly decomposed at room temperature. A high degree of purity (>95% HPLC area) was required for the success of the next coupling step (Scheme 3). Only by applying highly pure bromides **6a,b**, we were able to obtain acetylenes **7a–c** in synthetically useful amounts.

At the next step (Scheme 3), bromides **6a**,**b** were coupled with bis(tributylstannyl)acetylene and, as expected, provided a mixture of 3 compounds (**7a**–**c**). Isolation was performed by chromatography on reversed-phase and afforded a mixture of 5,5-, 5,6-, and 6,6-regioisomers in an overall yield of 81%.

The acetylene-bridged systems consisting of two fluorescent dyes linked directly through the triple bond belong to the family of through-bond energy transfer cassetten (TBET-C).^5−7^ The reaction conditions in Scheme 3 (for details, see the Experimental Section) may be applied for the synthesis of other TBET-Cs.

**Scheme 4:**
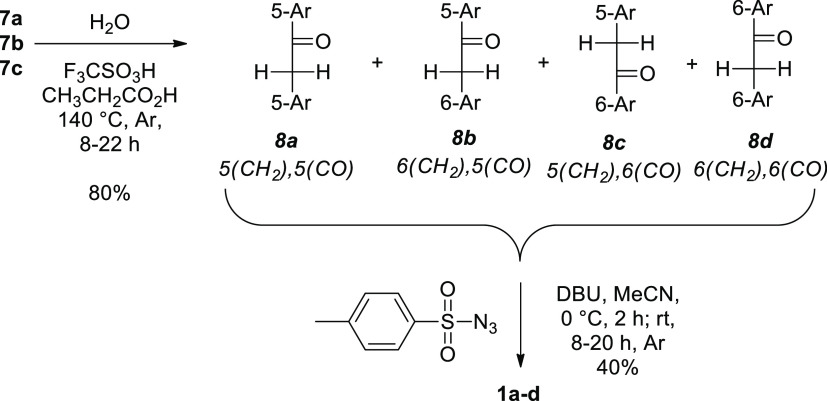
Hydration of **7a**, **7b**, and **7c** Mixture in the Presence of Triflic F_3_CSO_3_H (Reagent) and Propionic (Solvent) Acids Leads to the Mixture of Ketones **8a** [5(CH_2_),5(CO)], **8b** [6(CH_2_),5(CO)], **8c** [5(CH_2_),6(CO)], and **8d** [6(CH_2_),6(CO)] The Regitz diazotransfer with tosyl azide affords the target diazoketones **1a** [5(N_2_),5(CO)], **1b** [6(N_2_),5(CO)], **1c** [5(N_2_),6(CO)], and **1d** [6(N_2_),6(CO)]. For full structures of **1a–d**, see Figure 1.

**Scheme 5:**
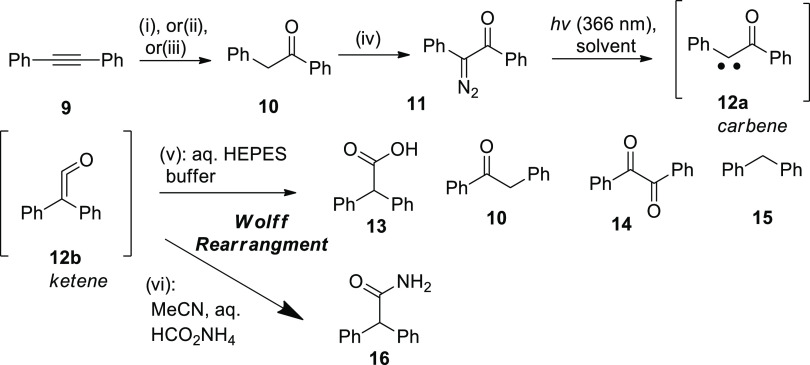
Synthesis and Photolysis of Azibenzil (**11**) Conditions: (i) aq. AcOH, Nafion NR50, 100 °C, 24 h, 70%; (ii) aq. Ga(F_3_CSO_3_)_3_, 100 °C, 24 h, 59%; (iii) aq. F_3_CCH_2_OH, F_3_CSO_3_H, MW, 90 °C, 1 h, 90%; (iv) TsN_3_, DBU, MeCN, 0 °C, 1 h, rt, 3–18 h, 53%; (v) MeCN, aq. HEPES buffer, pH 6.5, air; (vi) MeCN, aq. HCOONH_4_ buffer, pH 7.3–7.4, air.

The reported conditions of hydration reaction (Scheme 4) were first checked with diphenylacetylene (tolane (**9**) in Scheme 5) as a model. Transformation of tolane to deoxybenzoin **10** catalyzed by Nafion NR50,^8^ Ga(F_3_CSO_3_)_3_,^9^ or CF_3_SO_3_H in CF_3_CH_2_OH^10^ proceeded smoothly and with good yields. However, under all of these conditions, hydration of acetylenes **7a**–**c** was sluggish. With HSO_3_F (magic acid),^11^ Nafion NR50, Nafion 117, or *p*-toluenesulfonic acid, ketones did not form at all. Only by using great excess of water, trifluormethanesulfonic (TfOH, reagent), and propionic (solvent) acids at 140 °C, we managed to detect the formation of regioisomeric ketones (Scheme 4). The combinatorial fashion of the reaction sequence **6a**,**b**–**7a**–**c**–**8a**–**d** increased the number of regioisomers on each step. The hydration reaction proceeded through the corresponding vinyl esters formed from acetylenes and TfOH. Further optimization was required, to fully hydrolyze these esters to ketones **8a**–**d**. The HPLC analysis was difficult, due to numerous peaks with similar retention times. However, we managed to isolate a mixture of **8a**–**d** and then separate it to individual components **8a** [5(CH_2_),5(CO)], **8b** [6(CH_2_),5(CO)], **8c** [5(CH_2_),6(CO)], and **8d** [6(CH_2_),6(CO)] so that the overall yield was about 80%. For that, we used preparative HPLC on reversed phase with a gradient of acetonitrile in the basic aqueous buffer.

Bis(rhodamine)diazoketones **1a**–**d** (Figure 1) were prepared according to the modified and optimized procedure of M. Regitz using *p*-toluene sulfonyl azide and DBU as a base (Scheme 4).^12,13^ Diazoketones **1a**–**d** were sensitive to acids and decomposed under acidic conditions. They were isolated in milligram amounts and purified by means of preparative HPLC with acetonitrile and basic aqueous buffers (e.g., AcONH_4_ at pH 8.6). The overall preparative yield of all compounds **1a** [5(N_2_),5(CO)], **1b** [6(N_2_),5(CO)], **1c** [5(N_2_),6(CO)], and **1d** [6(N_2_),6(CO)] was about 40%. To avoid decomposition, the products were stored at −18 °C in the dark.^14^

### Structure Elucidation of Diazoketones **1a–d**

The regularities of ^1^H NMR spectra reported for 5- and 6-substituted (in the pendant phenyl ring) rhodamines^15^ allowed us to assign structures to compounds **1a**–**d** (Figure 1). Additionally, we used gCOSY and gHMBCAD spectra showing ^1^H–^1^H and multibond (optimized for three bonds) ^1^H-^13^C correlations, respectively. In the proton spectra, we observed six 1-proton multiplets corresponding to two 3-substituted benzene rings: one with CO and one with CN_2_ group. For isomer 1 (lowest retention time in HPLC), these signals were 8.09, 8.07, 7.92, 7.73, 7.56, and 7.20 ppm. In the gCOSY spectrum, we did not observe cross-peaks between 8.09 and 7.73 ppm, but all other cross-peaks required for two sets of three protons were present. We could conclude that the signal at 8.09 ppm belongs to the same set as the multiplets at 7.73 and 7.20 ppm, and the signals at 8.07, 7.92, and 7.56 ppm belong to another aromatic ring. In the gHMBCAD spectrum of this compound, we found that the ^13^C resonance in CO of the diazoketone has cross-peaks with multiplets at 7.56 and 7.92 ppm. Therefore, the signals at 8.07, 7.92, and 7.56 ppm belong to the ring linked with CO in COCN_2_, and the group of signals with δ = 8.09, 7.73, and 7.20 ppm—to the ring bound with CN_2_. In each set, the most high-field signal belongs to H-7(7′)—the proton nearby the fluorophore.^15^ This proton is shielded by the π-system of the fluorophore. The molecule is twisted, and H-7(7′) is out of the plane of the three fused six-membered rings. Thus, in the ring with CO, H-7′ is found at 7.56 ppm (weak splitting, 6-CO isomer), and for the ring with CN_2_, the signal at 7.20 ppm belongs to H-7 (strong splitting, 5-CN_2_ isomer). To confirm that there was no rearrangement (exchange of the oxo and diazogroups in the course of diazotransfer in Scheme 4), we isolated the precursor of compound **1c** (isomer 1). This ketone is named **8c** in Scheme 4 and Table 1. The structure of 1,2-diarylethanone-1 **8c** was established using the principles mentioned above, and **8c** was shown to be the “true” precursor of **1c**: [5(CH_2_),6(CO)]-**8c**.

**Table 1:** Chemical Shifts (δ, ppm) and Coupling Constants (*J*, Hz) of Aromatic Protons H-4–H-7 and H-4′–H-7′ in Compound **8c** (Scheme 4) and Diazoketones **1a–d** (Figure 1) in [D_4_]MeOH

compound	H-4 (*J*)	H-5 (*J*)	H-6 (*J*)	H-7 (*J*)	H-4′ (*J*)	H-5′ (*J*)	H-6′ (*J*)	H-7′ (*J*)
**8c** [5(CH_2_),6(CO)]	7.76 d (0.7)		7.52 dd (8.0, 1.5)	7.10 d (7.9)	8.28 dd (8.0, 1.4)	8.11 dd (8.0, 0.6)		7.90 d (0.7)
**1a** [5(N_2_),5(CO)] Isomer 4	8.24 d (1.4)		8.01 dd (7.9, 1.7)	7.39 d (7.9)	8.08 d (1.8)		7.93 dd (8.1, 1.9)	7.34 d (8.1)
**1b** [6(N_2_),5(CO)] Isomer 3	8.23 d (1.5)		7.93 dd (7.9, 1.6)	7.29 d (7.9)	8.14 d (8.3)	7.86 dd (8.2, 1.7)		7.44 d (1.3)
**1c** [5(N_2_),6(CO)] Isomer 1 ([D_3_]MeCN)	8.09 d (0.5)		7.73 dd (8.2, 1.8)	7.20 dd (8.2, 0.7)	8.07 dd (7.9, 0.8)	7.92 dd (7.9, 1.4)		7.56 dd (1.3, 0.8)
**1d** [6(N_2_),6(CO)] Isomer 2	8.11 dd (8.1, 2.6)	7.81 d (7.9)		7.35 s	8.06 dd (8.3, 2.5)	7.63 d (7.4)		7.22 s

### Photolysis of Azibenzil PhCOCN_2_Ph (11) and Bis-Rhodamines 1a–d Having Diazoketone Bridge

The main reactivity pattern of α-diazoketones and, in particular, azibenzil **11** (Scheme 5), which we used as a model compound, includes elimination of dinitrogen and formation of highly reactive carbenes.^16^ The reactions can be induced thermally, photochemical, or catalytically (acids, heavy metal oxides, and salts). The synthetically useful and well-studied reaction path includes the formation of carbene, its rearrangement into ketene, and the reaction with a nucleophile (e.g., water, alcohol, or amine); the overall transformation known as Wolff rearrangement (Scheme 5).^17^ The photochemically induced Wolff rearrangement discovered by Horner^14^ is advantageous because the photolysis is the most “ketene-rich” reaction path, while thermal or catalytic reactions lead mostly to the products of C–H insertion.^17,18^

Azibenzil (**11**)^19−21^ was prepared from tolane (**9**) as given in Scheme 5. The photolysis of azibenzil^22,23^ was performed under irradiation with 365 nm light in acetonitrile–water mixtures (80/20; v/v) in the presence of HEPES (pH 6.5) or HCOONH_4_ buffer (pH 7.3–7.4). The reaction mixtures were analyzed by means of HPLC with a UV–vis absorption (diode array) spectrometer and a mass spectroscopic detection (LC-MS). The expected product of the photolysis (in the absence of amines in the reaction solution)—diphenylacetic acid (**13**)^24^—was detected along with deoxybenzoin (**10**), benzil (**14**), and traces of diphenylmethane (**15**) (Scheme 5). These compounds were identified by comparison with commercial reference substances (retention times, UV, and mass spectra). In some experiments, we also detected products with higher masses: an oxazole formed upon [2 + 3] cycloaddition from acetonitrile and ketene **12b**,^25^ as well as small amounts of 3,3,6,6-tetraphenyl-1,2,4,5-tetroxane, the peroxide related to the photocyclization product of diphenylacetic acid.^26^

**Figure 2:**
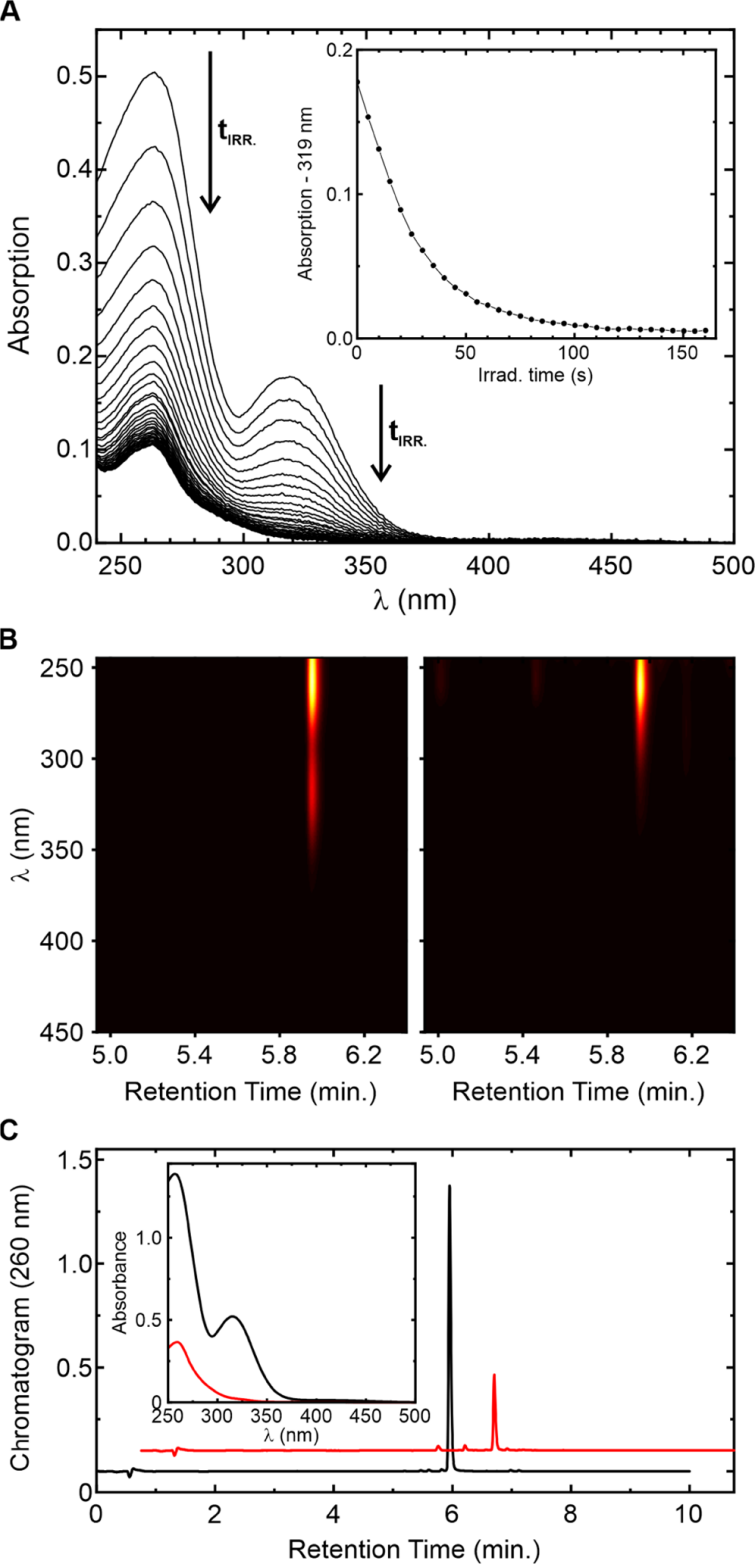
Irradiation of azibenzil (PhCOCN_2_Ph) dissolved in aqueous acetonitrile (80% acetonitrile, 20% water, v/v) with HCOONH_4_ buffer (pH 7.3) results in full conversion to a new substance (amide **16**, see Figure S2) with the same retention time but without absorption maximum at 319 nm. (A) Absorption changes upon irradiation; inset: transient at 319 nm. (B) Chromatograms (2D maps) of the sample before (left) and after (right) irradiation. (C) Chromatogram at 260 nm (a shift was introduced for clarity); inset: absorption spectra of the main peaks.

Photolysis of the solutions containing aqueous HEPES buffer provided complex mixtures with diphenylacetic acid (**13**) as one of the main products (Figure S1). Irradiation in the presence of aqueous HCOONH_4_ was found to be “cleaner” (Figure 2) and resulted in the formation of diphenylacetic acid amide (**16**; Scheme 5). Azibenzil **11** and amide **16** had the same retention times under conditions of HPLC separation. Unlike azibenzil (**11**) and benzil (**14**), amide **16** did not display the absorption maximum at about 320 nm. The composition of amide **16** was confirmed by HRMS data obtained for the reaction mixture (see Figure S2). The origin of amide **16** is obvious: it formed from ketene **12b** and ammonia, as the strongest nucleophile present in the equilibrium in aqueous ammonium formate (2 mM) at pH 7.3–7.4 (the initial concentration of azibenzil was 0.1 mM.) At physiological pH, ammonia may be considered as an analogue of biogenic amines,^27^ which have basicity similar to ammonia.

**Figure 3:**
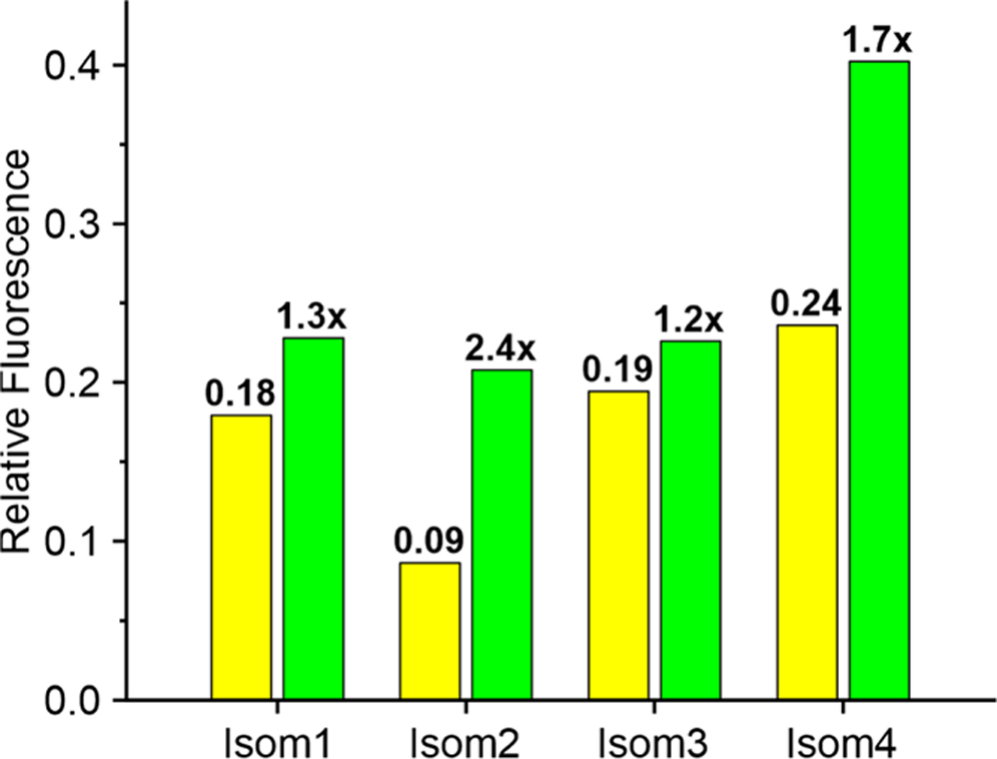
Relative fluorescence of isomers 1–4 (Figure 1) in MeCN (80% v/v) and 10 mM HCOONH_4_ buffer, pH = 7.4 (20% v/v). Yellow bars: starting materials. Green bars: after complete photolysis of the starting diazoketones. The numbers on top of the bars show the fluorescence quantum yields for the starting compounds and their increase upon photolysis to mixtures containing α-diketones as the main products (see Figure S3).

**Scheme 6:**
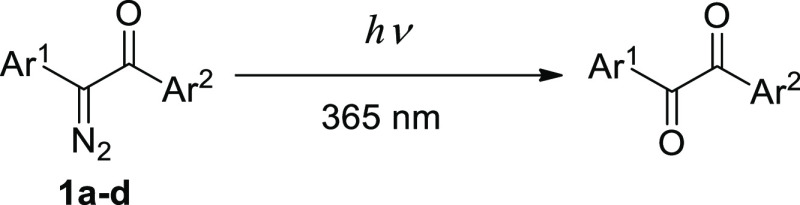
Photolysis of the Bis(rhodamine) Diazoketones **1a**, **1b**, **1c**, and **1d** The main product is shown. Solvent: acetonitrile/water 80/20 (v/v), aqueous HCOONH_4_ buffer (pH 7.3–7.4).

Having in mind the encouraging results obtained with model diazoketone **11**, we performed the photolysis of diazoketones **1a–d** (12 μM) in aqueous acetonitrile (acetonitrile/water = 80/20; v/v) in the presence of ammonium formate buffer (pH 7.3-7.4) (Scheme 6). Surprisingly, in this solvent, diketones Ar^1^COCOAr^2^ were the main products formed upon full conversion of the starting diazoketones **1a**–**d**. The LC-MS data (Figure S3a) indicated that the molecular masses of the photolysis products were always 12 Da lower than the molecular masses of diazoketones **1a**–**d**. A mass difference of −12 Da corresponds to the elimination of nitrogen (−28) and the addition of one oxygen atom (+16). For diazoketones **1a**–**d**, the Wolff rearrangement is disfavored, probably because the migration ability of the bulky and heavy dye residue is reduced. The fluorescence signals (and their quantum yields) of diazoketones **1a**–**d** and the mixture of products obtained from their photolysis are given in Figure 3. The emission efficiencies of compounds **1a**–**d** vary in the range of 0.09–0.24. Their emission is reduced, compared with the parent rhodamines, which are highly fluorescent,^4^ but not completely quenched by the presence of the diazoketone bridge. The diazoketone residue turned out to be an inefficient quencher, at least for these rhodamine dyes. The comparison of the absorption spectra recorded before and after photolysis is given in Figure S4b. Compounds **1a–d** have 3–4 times higher absorption at 365 nm (irradiation wavelength) than the parent fluorophore—*N,N*′-bis(2,2,2-trifluoroethyl)rhodamine.^4^ The presence of the azibenzil chromophore (Figure 2, abs. max. 325 nm) is masked by the relatively strong absorption of the parent dye with a maximum at 290 nm (Figure S4b). The photolysis of compounds **1a**–**d** was accompanied by an increase in emission by 20–240% (Figures 3, S4a, and S5). On the other hand, the relative absorption intensity at 300–310 nm decreased, after the photolysis was complete. The absorption spectra of the products and the parent rhodamine dye are much more similar to each other than the absorption spectra of diazoketones **1a–c**, which differ from each other considerably (Figure S4b). As expected, isomers 1 and 3 (compounds **1b** and **1c** in Figure 1) gave the same diketone 5-ArCOCOAr-6. The products’ retention times (Figure S3a) and emission gains were very similar: 30 and 20%, respectively (Figure 3). For all diazoketones, the photoactivation ratios (1.2–2.4) are moderate, if compared with dyes having two 2-nitrobenzyloxycarbonyl residues attached to the nitrogen atoms in one fluorophore,^28^ photoactivatable rhodamine spiroamides,^29^ or rhodamines incorporating the spiro-diazoketone fragment.^30^

This result may be explained if we assume that the quenching ability of diazoketone COCN_2_ is higher than that of α-diketone COCO, but the former does not completely inhibit the emission, while the latter does not allow to unfold the full fluorescence signal pertinent to two fluorophores. In addition, the quenching ability of the COCN_2_ residue toward “left” (Ar^1^) and “right” (Ar^2^) aryl groups in Ar^1^COCN_2_Ar^2^ is expected to be different, and may also depend on the substitution pattern of the aromatic ring (i.e., 5′ or 6′). The Wolff rearrangement is unfavored because the migration ability of the very bulky dye residue is low.

## Conclusions

We prepared and studied the photolysis of assemblies consisting of the two identical fluorophores directly bound with a short, compact, and photoconvertible diazoketone bridge (−COCN_2_−). Structurally, this approach to compounds in which two fluorophores can be activated with one photon is simpler than the design of sophisticated assemblies containing one photoconvertible unit (FRET acceptor) bound with two fluorescent dyes (FRET donors).^31^ In the course of photolysis, we observed only a moderate fluorescence increase. However, this method may be easily extended to compounds with other, more efficient quenchers linking two fluorescent dyes and undergoing photoconversion into another, essentially nonquenching state.

## Experimental Section

### General Remarks

The reactions were performed with magnetic stirring under argon. Oil baths were used for heating the reaction mixtures, and the bath temperatures are given as reaction temperatures. Evaporations in vacuum were performed in a rotary evaporator with bath temperature not exceeding 40 °C. NMR spectra were recorded at 25 °C on an Agilent 400-MR (400 MHz ^1^H and 100.5 MHz ^13^C). All spectra are referenced to tetramethylsilane (δ = 0 ppm) using the signals of the residual protons of CHCl_3_ (7.26 ppm) in CDCl_3_, CHD_2_OD (3.31 ppm) in CD_3_OD (49.15 ppm for ^13^C), CHD_2_CN (1.94 ppm) in CD_3_CN (1.39 and 189.69 ppm for ^13^C), or [D_5_]DMSO (2.50 ppm for ^1^H); 39.5 ppm for ^13^C in [D_6_]DMSO. Multiplicities of signals are described as follows: s = singlet, br. = broad signal, d = doublet, t = triplet, q = quartet, m = multiplet, dd = doublet of doublets. Coupling constants (*J*) are given in hertz. Structural assignments for asymmetric acetylenes **7a–c**, ketones **8a–d**, and diazoketones **1a–d** were made with additional information from gCOSY, gHSQC, and gHMBC experiments. Mass spectra with electrospray ionization (ESI-MS) were recorded on a Varian 500-MS spectrometer (Agilent). ESI-HRMS were measured on a MICROTOF spectrometer (Bruker) equipped with an Apollo ion source and a direct injector with an LC-autosampler Agilent RR 1200. Analytical RP-HPLC was carried out with Knauer Azura or Thermo Fisher Scientific (Ultimate 3000) systems equipped with diode array detectors. Solvent A: H_2_O + 0.1% v/v TFA; solvent B: MeCN + 0.1% v/v TFA. For the Knauer HPLC system: analytical column US10C18HQ-250/P46 (Interchim, 250 mm × 4.6 mm, 10 μm, flow rate 1.2 mL/min). LC-MS analyses were performed with Thermo Fisher Scientific ISQ EM mass spectrometer (coupled to Ultimate 3000 system) using a gradient of acetonitrile (20–100%, if not stated otherwise) in water (with the addition of 0.1% v/v HCOOH to both solvents). Preparative HPLC separations (reversed phase) were accomplished on an Interchim puriFlash 4250 device with a 250 × 21.2 mm column PF5C18AQ (10 μ, flow rate 20 mL/min) or a column from Knauer GmbH (Berlin, Germany), 250 × 30 mm, 5 μ, flow rate 45 mL/min. The mixtures of acetonitrile with 50 mM aqueous solutions of AcONH_4_ (pH ∼6.9) or 50 mM HCOONH_4_ (pH ∼6.5) were used as neutral buffers for the isolation of diazoketones **1a–d**. Individual regioisomers **7a–c** and **8a–d** were isolated by preparative HPLC. Column: Phenomenex Kinetex, 5 μm C18, 250 × 21 mm. Solvent A: H_2_O + 0.05% v/v TFA; solvent B: MeCN. Gradient A/B: 70/30–0/90 (0–20 min), flow rate 18 mL/min, 22°C; detection at 500 nm. Analytical TLC (normal phase) was performed on MERCK ready-to-use plates with silica gel 60 (F_254_). The spots were detected under UV light (254 and 365 nm). TLC on reversed phase: silica gel 60 RP-18 F_254S_, 50 × 75 mm plates purchased from MERCK (Darmstadt, Germany). Automated flash column chromatography was carried out using cartridges with regular silica gel on a Biotage Isolera One device. *m*-Anisidine (**2**) was purchased from Sigma-Aldrich; compounds **3**–**5** were prepared as described in ref (4).

### Photochemistry

Irradiation experiments were performed in a home-build setup,^32^ using a 365 nm LED as irradiation source (M365-L2, Thorlabs), a deuterium/xenon lamp (DH-2000-BAL, Ocean Optics) as an illumination source (for recording absorption spectra), and a diode array spectrometer (FLAME-S-UV–VIS–ES, Ocean Optics). The intensity of the irradiation light was calibrated with a chemical actinometer (Azobenzene in MeOH). The samples were kept at 20 °C and continuously stirred with a Peltier-based temperature controller (Luma 40, Quantum Northwest, Inc.). The absorption of the samples was recorded at a right angle with respect to the irradiation source, at fixed irradiation intervals until complete conversion to the final product. At fixed intervals, a small sample was extracted to perform LC-MS experiments (Shimadzu LCMS-2020).

#### 5′-Bromo-*N,N*′-bis(2,2,2-trifluoroethyl)rhodamine (6a) and 6′-Bromo-*N,N*′*-*bis(2,2,2-trifluoroethyl)rhodamine (**6b**)

In a pear-shaped flask, powdered phthalic anhydride (500 mg, 2.20 mmol, 1.0 equiv) and powdered aminophenol **5**^4^ (317 mg, 1.66 mmol, 0.75 equiv) were well mixed and heated under argon at 170 °C for 3 h. The course of the reaction was monitored via HPLC and TLC. After no more changes in HPLC and TLC were detected, another portion of aniline **5** (253 mg, 1.32 mmol, 0.6 equiv) and 85% aq. H_3_PO_4_ (2.0 mL) were added and the heating was continued at 160 °C for 3 h. After cooling to rt, the red mixture was taken up in ethyl acetate, washed with aqueous NaHCO_3_ (2 × 50 mL), sat. NaCl solution (50 mL), dried over MgSO_4_, and evaporated to get 1.37 g of a glassy red solid. It was dissolved in ethyl acetate, applied onto Celite, dried, and subjected to flash chromatography (regular SiO_2_, RediSep Rf cartridge 120 g, 25 μM; gradient: 10 to 85 v/v% AcOEt in hexane). The red fractions containing the product were pooled and concentrated to give 602 mg (48%) of the title compound as a bright red solid. TLC (SiO_2_) hexane/AcOEt 1:2, *R*_f_ (mixture **6a**, **6b**) = 0.33. TLC (reversed phase): MeCN:H_2_O, 7:3; *R*_f_ (mixture **6a**, **6b**) = 0.19. Analytical HPLC (Knauer, A/B = 70/30–0/100 in 20 min, λ = 530 nm): *t*_R_ = 11.3 min and 11.6 min (1:1; sum of peak areas 100%). As a byproduct, we isolated 172 mg (14%) of compounds with one oxygen atom instead of one CF_3_CH_2_NH group (dark orange solid). For additional purification, the product was dissolved in a minimal amount of aqueous MeCN and subjected to preparative HPLC (Interchim; gradient MeCN/H_2_O: 20/80–70/30 with 0.1 v/v% of HCOOH in both components); detection interval 200–600 nm, column Knauer (see the General Remarks section). The pure fractions were pooled and evaporated; the residue dissolved in 1,4-dioxane and filtered through a 0.2 μM PTFE membrane filter. The dioxane solution was frozen and lyophilized to yield 550 mg (44%) of compounds **6a,b** as red solid. Mixture of 5′- and 6′-COOH isomers in ca. 1:1 ratio. ^1^H NMR (CD_3_CN, 400 MHz) δ 8.11 (d, 0.5H, *J* = 1.8, H-4′ in 5′-isomer), 7.89–7.75 (m, 1H, H-6′ in 5′-isomer and H-4′ in 6′-isomer), 7.41 (d, 0.5H, *J* = 1.5, H-7′ in 6′-isomer), 7.11 (d, 0.5H, *J* = 8.2, H-7′ in 5′-isomer), 6.64–6.55 (m, 4H), 6.46 (m, 2H), 5.27 (t, 2H, *J* = 7.0, NH), 3.88 (m, 4H, CH_2_CF_3_). ^13^C{^1^H} NMR (CD_3_CN, 101 MHz) δ 169.7, 169.1 (COOH), 156.1, 154.0, 153.0, 151.1, 133.8 (all C_q_), 139.4 (CH), 134.6 (CH), 130.9 (C_q_), 130.6 (C_q_), 130.3 (2 × CH), 128.8 (CH), 128.5 (CH), 128.3 (C_q_), 127.6 (CH), 127.5 (C_q_), 127.2 (CH), 127.0 (q, *J* = 280, CF_3_), 124.5 (C_q_), 111.6 (2 × CH), 109.3 (C_q_), 99.9 (2 × CH), 44.3 (2 × q, *J* = 44, CH_2_CF_3_). ^19^F NMR ([D_6_]DMSO, 376 MHz) δ −70.5 (t, *J* = 9.6). HRMS (ESI-TOF) *m*/*z*: [M + H]^+^ calcd for C_24_H_16_BrF_6_N_2_O_3_ 573.0243; found 573.0224. *m*/*z*: [M + Na]^+^ calcd for C_24_H_15_BrF_6_N_2_O_3_Na 595.0062; found 595.0042.

#### Compounds **7a–c**

A 1:1 mixture of 5′- and 6′-bromorhodamines **6a**,**b** (2.82 g, 4.9 mmol, 2.0 equiv) and Pd(PPh_3_)_4_ (284 mg, 0.25 mmol, 0.1 equiv) was transferred into a screw-cap 100 mL pressure tube and purged with argon for 5 min. Degassed dioxane (45 mL) and bis(tributylstannyl)acetylene (1.29 mL, 1.48 g, 2.46 mmol, 1.0 equiv) were added, and the reaction mixture was purged with argon for 10 min. The reaction vial was closed, and the red reaction solution was heated to 110 °C with stirring for 5 h. The course of the reaction was monitored by TLC, LCMS, or HPLC. After the reaction was complete, the reaction mixture was cooled to rt, water (50 mL) was added, and the mixture was extracted with ethyl acetate (3 × 50 mL). The combined organic solutions were washed with brine and dried over MgSO_4_. The solvents were removed under reduced pressure. The residue (3.5 g) was dissolved in ethyl acetate, applied onto Celite, and subjected to flash chromatography in two portions. Cartridge with 40 g of regular SiO_2_ (Puriflash, 15 μm); eluent CH_2_Cl_2_: MeOH = 90:10 to 35:65 to 50:50 (%, v/v). The fractions containing compounds **7a**–**c** were pooled and concentrated under reduced pressure, excluding light and atmospheric oxygen. The residue was dissolved in dioxane, filtered through a 0.2 μM TFFP filter, frozen, and lyophilized. Yield 2.01 g (81%) of the mixture **7a**, **7b** and **7c** (red solid). TLC (SiO_2_), CH_2_Cl_2_/AcOEt 1:1; *R*_f_ = 0.28, 0.20, 0.13. Analytical HPLC (Knauer, A/B = 80/20–30/70 in 30 min, λ = 530 nm): *t*_R_ = 20.4 min (peak area 34%), 21.5 min (peak area 40%), 22.3 min (peak area 26%). **Compound 7a [isomer 5,5]**. ^1^H NMR (CD_3_CN, 400 MHz) δ 8.16 (dd, 2H, *J* = 1.5, 0.8; H-4,4′), 7.90 (dd, 2H, *J* = 8.0, 1.5; H-6,6′), 7.26 (dd, 2H, *J* = 8.0, 0.9; H-7,7′), 6.63 (d, 4H, *J* = 8.7), 6.59 (d, 4H, *J* = 2.4), 6.48 (dd, 4H, *J* = 8.7, 2.4), 5.28 (t, 4H, *J* = 8.7, NH), 3.89 (qd, 8H, *J* = 9.3, 6.8; CH_2_CF_3_). ^13^C{^1^H} NMR (CD_3_CN, 101 MHz) δ 169.7 (COOH), 154.2, 154.0, 151.1, 139.4, 130.5, 129.1, 126.9 (q, *J* = 276, CF_3_), 125.9, 125.5, 111.6, 109.85, 99.9, 90.6, 45.8 (q, *J* = 33, CH_2_CF_3_). HRMS (ESI-TOF) *m*/*z*: [M + H]^+^ calcd for C_50_H_31_F_12_N_4_O_6_ 1011.2046; found 1011.2040. *m*/*z*: [M + Na]^+^ calcd for C_50_H_30_F_12_N_4_O_6_Na 1033.1866; found 1033.1850. *m*/*z*: [M + 2H]^2+^ calcd for C_50_H_32_F_12_N_4_O_6_ 506.1060; found 506.1057. **Compound 7b [isomer 5,6′]**. ^1^H NMR (CD_3_CN, 400 MHz) δ 8.17 (dd, 1H, *J* = 1.5, 0.8; H-4), 8.09 (dd, 1H, *J* = 8.0, 0.7; H-4′), 7.90 (dd, 1H, *J* = 8.0, 1.4; H-6), 7.86 (dd, 1H, *J* = 8.0, 1.4; H-5′), 7.46 (dd, 1H, *J* = 1.4, 0.7; H-7′), 7.25 (dd, 1H, *J* = 8.0, 0.7; H-7), 6.81 (d, 2H, *J* = 8.8), 6.75 (d, 2H, *J* = 8.8), 6.71 (dd, 4H, *J* = 8.4, 2.4), 6.62 (dd, 2H, *J* = 8.8, 2.4), 6.59 (dd, 2H, *J* = 8.8, 2.4), 5.74 (br. s, 4H, NH), 3.96 (m, 8H, CH_2_CF_3_). ^1^H NMR (CD_3_OD, 400 MHz) δ 8.36 (m, 1H, H-4), 8.27 (d, 1H, *J* = 8.1, H-4′), 8.02 (dd, 1H, *J* = 8.1, 1.5; H-5′), 7.98 (dd, 1H, *J* = 8.0, 1.6; H-6), 7.60 (dd, 1H, *J* = 1.5, 0.6; H-7′), 7.39 (dd, 1H, *J* = 8.0, 0.6; H-7), 7.05 (d, 2H, *J* = 9.0), 7.00 (d, 2H, *J* = 9.0), 6.93 (dd, 4H, *J* = 7.2, 2.3), 6.82 (m, 4H), 4.12 (m, 8H, CH_2_CF_3_). ^13^C{^1^H} NMR (CD_3_OD, 101 MHz) δ 172.7 (COOH), 167.2, 167.0, 155.7, 154.2 (all C_q_), 136.6 (CH), 133.1 (CH), 131.1 (CH), 130.3 (CH), 129.7 (CH), 125.1 (q, *J* = 282, CF_3_), 128.9 (C_q_), 128.8 (C_q_), 128.4 (CH), 128.1 (C_q_), 127.5 (CH), 124.2 (C_q_), 113.6 (CH), 111.6 (C_q_), 97.3 (CH), 91.1 (C_q_), 89.4 (C_q_), 45.1 (m, CH_2_CF_3_). HRMS (ESI-TOF) *m*/*z*: [M + H]^+^ calcd for C_50_H_31_F_12_N_4_O_6_ 1011.2046; found 1011.2039. *m*/*z*: [M + Na]^+^ calcd for C_50_H_30_F_12_N_4_O_6_Na 1033.1866; found 1033.1853. *m*/*z*: [M + 2H]^2+^ calcd for C_50_H_32_F_12_N_4_O_6_ 506.1060; found 506.1054. **Compound 7c [isomer 6,6]**. ^1^H NMR (CD_3_CN, 400 MHz) δ 8.02 (dd, 2H, *J* = 8.0, 0.7; H-4,4′), 7.79 (dd, 2H, *J* = 8.0, 1.4; H-5,5′), 7.37 (dd, 2H, *J* = 1.4, 0.7; H-7,7′), 6.74 (d, 4H, *J* = 8.8; H-4″,5″ in xanthene), 6.68 (d, 4H, *J* = 2.4; H-1″,8″ in xanthene), 6.57 (dd, 4H, *J* = 8.8, 2.4; H-3″,6″ in xanthene), 5.69 (br. s, 4H, NH), 3.94 (m, 8H, CH_2_CF_3_). ^1^H NMR (CD_3_OD, 400 MHz) δ 8.32 (d, 2H, *J* = 7.9, H-4,4′), 7.95 (m, 2H, H-5,5′), 7.63 (dd, 2H, *J* = 1.4, H-7,7′), 7.19 (d, 4H, *J* = 9.2), 7.13 (d, 4H, *J* = 2.1), 7.00 (dd, 4H, *J* = 9.1, 2.2), 4.23 (q, 8H, *J* = 9, CH_2_CF_3_). ^13^C{^1^H} NMR (CD_3_OD, 101 MHz) δ 160.2, 159.9 (COOH), 136.2 (C_q_), 134.8 (CH), 134.2 (CH), 133.1 (CH), 132.6 (CH), 130.5, 128.4, 127.7, 124.9 (all C_q_), 124.8 (q, *J* = 280, CF_3_), 118.1 (CH), 116.3 (C_q_), 97.6 (CH), 92.4 (C_q_), 45.2 (q, *J* = 34, CH_2_CF_3_). HRMS (ESI-TOF) *m*/*z*: [M + 2H]^2+^ calcd for C_50_H_32_F_12_N_4_O_6_ 506.1060; found 506.1056.

#### Hydration of Acetylenes **7a–c** to Ketones **8a–c**

The reaction was carried out in two 20 mL Biotage microwave reaction vials. The mixture of acetylenes **7a**–**c** (100 mg, 0.10 mmol, 1.0 equiv) was placed in a vial, purged with Ar, and sealed with a septum and a cap. Under stirring at room temperature, the following reagents were added dropwise via syringes to each vial: propionic acid (300 μL, 297 mg, 4.07 mmol, 40.5 equiv), water (2.10 mL, 116 mmol, 1180 equiv), and CF_3_SO_3_H (4.50 mL, 7.70 g, 51 mmol, 518 equiv). The reaction mixtures were heated with stirring at 140 °C. After 1, 2, and 3 h, additional portions of CF_3_SO_3_H (0.50 mL each time) were added dropwise at 140 °C. After heating at 140 °C for 4 h, the reaction mixture was cooled down to 100 °C; water (1.0 mL), propionic acid (0.1 mL), and CF_3_SO_3_H (0.50 mL) were added; and heating was continued at 140 °C for 1 h. After 5 h, CF_3_SO_3_H (500 μL) was added at 140 °C and heating was continued. After 6 and 8 h, the reaction mixture was cooled to 100 °C, and further portions of water (1.0 mL), propionic acid (0.1 mL), and CF_3_SO_3_H (0.50 mL) were added. After each addition, heating at 140 °C was continued, and finally, the reaction mixture was heated at 140 °C overnight. The HPLC (LC-MS) analysis evidenced full conversion. The contents of two vials were carefully transferred with aqueous dioxane into a 1 L Erlenmeyer flask with saturated aqueous NaHCO_3_ (200 mL) and ethyl acetate (100 mL). The flask was cooled with ice water, and stirring was applied to avoid strong foaming. If pH of the aqueous layer was slightly basic or neutral, the organic layer was separated and the aqueous layer was extracted with ethyl acetate (3 × 25 mL). Combined organic solutions (OS1) were set aside; the aqueous phase was acidified to pH 5 by addition of 10% aq. citric acid and extracted with ethyl acetate (3 × 25 mL). These “second” organic solutions (OS2) were washed with saturated aq. NaHCO_3_. The combined OS1 and OS2 were washed with water (2 × 50 mL), brine (2 × 50 mL), dried over MgSO_4_, and concentrated. The residue was dissolved in MeCN (6 mL) and water (2 mL) and subjected to prep. RP-HPLC (in 2 portions). HPLC column Knauer 250 × 30 mm, MeCN/aqueous 50 mM NH_4_OAc buffer (pH 6.9) = 40:60–70:30 in 30 min, flow rate 45 mL/min, λ = 530 nm. The residue was dissolved in dioxane, filtered through a 0.2 μM PTFE membrane filter, frozen, and lyophilized to yield 163 mg (80%) of a dark red solid containing all four isomers of **8a**–**d**. Further separation (RP-HPLC) (see above) afforded four fractions of the individual regioisomers: 6CH_2_-6CO (**8d**, 41 mg, 20%); 5CH_2_-6CO (**8c**, 13 mg, 6.4%); 6CH_2_-5CO (**8b**, 35 mg, 17%); 5CH_2_-5CO (**8a**, 30 mg, 15%). In total, 119 mg (59%) of four regioisomers as red solids was isolated. TLC: CHCl_3_/MeOH/H_2_O = 35:30:2; *R*_f_ = 0.15 for all four isomers. Analytical HPLC (Interchim column 4.6 × 250 mm, MeCN/50 mM aq. NH_4_OAc buffer = 40:60–70:30% in 30 min, flow rate 1.2 mL/min, λ = 500 nm): *t*_R_ (6CH_2_-6CO, **8d**) = 26.6 min (peak area 98%), *t*_R_ (5CH_2_-6CO, **8c**) = 27.3 min (peak area 90%), *t*_R_ (6CH_2_-5CO, **8b**) = 28.4 min (peak area 99.5%), *t*_R_ (5CH_2_-5CO, **8a**) = 28 min (peak area 98%). **Compound 8a [5(CH**_**2**_**)-5(CO)]**. ^1^H NMR (CD_3_CN, 400 MHz) δ 8.60 (dd, 1H, *J* = 1.6, 0.7; H-4′ [CO]), 8.37 (dd, 1H, *J* = 8.1, 1.6; H-6′ [CO]), 7.86 (m, 1H, H-4 [CH_2_]), 7.64 (dd, 1H, *J* = 7.9, 1.6; H-6 [CH_2_]), 7.36 (dd, 1H, *J* = 8.0, 0.7; H-7′ [CO]), 7.18 (dd, 1H, *J* = 7.9, 0.7; H-7 [CH_2_]), 6.63–6.55 (m, 8H), 6.47 (ddd, *J* = 8.7, 3.2 and 3.4; 4H), 5.24 (2 × t, *J* = 7, 4H, NH), 4.68 (s, 2H, CH_2_CO), 3.88 (2 × dq, *J* = 7.0, 9.4; 8H, CH_2_CF_3_). ^13^C{^1^H} NMR (CD_3_CN, 101 MHz) δ 197.4 (CO), 170.5, 169.7 (COOH), 158.4, 153.9, 153.2, 151.1, 150.9, 139.8, (all C_q_), 138.6 (CH), 138.3 (C_q_), 136.2 (CH), 130.2 (2 × CH), 129.0, 128.6, 128.3, 127.1 (all C_q_), 126.9 (q, *J* = 282, CF_3_), 126.2 (CH), 125.9 (CH), 125.6 (C_q_), 125.1 (CH), 111.6 (CH), 110.0 (CH), 109.2 (CH), 100.0 (CH), 85.5 (C_q_O), 85.1 (C_q_O), 46.0 (CH_2_), 45.8 (2 ×q, *J* = 43, CH_2_CF_3_). HRMS (ESI-TOF) *m*/*z*: [M + H]^+^ calcd for C_50_H_33_F_12_N_4_O_7_ 1029.2152; found 1029.2148. *m*/*z*: [M + Na]^+^ calcd for C_50_H_32_F_12_N_4_O_7_Na 1051.1972; found 1051.1937. *m*/*z*: [M + 2H]^2+^ calcd for C_50_H_34_F_12_N_4_O_7_ 515.1112; found 515.1113. **Compound 8b [6(CH**_**2**_**)-5(CO)]**. ^1^H NMR (CD_3_CN, 400 MHz) δ 8.48 (dd, 1H, *J* = 1.6, 0.7; H-4 [CO]), 8.24 (dd, 1H, *J* = 8.1, 1.6; H-6 [CO]), 7.93 (dd, 1H, *J* = 7.9, 0.7; H-4 [CH_2_]), 7.56 (dd, 1H, *J* = 7.9, 1.4; H-5 [CH_2_]), 7.28 (dd, 1H, *J* = 8.1, 0.7; H-7 [CO]), 7.11 (dd, 1H, *J* = 1.4, 0.7; H-7 [CH_2_]), 6.61 (d, 2H, *J* = 8.7), 6.58–6.57 (m, 3H), 6.52 (m, 1H), 6.45 (ddd, *J* = 8.9, 6.7 and 2.4, 4H), 5.23 (2 × t, *J* = 7, 4H, NH), 4.56 (s, 2H, CH_2_CO), 3.87 (m, 8H, CH_2_CF_3_). ^13^C{^1^H} NMR (CD_3_CN, 101 MHz) δ 197.1 (CO), 170.5, 169.6 (COOH), 158.3, 154.9, 153.9, 151.1, 150.9, 144.2, 139.6, (all C_q_), 136.1 (CH), 133.2 (CH), 130.4 (CH), 130.3 (CH), 128.9 (C_q_), 128.3 (C_q_), 126.9 (q, *J* = 282, CF_3_), 126.8 (CH), 126.1 (CH), 125.7 (CH), 125.6, 111.6, 111.5, 110.0, 109.1 (all C_q_), 99.94 (CH), 99.90 (CH), 46.7 (CH_2_), 45.8 (2 × q, *J* = 34, CH_2_CF_3_). HRMS (ESI-TOF) *m*/*z*: [M + H]^+^ calcd for C_50_H_33_F_12_N_4_O_7_ 1029.2152; found 1029.2150. *m*/*z*: [M + Na]^+^ calcd for C_50_H_32_F_12_N_4_O_7_Na 1051.1972; found 1051.1932. *m*/*z*: [M + 2H]^2+^ calcd for C_50_H_34_F_12_N_4_O_7_ 515.1112; found 515.1110. **Compound 8c [5(CH**_**2**_**)-6(CO)]**. ^1^H NMR (CD_3_CN, 400 MHz) δ 8.26 (dd, 1H, *J* = 8.0, 1.4; H-4 [CO]), 8.09 (d, 1H, *J* = 8.1; H-3 [CO]), 7.88 (d, 1H, *J* = 1.1; H-7 [CO]), 7.75 (d, 1H, *J* = 1.5; H-7 [CH_2_]), 7.50 (dd, 1H, *J* = 8.0, 1.6; H-6 [CH_2_]), 7.08 (d, 1H, *J* = 7.9, 1.4; H-7 [CH_2_]), 6.63–6.49 (m, 8H), 6.45 (m, 4H), 5.27 (m, 4H, NH), 4.48 (s, 2H, CH_2_CO), 3.88 (m, 8H, CH_2_CF_3_). ^13^C{^1^H} NMR (CD_3_CN, 101 MHz) δ 198.0 (CO), 170.4, 169.7 (COOH), 154.7, 154.0, 153.9, 152.9, 151.1, 150.9, 143.8, 138.5 (all C_q_), 138.1 (CH), 131.8 (C_q_), 130.4 (CH), 130.2 (CH), 128.4 (C_q_), 128.3 (C_q_), 127.0 (C_q_), 126.5 (CH), 126.5 (q, *J* = 282, CF_3_), 125.9 (CH), 124.6 (CH), 124.4 (CH), 111.5 (2 × CH), 109.9 (C_q_), 109.4 (C_q_), 99.9 (CH), 46.2 (CH_2_), 45.8 (2 × q, *J* = 45, CH_2_CF_3_). ^19^F NMR (CD_3_CN, 376 MHz) δ −72.85 (t, *J* = 9.3), −72.84 (t, *J* = 9.3). HRMS (ESI-TOF) *m*/*z*: [M + H]^+^ calcd for C_50_H_33_F_12_N_4_O_7_ 1029.2152; found 1029.2127. *m*/*z*: [M + Na]^+^ calcd for C_50_H_32_F_12_N_4_O_7_Na 1051.1972; found 1051.1970. *m*/*z*: [M + 2H]^2+^ calcd for C_50_H_34_F_12_N_4_O_7_ 515.1112; found 515.1109.

### Compound **8d** [6(CH_2_)-6(CO)]

^1^H NMR (CD_3_CN, 400 MHz) δ 8.09 (dd, 1H, *J* = 8.0, 1.4; H-5 [CO]), 8.00 (dd, 1H, *J* = 8.0, 0.8; H-4 [CO]), 7.83 (dd, 1H, *J* = 7.9, 0.7; H-4 [CH_2_]), 7.69 (m, 1H, H-7 [CO]), 7.41 (dd, 1H, *J* = 7.9, 1.4; H-5′ [CH_2_]), 6.91 (m, 1H, H-7 [CH_2_]), 6.56 (m, 4H), 6.48 (dd, *J* = 8.7, 2.3; 4H), 6.41 (dt, *J* = 8.7, 2.6; 4H), 5.22 (m, 4H, NH), 4.31 (s, 2H, CH_2_CO), 3.88 (m, 8H, CH_2_CF_3_). ^13^C{^1^H} NMR (CD_3_CN, 101 MHz) δ 197.9 (CO), 170.4, 169.6 (COOH), 154.7, 154.6, 154.0, 153.8, 151.0, 150.8, 144.1, 143.7 (all C_q_), 133.1 (CH), 131.7 (C_q_), 130.8, 130.4 (CH), 130.3 (CH), 128.3 (C_q_), 126.9 (C_q_), 126.9 (q, *J* = 282, CF_3_), 126.7 (CH), 126.4 (CH), 125.7 (CH), 125.6, 125.3 (CH), 111.51 (CH), 111.45 (CH), 109.9 (C_q_), 109.4 (C_q_), 99.9 (CH), 85.7 (C_q_O), 84.3 (C_q_O), 47.0 (CH_2_), 45.8 (2 × q, *J* = 34, CH_2_CF_3_). HRMS (ESI-TOF) *m*/*z*: [M + H]^+^ calcd for C_50_H_33_F_12_N_4_O_7_ 1029.2152; found 1029.2122. *m*/*z*: [M + Na]^+^ calcd for C_50_H_32_F_12_N_4_O_7_Na 1051.1972; found 1051.1942. *m*/*z*: [M + 2H]^2+^ calcd for C_50_H_34_F_12_N_4_O_7_ 515.1112; found 515.1109.

#### α-Diazoketones **1a–c**

A solution of the mixture containing **8a**, **8b**, **8c**, and **8d** (128 mg, 0.125 mmol, 1.0 equiv) and *p*-toluene sulfonyl azide (42 mg, 0.22 mmol, 1.7 equiv) in MeCN (2.6 mL; purged with argon) were introduced into an oven-dried 10 mL vial filled with argon. After cooling in an ice bath, a solution of DBU (36 mg, 0.24 mmol) in MeCN was added dropwise via a syringe within 10 min. The yellow color changed to cherry red in the course of DBU addition. The reaction mixture was kept with stirring at 0 °C for 2–3 h and then stirred at rt for 8–20 h. AcOEt (5 mL) and H_2_O (5 mL) were added, and the reaction mixture was stirred for 5 min. The organic phase was separated, the aqueous phase diluted with H_2_O (10 mL), and extracted with ethyl acetate (3 × 15 mL). The combined organic solutions were washed with H_2_O (10 mL), and the phases separated. The combined aqueous solutions (25 mL) were reextracted with ethyl acetate (2 × 10 mL). The combined organic solutions were shaken with saturated NaHCO_3_ (2 × 10 mL), and the combined aqueous NaHCO_3_ solutions (20 mL) were reextracted with ethyl acetate (10 mL). All organic phases were combined and kept for further workup. The combined aq. phases were neutralized with 10% aq. citric acid (ca. 20 mL) to pH ∼5 and extracted with ethyl acetate (2 × 10 mL). All combined organic solutions were washed with aq. NaHCO_3_ (10 mL), saturated brine (50 mL), dried over MgSO_4_, and concentrated under exclusion of light and atmospheric oxygen. The residue was dissolved in a mixture of MeCN (3 mL) and H_2_O (1 mL) and subjected to preparative RP-HPLC. Knauer column (see the General remarks section), MeCN/H_2_O + 50 mM aq. NH_4_OAc buffer (pH 6.9) = 40:60–70:30% in 40 min, flow rate 45 mL/min, λ = 510 nm. The fractions containing individual products were pooled and lyophilized separately. Each isomer was dissolved in 1,4-dioxane, filtered through a 0.2 μM PTFE membrane filter, frozen, and lyophilized to yield four isomers as red solids. 5(N_2_)-6(CO) (**1c**), 4.8 mg (3.7%); 6(N_2_)-6(CO) (**1d**), 16.0 mg (12%); 6(N_2_)-5(CO) (**1b**), 22.5 mg (17%); 5(N_2_)-5(CO) (**1a**), 9.3 mg (7%). In total, 52.6 mg (40%) of diazoketones were obtained. TLC (RP-18 F_254_), eluent: MeCN/aq. AcONH_4_ buffer (50 mM), 7/3. 5(N_2_)-6(CO) **1c**: *R*_f_ 0.18; 6(N_2_)-6(CO) **1d**: *R*_f_ 0.25; 6(N_2_)-5(CO) **1b**: *R*_f_ 0.18; 5(N_2_)-5(CO), **1a**, *R*_f_ 0.15. Analytical HPLC (Interchim column 250 × 4.6 mm, MeCN/aq. 50 mM NH_4_AcO buffer = 40:60–70:30 in 30 min, flow rate 1.2 mL/min): *t*_R_ 27.1 min (**1c** 5(N_2_)-6(CO), peak area 94%); *t*_R_ = 27.2 min (**1d**, 6(N_2_)-6(CO), peak area 96%); *t*_R_ = 27.7 min (**1b**, 6(N_2_)-5(CO), peak area 96%); *t*_R_ = 28.0 min (**1a**, 5(N_2_)-5(CO), peak area 88%). Analytical HPLC (Phenomenex Kinetex C18, 5 μM, 250 × 4.6 mm, MeCN/aq. 0.1% HCOOH in both components = 20:80–80:20 in 20 min, flow rate 1.2 mL/min, λ = 508 nm): *t*_R_ = 11.6 min (5(N_2_)-6(CO), **1c)**; *t*_R_ = 12.4 min (6(N_2_)-6(CO), **1d**); *t*_R_ = 11.8 min (6(N_2_)-5(CO), **1b**); *t*_R_ = 12.1 min (5(N_2_)-5(CO), **1a**).

### 1c [5(N_2_)-6(CO)] (isomer 1)

^1^H NMR (CD_3_CN, 400 MHz) δ 8.09 (d, 1H, *J* = 1.7, H-4), 8.07 (dd, 1H, *J* = 7.9, 0.8, H-4′), 7.92 (dd, 1H, *J* = 7.9, 1.4; H-5′), 7.73 (dd, 1H, *J* = 8.2, 1.8; H-6), 7.56 (dd, 1H, *J* = 1.3, 0.8; H-7′), 7.20 (dd, 1H, *J* = 8.2, 0.7; H-7), 6.62–6.52 (m, 8H), 6.46 (m, 4H), 5.24 (2 × t, 4H, *J* = 7.0, NH), 3.87 (m, 8H, CH_2_CF_3_). ^13^C NMR (CD_3_CN, 101 MHz) δ 188.1 (CO), 169.7 (COOH), 154.2, 154.1, 154.0, 152.6, 151.1, 151.0, 145.2 (all C_q_), 133.0 (CH), 130.8 (C_q_), 130.2 (CH), 129.9 (CH), 128.4 (C_q_), 128.3 (C_q_), 127.0 (q, *J* = 282, CF_3_), 125.4 (CH), 122.8 (CH), 111.6 (CH), 109.6 (C_q_), 99.9 (CH), 85.8 (C_q_), 85.3 (C_q_), 45.4 (q, *J* = 45, CH_2_CF_3_). ^19^F NMR (CD_3_CN, 376 MHz) δ −72.86 (t, *J* = 9.4), −72.85 (t, *J* = 9.4). HRMS (ESI-TOF) *m*/*z*: [M + H]^+^ calcd for C_50_H_31_F_12_N_6_O_7_ 1055.2057; found 1055.2061. *m*/*z*: [M + Na]^+^ calcd for C_50_H_30_F_12_N_6_O_7_Na 1077.1877; found 1077.1860. *m*/*z*: [M + 2H]^2+^ calcd for C_50_H_32_F_12_N_6_O_7_ 528.1065; found 528.1058.

### 1d [6(N_2_)-6(CO)] (isomer 2)

^1^H NMR ([D_4_]MeOH, 400 MHz) δ 8.11 (dd, 1H, *J* = 8.2, 2.6; H-4), 8.06 (dd, 1H, *J* = 8.3, 2.5, H-4′), 7.81 (d, 1H, *J* = 7.9; H-5), 7.63 (d, 1H, *J* = 7.4; H-5′), 7.35 (s, 1H, H-7), 7.22 (s, 1H, H-7′), 6.85 (m, 2H), 6.77 (m, 2H), 6.69 (m, 4H) 6.65–6.59 (m, 4H), 4.00 (m, 8H, CH_2_CF_3_). ^13^C NMR (CD_3_CN, 101 MHz) δ 188.8 (CO), 171.1 (COOH), 171.0 (COOH), 157.2, 156.4, 155.7, 155.6, 155.5, 155.1, 154.9, 147.5, 143.9, 142.4, 137.7, 137.6, 133.8 (all C_q_), 132.0 (CH), 131.7 (C_q_), 130.7 (CH), 128.6 (CH), 128.0 (C_q_), 127.5 (C_q_), 125.3 (C_q_), 125.2 (CH), 125.1 (q, *J* = 282, CF_3_), 114.4, (CH), 113.5 (CH), 112.6 (C_q_), 111.7 (C_q_), 111.6 (C_q_), 99.7 (CH), 98.4 (CH), 45.3 (q, *J* = 45, CH_2_CF_3_). ^19^F NMR ([D_4_]MeOH, 376 MHz) δ −71.8 ÷ −74.7 (m). HRMS (ESI-TOF) *m*/*z*: [M + H]^+^ calcd for C_50_H_31_F_12_N_6_O_7_ 1055.2057; found 1055.2042. *m*/*z*: [M + Na]^+^ calcd for C_50_H_30_F_12_N_6_O_7_Na 1077.1877; found 1077.1850. *m*/*z*: [M + 2H]^2+^ calcd for C_50_H_32_F_12_N_6_O_7_ 528.1065; found 528.1056.

### 1b [6(N_2_)-5(CO)] (isomer 3)

^1^H NMR ([D_4_]MeOH, 400 MHz) δ 8.23 (d, 1H, *J* = 1.5; H-4), 8.14 (d, 1H, *J* = 8.3, H-4′), 7.93 (dd, 1H, *J* = 7.9, 1.6; H-6), 7.86 (dd, 1H, *J* = 8.2, 1.7; H-5′), 7.44 (d, 1H, *J* = 1.3; H-7′), 7.29 (d, 1H, *J* = 7.9, H-7), 6.88 (m, 6H), 6.77 (m, 2H), 6.68 (m, 4H), 4.07/3.96 (2 × t, 8H, *J* = 9.2; CH_2_CF_3_). ^13^C NMR (CD_3_CN, 101 MHz) δ 189.7 (CO), 169.9 (COOH), 156.0, 155.4, 155.3, 153.6, 153.5, 144.9, 144.5, 138.9, 134.3, 131.9 (all C_q_), 131.2 (CH), 130.9 (C_q_), 129.9 (CH), 127.5 (CH), 127.6 (CH), 126.5 (CH), 125.7 (q, *J* = 281, CF_3_), 123.4 (CH), 113.3 (C_q_), 112.5 (C_q_), 111.1 (C_q_), 110.9 (C_q_), 97.1 (CH), 96.9 (CH), 45.4/45.2 (CH_2_CF_3_), ^19^F NMR ([D_4_]MeOH, 376 MHz) δ −73.4 (t, *J* = 9.2), −73.5 (t, *J* = 9.2). HRMS (ESI-TOF) *m*/*z*: [M + H]^+^ calcd for C_50_H_31_F_12_N_6_O_7_ 1055.2057; found 1055.2052. *m*/*z*: [M + Na]^+^ calcd for C_50_H_30_F_12_N_6_O_7_Na 1077.1877; found 1077.1860. *m*/*z*: [M + 2H]^2+^ calcd for C_50_H_32_F_12_N_6_O_7_ 528.1065; found 528.1057.

### 1a [5(N_2_)-5(CO)] (isomer 4)

^1^H NMR ([D_4_]MeOH, 400 MHz) δ 8.24(d, 1H, *J* = 1.4; H-4), 8.08 (d, 1H, *J* = 1.8, H-4′), 8.01 (dd, 1H, *J* = 7.9, 1.7; H-6), 7.93 (dd, 1H, *J* = 8.1, 1.9; H-6′), 7.39 (d, 1H, *J* = 7.9; H-7), 7.34 (d, 1H, *J* = 8.1, H-7′), 7.00 (d, 2H, *J* = 9), 6.94 (d, 2H, *J* = 9), 6.87 (m, 4H), 6.75 (m, 4H), 4.05 (m, CH_2_CF_3_). ^13^C NMR (CD_3_CN, 101 MHz) δ 187.7 (CO), 170.4 (COOH), 169.4 (COOH), 156.2, 156.1, 156.3, 156.0, 154.7, 154.6, 154.5, 153.4, 144.4, 139.7, 139.2, 135.1, 134.4 (all C_q_), 132.2 (CH), 131.6 (C_q_), 131.2 (CH), 130.3 (C_q_), 130.2 (C_q_), 128.8 (CH), 128.7 (CH), 127.2 (CH), 126.0, 124.9 (q, *J* = 282, CF_3_), 114.8 (CH), 113.5 (C_q_), 111.63 (C_q_), 111.60 (C_q_), 96.8 (CH), 45.2 (CH_2_CF_3_), ^19^F NMR ([D_4_]MeOH, 376 MHz) δ −73.44 (t, *J* = 9.1), −73.43 (t, *J* = 9.1). HRMS (ESI-TOF) *m*/*z*: [M + H]^+^ calcd for C_50_H_31_F_12_N_6_O_7_ 1055.2057; found 1055.2045. *m*/*z*: [M + Na]^+^ calcd for C_50_H_30_F_12_N_6_O_7_Na 1077.1877; found 1077.1840. *m*/*z*: [M + 2H]^2+^ calcd for C_50_H_32_F_12_N_6_O_7_ 528.1065; found 528.1054.

## Supporting Information Available

Photolysis of azibenzil (**11**) and diazoketones **1a–d**, HPLC traces, and NMR spectra of compounds **6a,b**, **7a–c**, **8a–d**, and **1a–d** (Figures S1–S5) (PDF)
