# Supplementary material for: Bis-Rhodamines Bridged with a Diazoketone Linker: Synthesis, Structure, and Photolysis
Source: J Org Chem. 2021 Dec 17;87(1):56–65. doi: 10.1021/acs.joc.1c01721 (PMC8749961; doi:10.1021/acs.joc.1c01721)

Supporting Information

Bis-rhodamines bridged with a diazoketone linker: synthesis, structure and photolysis

Heydar Shojaei,[a] Mariano L. Bossi,[b] Vladimir N. Belov,[a,*] Stefan W. Hell[a,b]

[a] Department of NanoBiophotonics, Max Planck Institute for Biophysical Chemistry, 37077 Göttingen, Germany
[b] Department of Optical Nanoscopy, Max Planck Institute for Medical Research, 69120 Heidelberg, Germany

*vladimir.belov@mpibpc.mpg.de

Table of Contents

Photolysis of azibenzil (**11**) and diazoketones **1a-d** – Figures S1-S5 S3

Regioisomeric bromorhodamine **6a** (5′-Br) and **6b** (6′-Br) S7

Bis(rhodamine) acetylenes **7a**, **7b** and S12

Bis-rhodamine ethanones **8a** [5(CH_2),5(CO)], **8b** [6(CH_2),5(CO)], **8c** [5(CH_2),6(CO)], **8d** [6(CH_2),6(CO)] S20

HPLC traces of bis-rhodamine diazoketones **1a** [5(N_2),5(CO)] (isomer 4), **1b** [6(N_2),5(CO)] (isomer 3), **1c** [5(N_2),6(CO)] (isomer 1) and **1d** [6(N_2),6(CO)] (isomer 2) S28

NMR Spectra of compound **1a** [5(N_2),5(CO)] (isomer 4) S30

NMR Spectra of compound **1b** [6(N_2),5(CO)] (isomer 3) S33

NMR Spectra of compound **1c** [5(N_2),6(CO)] (isomer 1) S36

NMR Spectra of compound **1d** [6(N_2),6(CO)] (isomer 2) S38

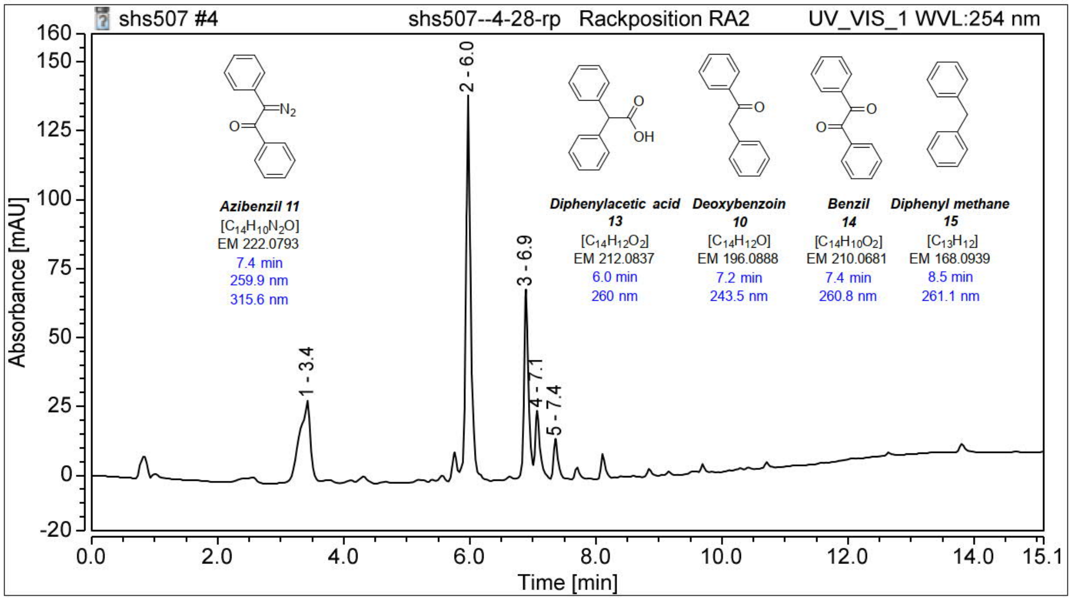

Figure S1. HPLC analysis of the reaction mixture: azibenzil (**11**) (0.1 mM) in acetonitrile (80% v/v) and aqueous HEPES buffer (20%, v/v) at pH 6.5 was irradiated with 365 nm light providing full conversion of the starting material.

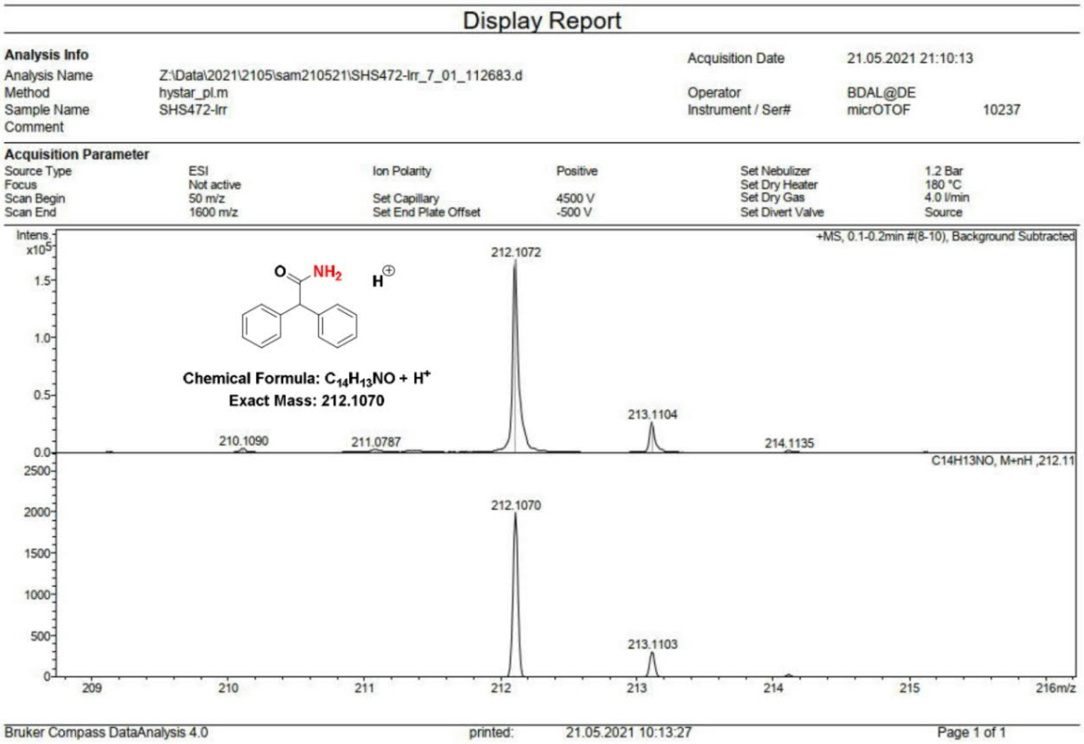

Figure S2. HR-MS of the reaction solution obtained after photolysis of azibenzil (**11**, $PhCOCN_2Ph$) solutions in aqueous acetonitrile (80% acetonitrile, 20% water, v/v) with $HCOONH_4$ buffer (pH 7.3). The full conversion to a new substance with the same retention time, but without absorption maximum at 319 nm, was observed (see Figure 2 in the main text).

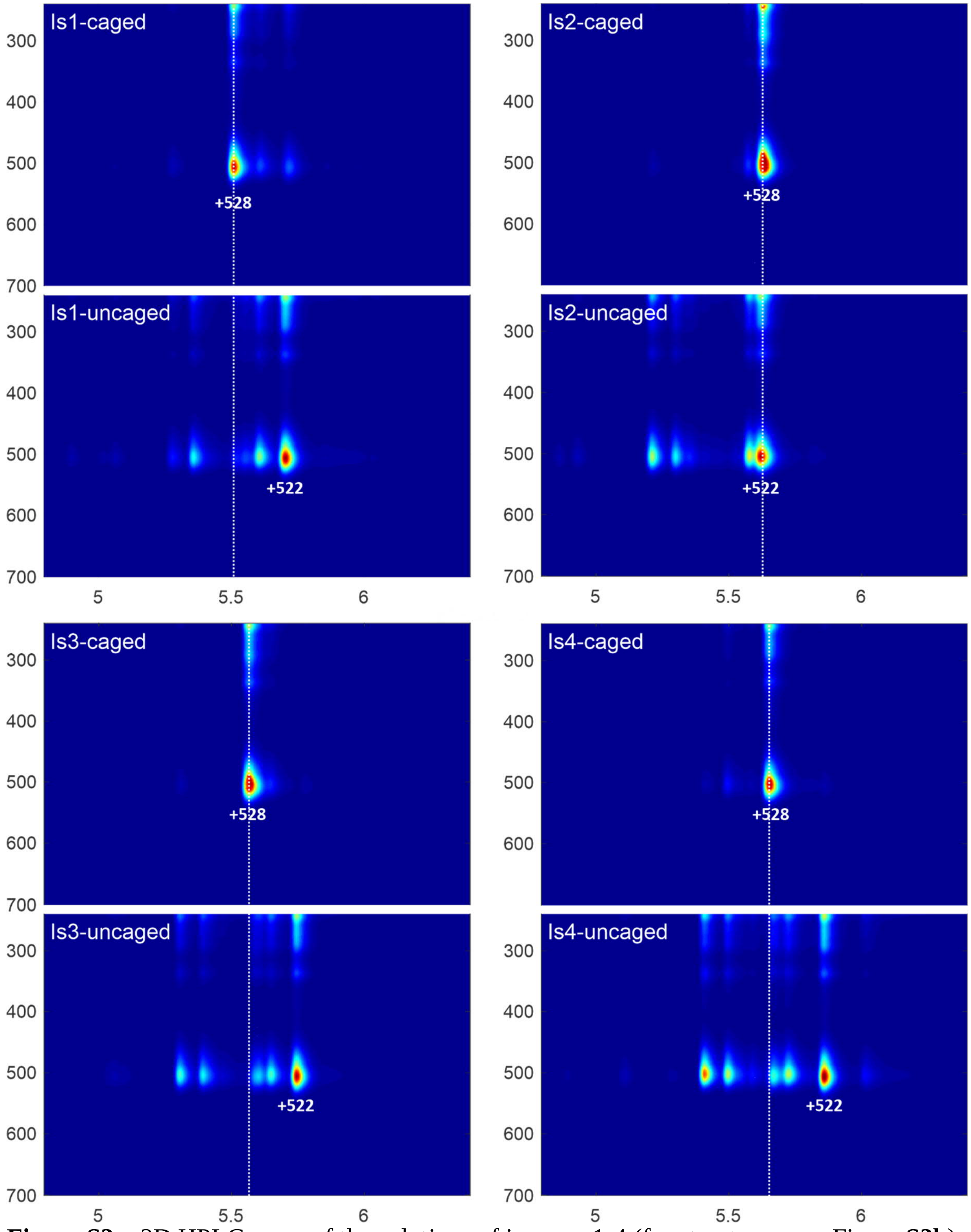

Figure S3a. 2D HPLC maps of the solutions of isomers 1-4 (for structures, see Figure **S3b**), before and after irradiation in MeCN (80% v/v) and aqueous 10 mM ammonium formate buffer (20% v/v; pH = 7.4), with 365 nm light, upon complete conversion. The *m/z* values of the main peaks $[M+2H]^{2+}$ are given for the starting materials (*m/z* 528) and the main reaction products (*m/z* 522).

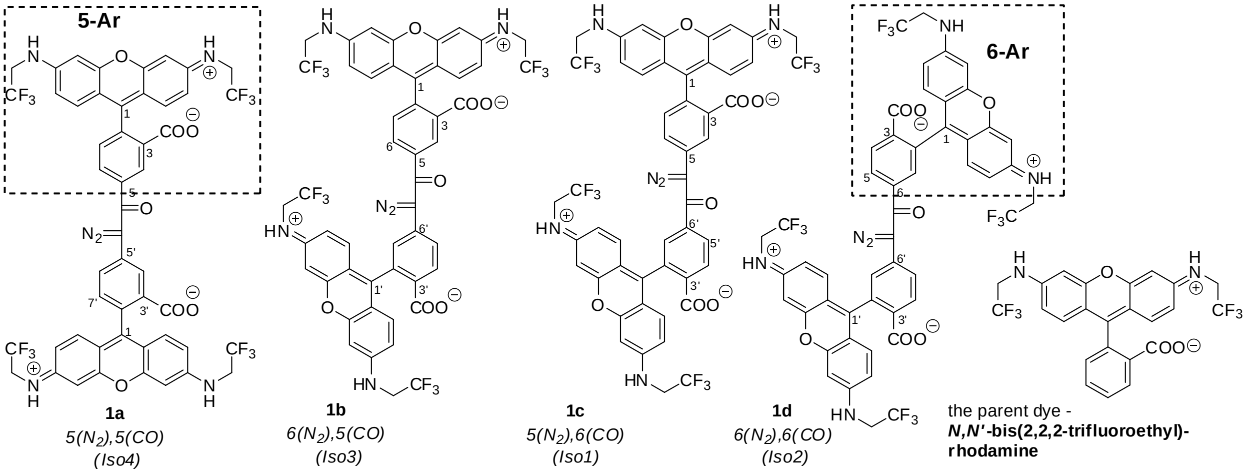

Figure S3b. Structures of compounds **1a, 1b, 1c** and **1d** (isomers 4, 3, 1 and 2, respectively), and the parent rhodamine dye.

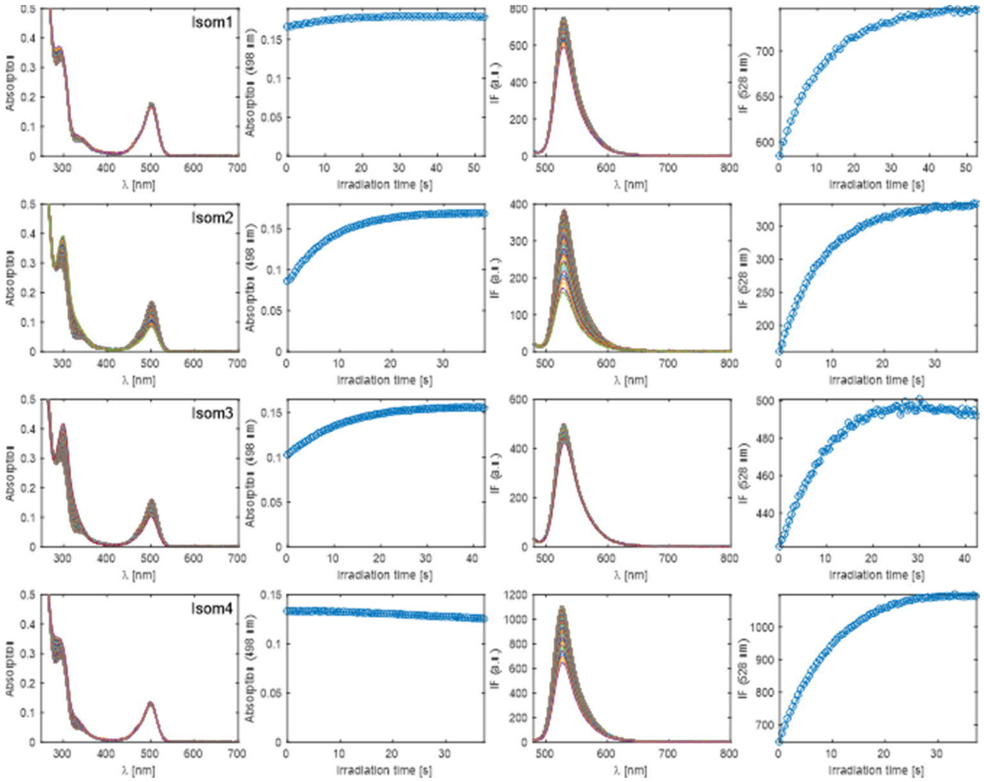

Figure S4a. Irradiation of isomers 1-4 (12 µM, for structures, see Figure S3b) in MeCN (80% v/v) and aqueous 10 mM ammonium formate buffer, pH = 7.4 (20% v/v). The samples were continuously stirred and kept at 20°C during irradiation with 365 nm light. Absorption at 498 nm and emission at 528 nm (λ_{EX} = 470 nm) were monitored and read out at fixed time intervals.

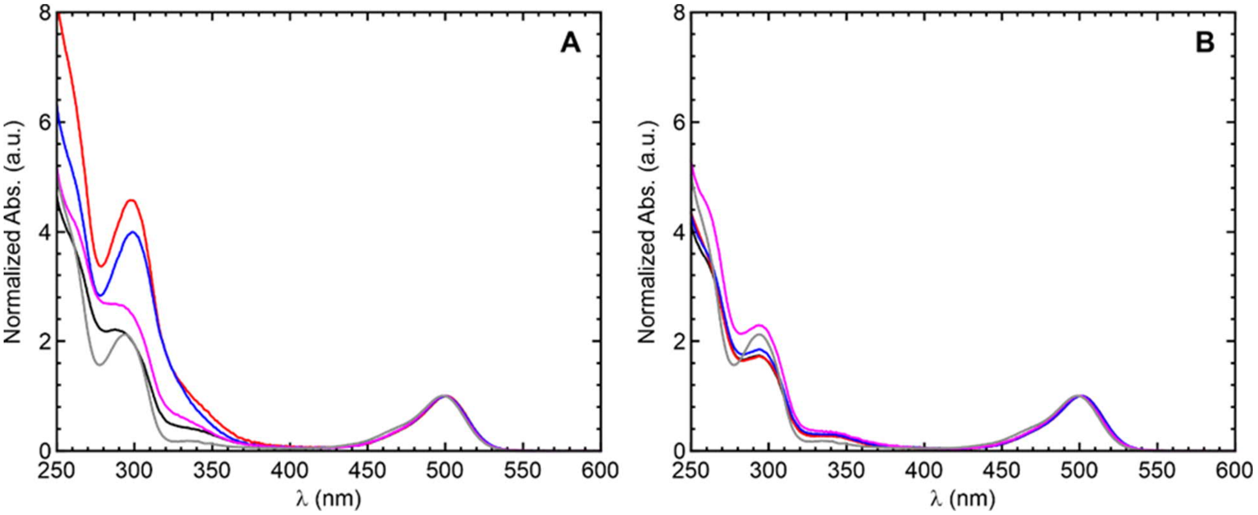

Figure S4b. Absorption spectra (normalized to the intensity of the band with the maximum at 498 nm) of compounds **1a** (purple), **1b** (blue), **1c** (black), **1d** (red) and the parent *N*,*N'*-bis(2,2,2-trifluoroethyl)rhodamine (grey) before (A) and after (B) photoconversion by irradiation with 365 nm source. For structures, see Figure S3b. Solvent: aqueous acetonitrile (80% acetonitrile, 20% water, v/v) with addition of $HCOONH_4$ buffer (pH 7.3).

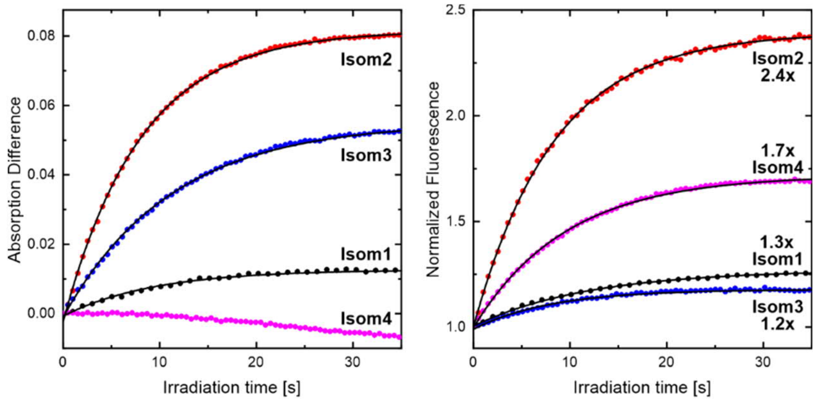

Figure S5. Normalized fluorescence (right) and absorption (left) transients during irradiation of isomers 1-4 (**1a-d**, 12 µM) with 365 nm light, as solutions in MeCN (80%) and aqueous 10 mM ammonium formate buffer; pH = 7.4 (20% v/v). Samples were continuously stirred and kept at 20°C during irradiation. Absorption at 498 nm and emissionat at 528 nm (λ_{EX} = 470 nm) were monitored at read out at fixed time intervals. The emission intensities were divided (normalized) by the corresponding initial value for each compound, and the initial absorbance was subtracted for each sample.

Bromorhodamines **6a** (5′-Br) and **6b** (6′-Br)

**Probe :**	**shs475-5-iso3**				
**Lösungsmittel :**	**MeCN/H2O**	**Aufgabemenge:**	**3.0 µl**		
**Säule:**	Phenomenex Kinetex C18 2.6 µm	**Länge:**	75 mm	**iO :**	3.0 mm
**Fluß (ml / Min) :**	**0.5**	**Temperatur :**	**25.0**		
**Detektor:**	DAD-3000	**Pumpe:**	HPG-3200SD	**Sampler:**	WPS-3000
**Laufmittel:**	**A = Acetonitril 0.1% FA**			**B = Wasser 0.1% FA**	
**Gradient:**	A 20.0 % B 80.0 %	**---->**	A 100.0 % B 0.0 %	T = 10 Min.	

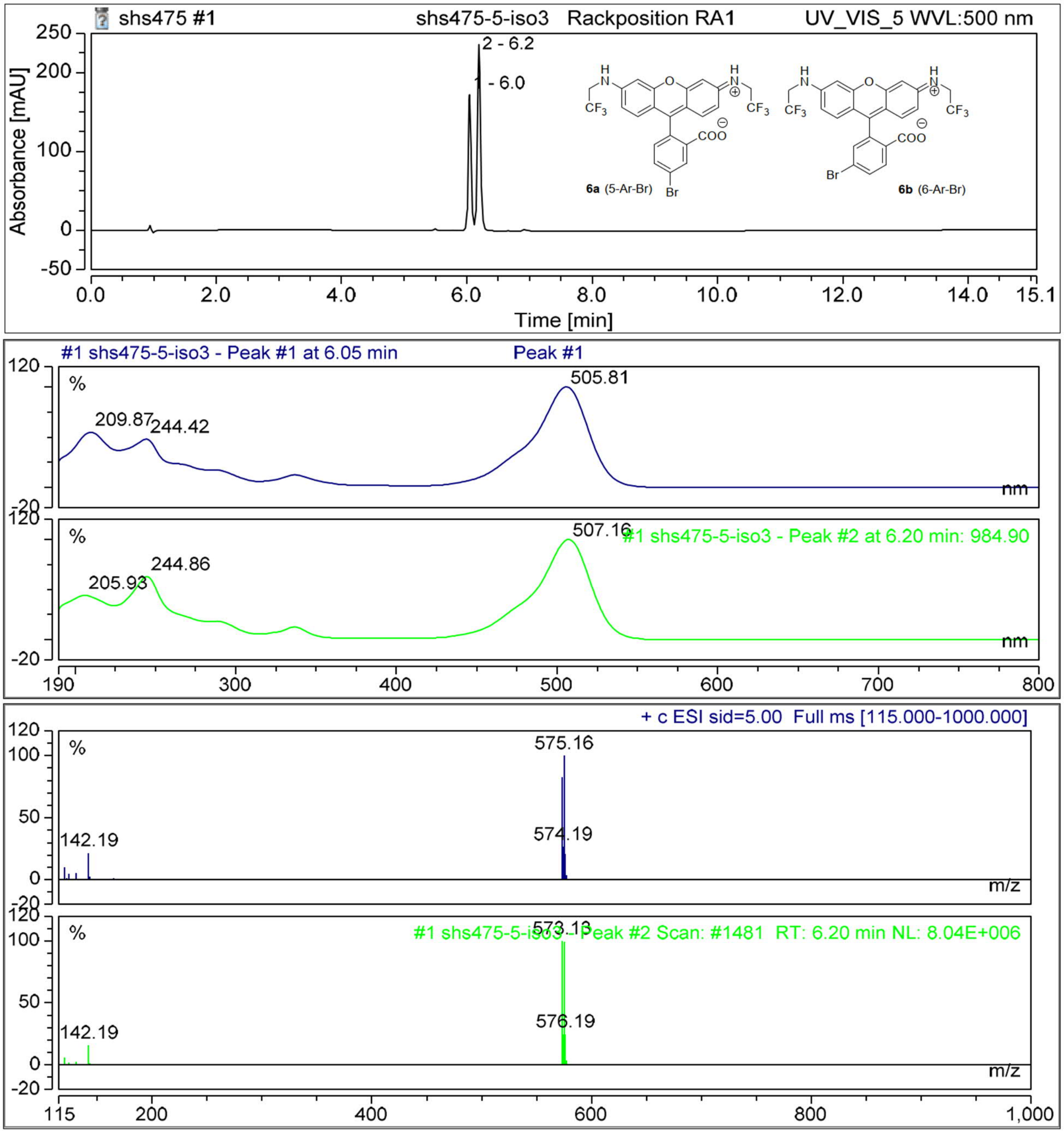

^{1}H NMR (400 MHz, CD_3CN) δ 8.11 (d, J = 1.8 Hz, 0H), 7.89 – 7.75 (m, 1H), 7.41 (d, J = 1.6 Hz, 0H), 7.11 (d, J = 8.2 Hz, 0H), 6.64 – 6.55 (m, 4H), 6.46 (dt, J = 8.7, 2.8 Hz, 2H), 5.27 (t, J = 6.9 Hz, 2H), 3.96 – 3.78 (m, 4H).

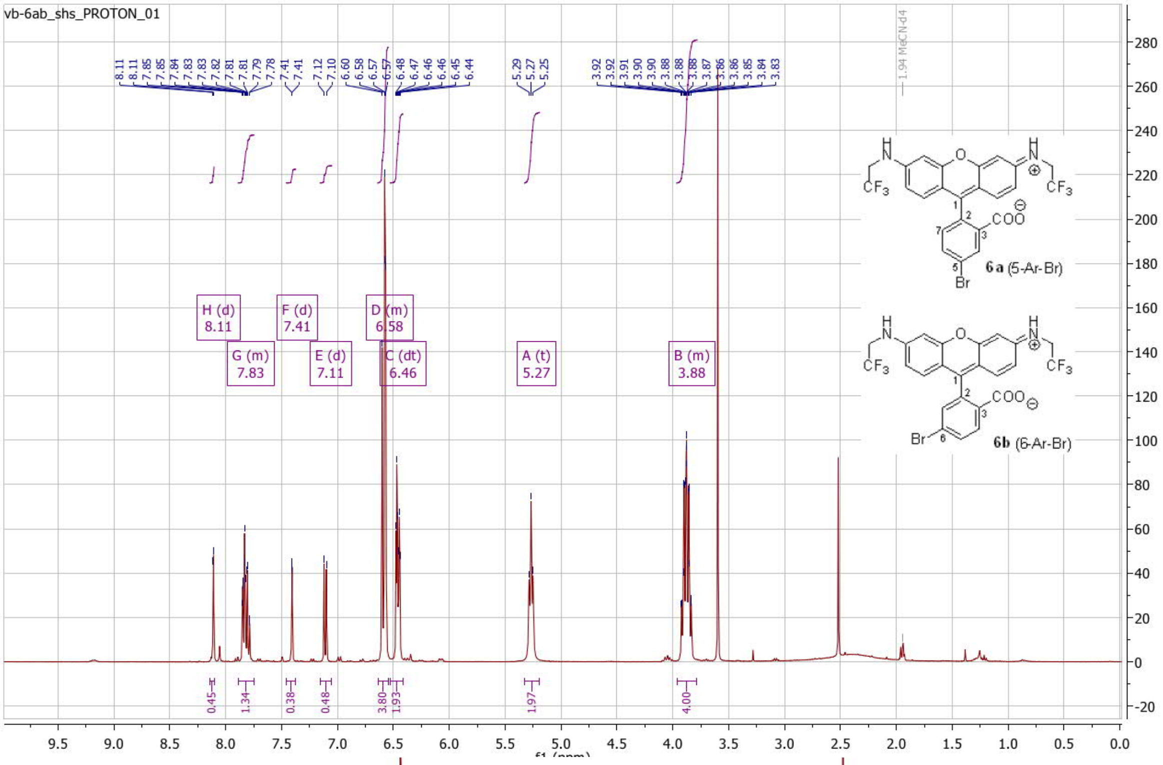

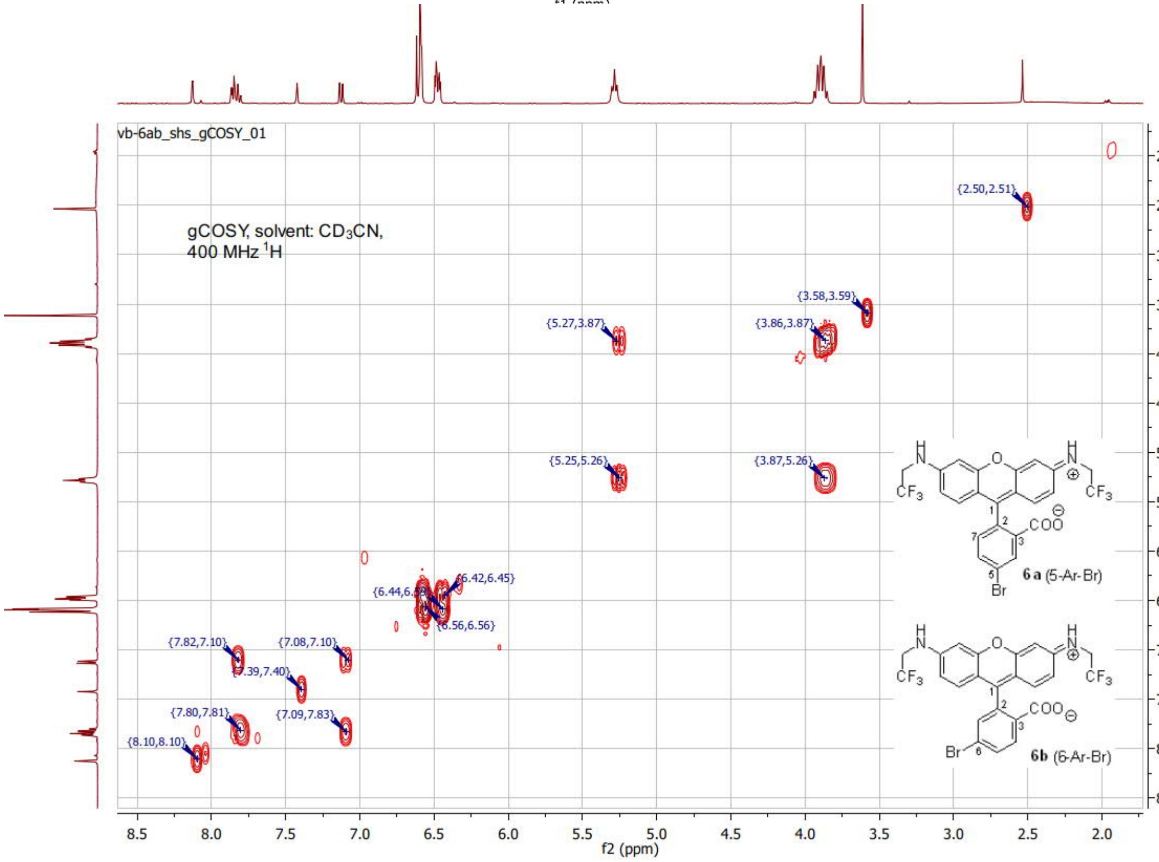

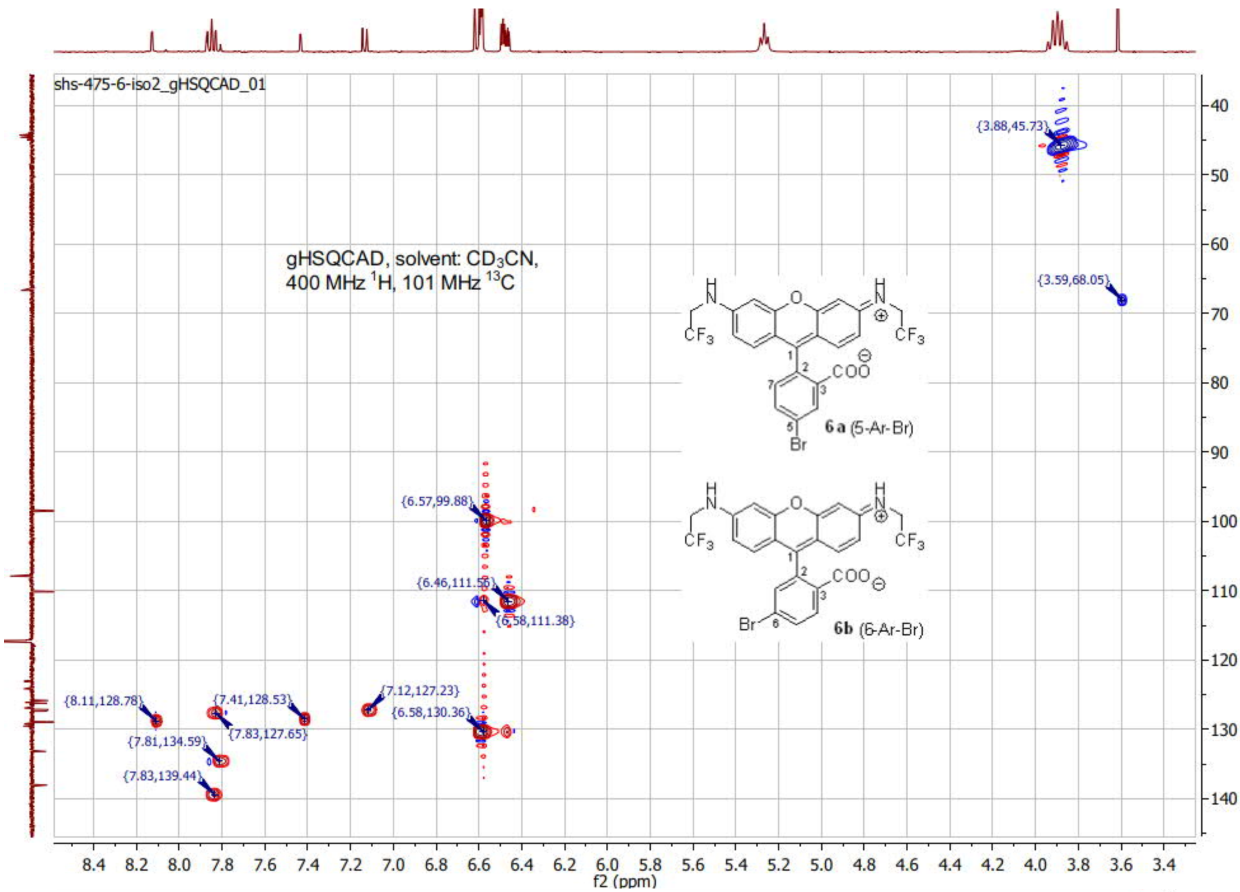
shs-475-6-iso2_gHSQCAD_01
gHSQCAD, solvent: CD_3CN,
400 MHz ^{1}H, 101 MHz ^{13}C
{3.88,45.73}
{3.59,68.05}
{6.57,99.88}
{6.46,111.55}
{6.58,111.38}
{8.11,128.78}
{7.41,128.53}
{7.83,127.65}
{7.12,127.23}
{6.58,130.36}
{7.81,134.59}
{7.83,139.44}
6a (5-Ar-Br)
6b (6-Ar-Br)
f2 (ppm)

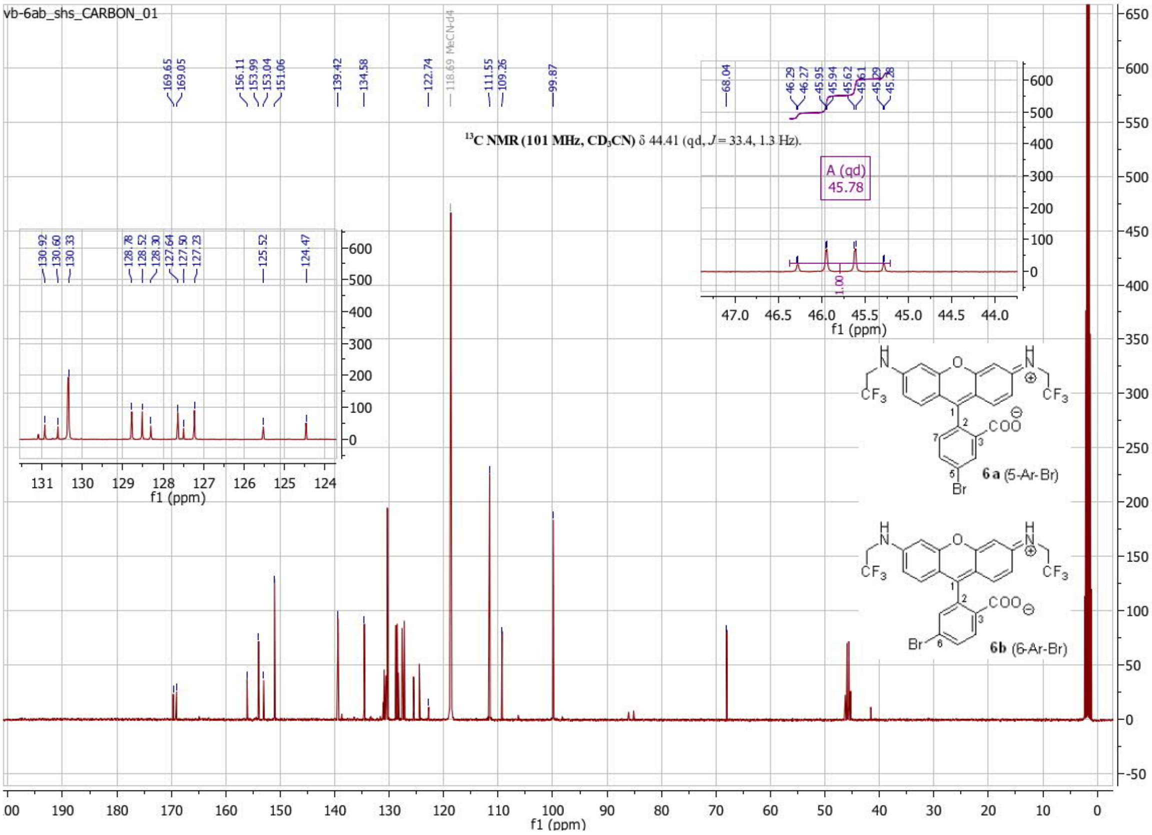
vb-6ab_shs_CARBON_01
169.65
169.05
156.11
153.99
153.04
151.06
139.42
134.58
122.74
118.69 MeCN-d4
111.55
109.26
99.87
68.04
46.29
46.27
45.95
45.94
45.62
45.61
45.29
45.28
^{13}C NMR (101 MHz, CD_3CN) δ 44.41 (qd, J = 33.4, 1.3 Hz).
A (qd)
45.78
130.92
130.60
130.33
128.78
128.52
128.30
127.64
127.50
127.23
125.52
124.47
6a (5-Ar-Br)
6b (6-Ar-Br)
f1 (ppm)

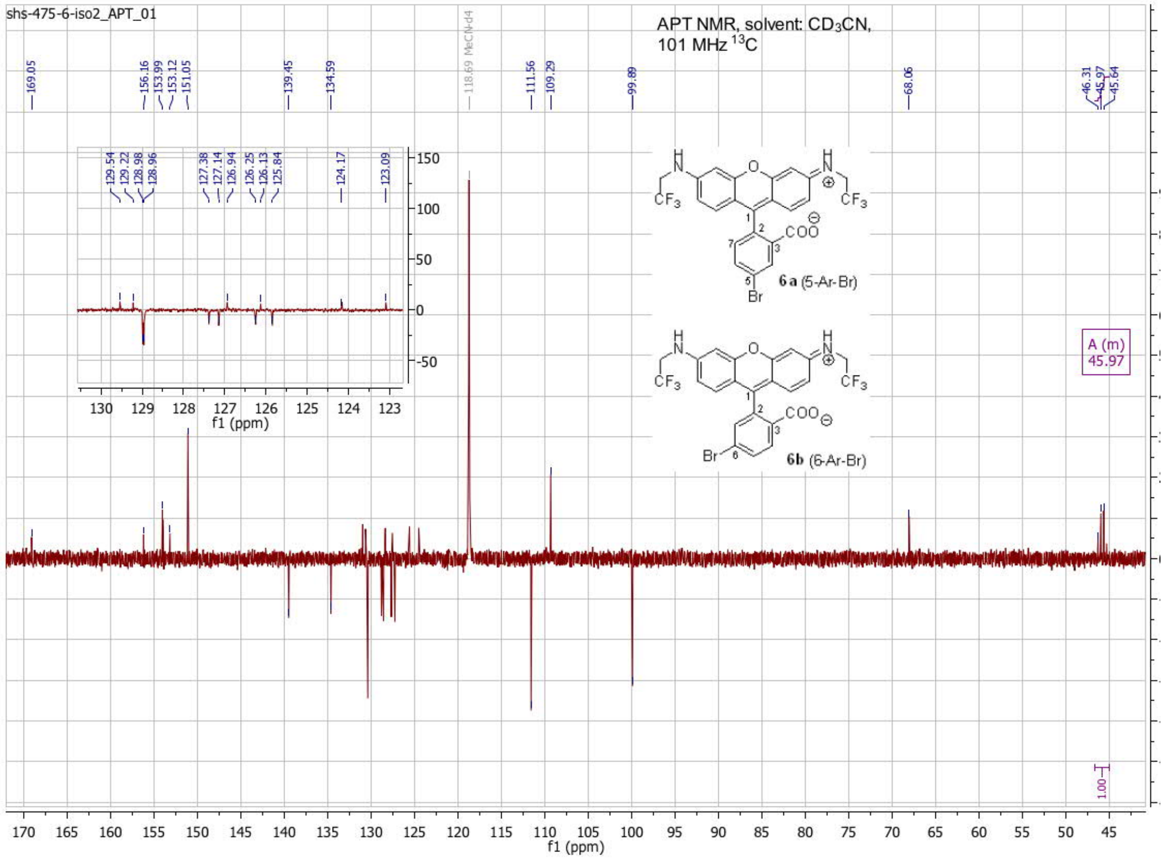

shs-475-6-iso2_APT_01
APT NMR, solvent: CD3CN,
101 MHz 13C
169.05
156.16
153.99
153.12
151.05
139.45
134.59
118.69 MeCN-d4
111.56
109.29
99.89
68.06
46.31
45.97
45.64
129.54
129.22
128.98
128.96
127.38
127.14
126.94
126.25
126.13
125.84
124.17
123.09
6a (5-Ar-Br)
6b (6-Ar-Br)
A (m)
45.97
1.00
f1 (ppm)

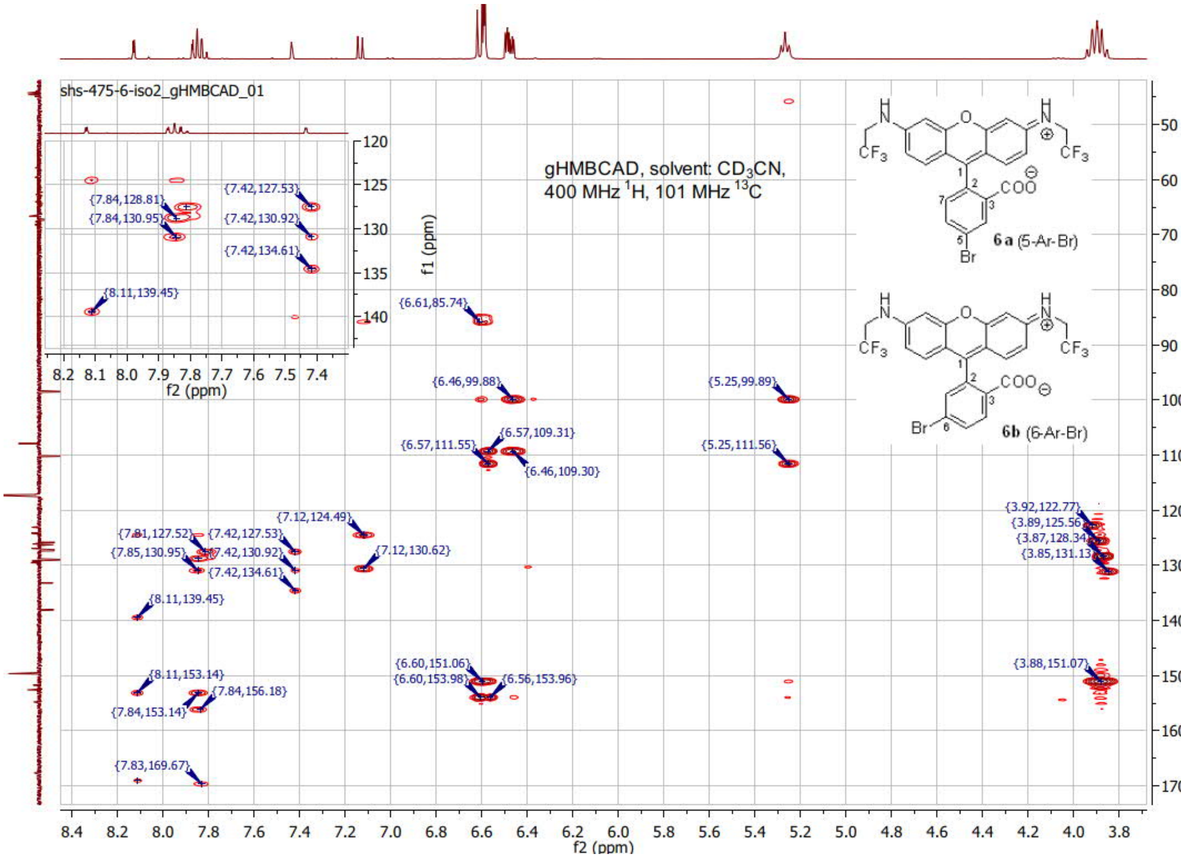

shs-475-6-iso2_gHMBCAD_01
gHMBCAD, solvent: CD3CN,
400 MHz 1H, 101 MHz 13C
{7.84,128.81}
{7.84,130.95}
{7.42,127.53}
{7.42,130.92}
{7.42,134.61}
{8.11,139.45}
{6.61,85.74}
{6.46,99.88}
{5.25,99.89}
{6.57,111.55}
{6.57,109.31}
{6.46,109.30}
{5.25,111.56}
{7.12,124.49}
{7.81,127.52}
{7.85,130.95}
{7.12,130.62}
{3.92,122.77}
{3.89,125.56}
{3.87,128.34}
{3.85,131.13}
{8.11,153.14}
{7.84,156.18}
{7.84,153.14}
{6.60,151.06}
{6.60,153.98}
{6.56,153.96}
{3.88,151.07}
{7.83,169.67}
6a (5-Ar-Br)
6b (6-Ar-Br)
f1 (ppm)
f2 (ppm)

^{19}F NMR (376 MHz, DMSO-d_6) δ -70.53 (t, J = 9.6 Hz, 12F) ppm.

shs475-1-iso-2a_FLUORINE_01

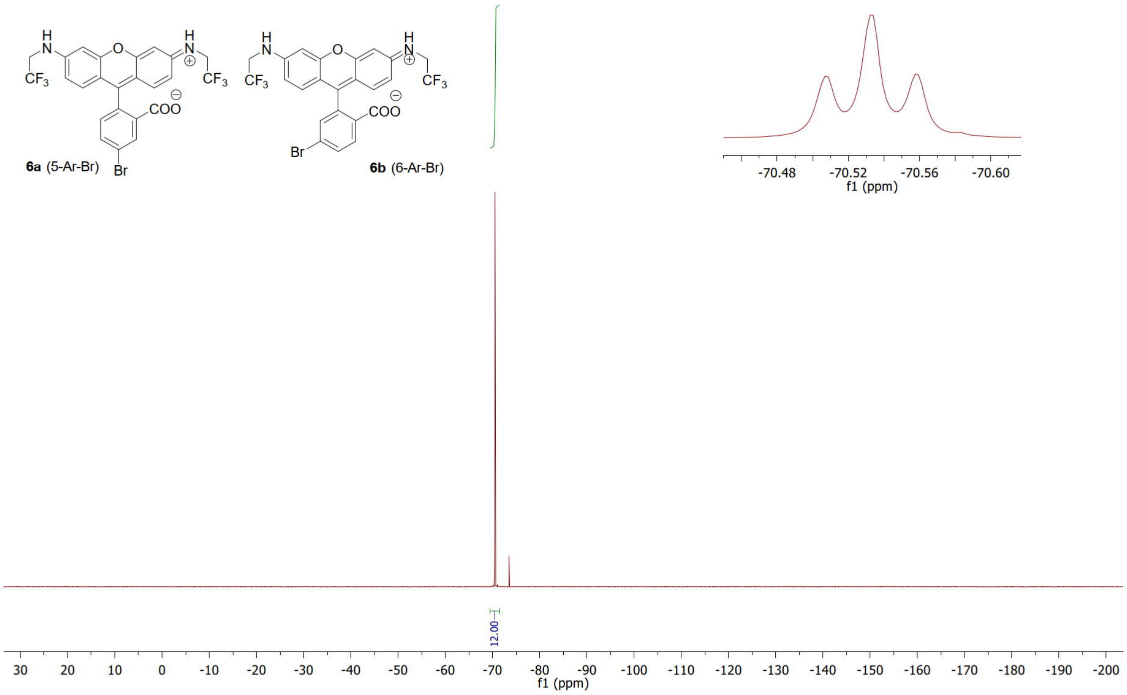

Bis(rhodamine) acetylenes **7a** (peak 3), **7b** (peak 2), and **7c** (peak 3) are 5,5'-, 5,6′- and 6,6′-isomers, respectively.

**Probe :**	**shs477-31-iso3**						
**Lösungsmittel :**		**MeCN/H2O**		**Aufgabemenge:**		**3.0 µl**	
**Säule:**	Phenomenex	Kinetex C18	2.6 µm	**Länge:**	75 mm	**iO :**	3.0 mm
**Fluß (ml / Min) :**		**0.5**	**Temperatur :**		**25.0**		
**Detektor:**	DAD-3000		**Pumpe:**	HPG-3200SD		**Sampler:**	WPS-3000
**Laufmittel:**		**A = Acetonitril 0.1% FA**				**B = Wasser 0.1% FA**	
**Gradient:**	A 20.0 %	B 80.0 %	**---->**	A 100.0 %	B 0.0 %	T = 10 Min.	

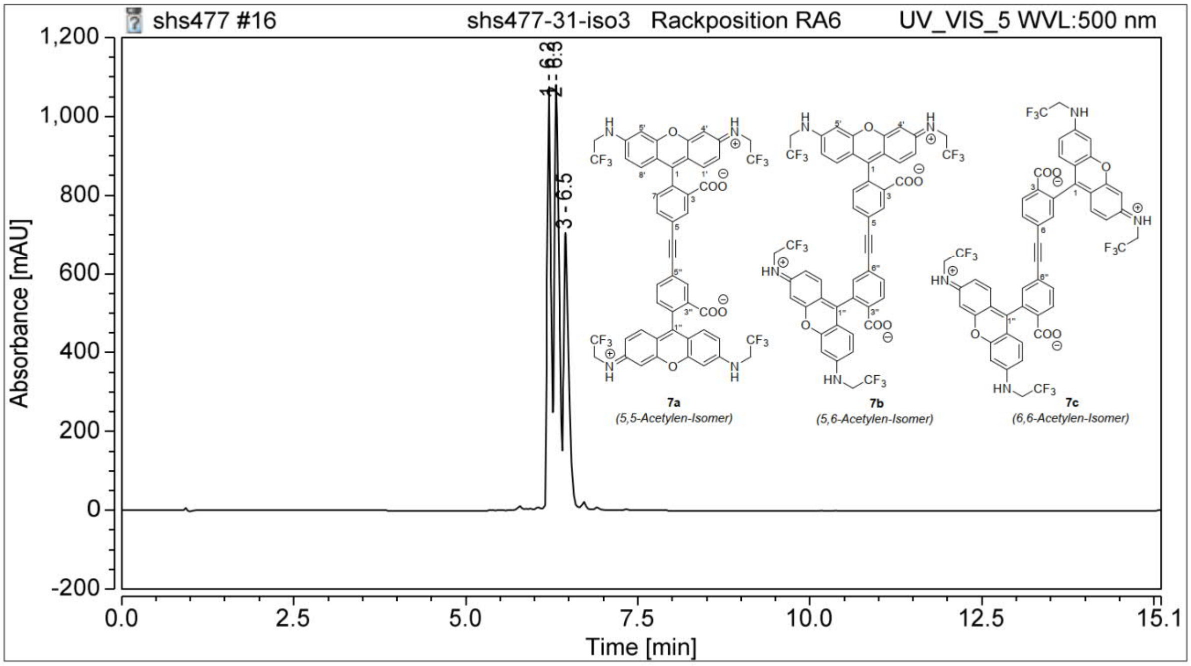

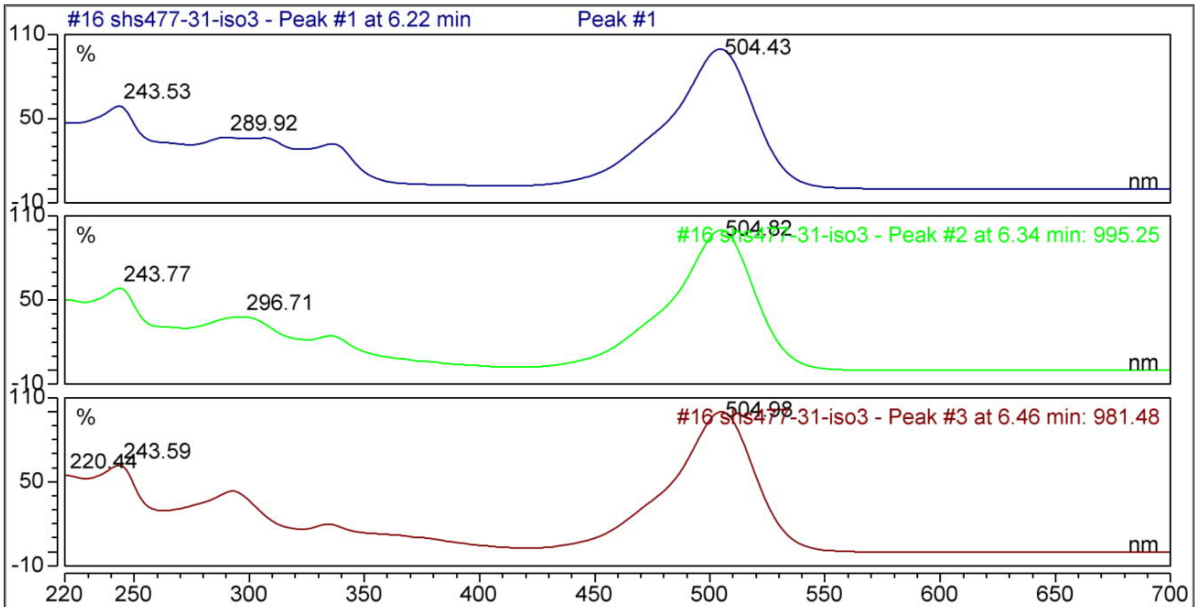

Analytical HPLC traces and NMR spectra of the individual regioisomers **7a** (peak 3), **7b** (peak 2) and **7c** (peak 1) isolated by means of preparative HPLC

Compound **7a**

**Probe :**	**shs477-31 iso3 prep Peak 3 RT=11,0**					
**Lösungsmittel :**	**MeCN/H2O**		**Aufgabemenge:**		**3.0 µl**	
**Säule:**	Phenomenex	2.6 µm	**Länge:**	75 mm	**iO :**	3.0 mm
**Fluß (ml / Min) :**	**0.5**	**Temperatur :**		**25.0**		
**Detektor:**	DAD-3000	**Pumpe:**	HPG-3200SD		**Sampler:**	WPS-3000
**Laufmittel:**	**A = Acetonitril 0.1% FA**				**B = Wasser 0.1% FA**	
**Gradient:**	A 20.0 %	B 80.0 %	---->	A 100.0 %	B 0.0 %	T = 10 Min.

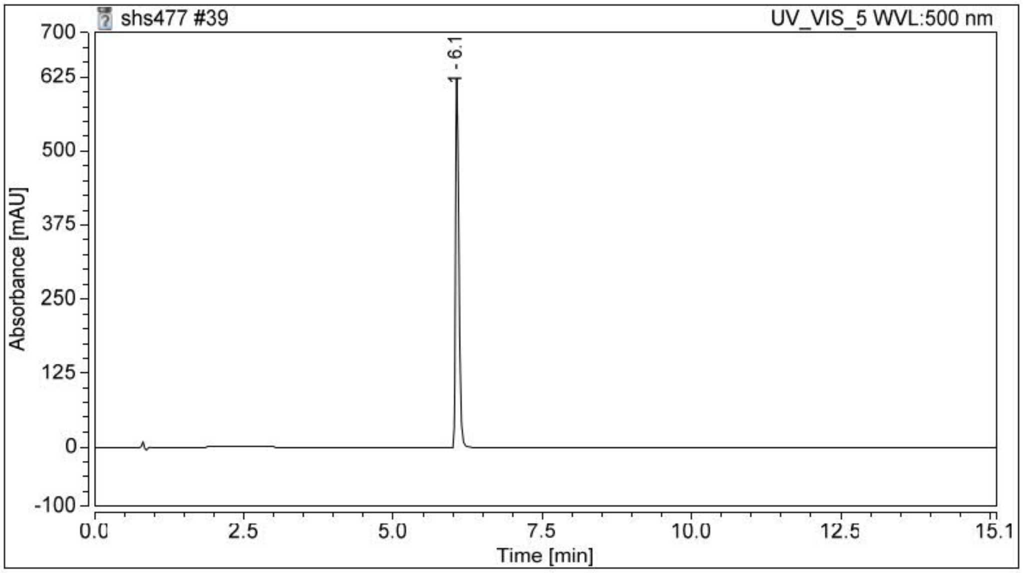

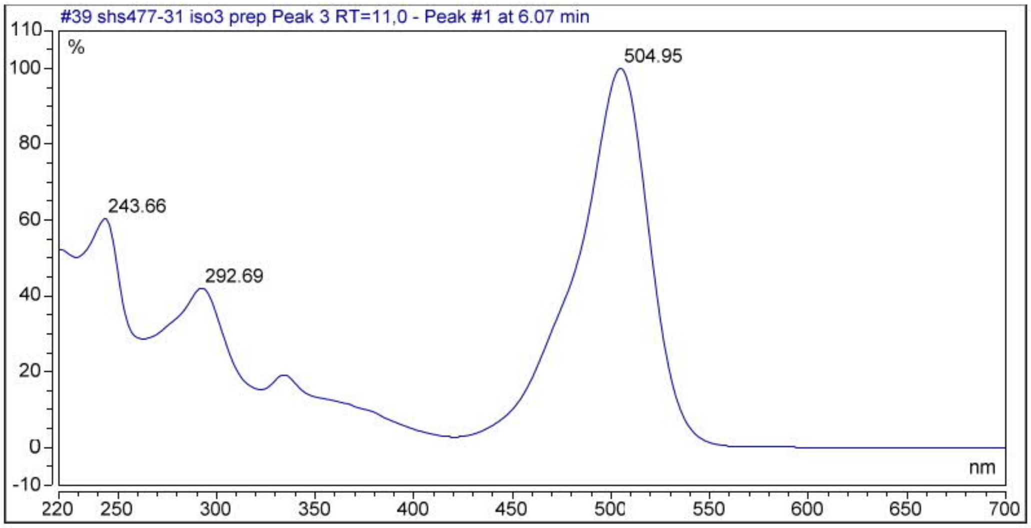

^{1}H NMR (400 MHz, Acetonitrile-d_3) δ 8.16 (dd, J = 1.5, 0.8 Hz, 1H), 7.90 (dd, J = 8.0, 1.5 Hz, 2H), 7.26 (dd, J = 8.0, 0.9 Hz, 2H), 6.63 (d, J = 8.7 Hz, 3H), 6.59 (d, J = 2.4 Hz, 3H), 6.48 (dd, J = 8.7, 2.4 Hz, 4H), 5.28 (t, J = 7.0 Hz, 3H), 3.89 (qd, J = 9.3, 6.8 Hz, 8H).

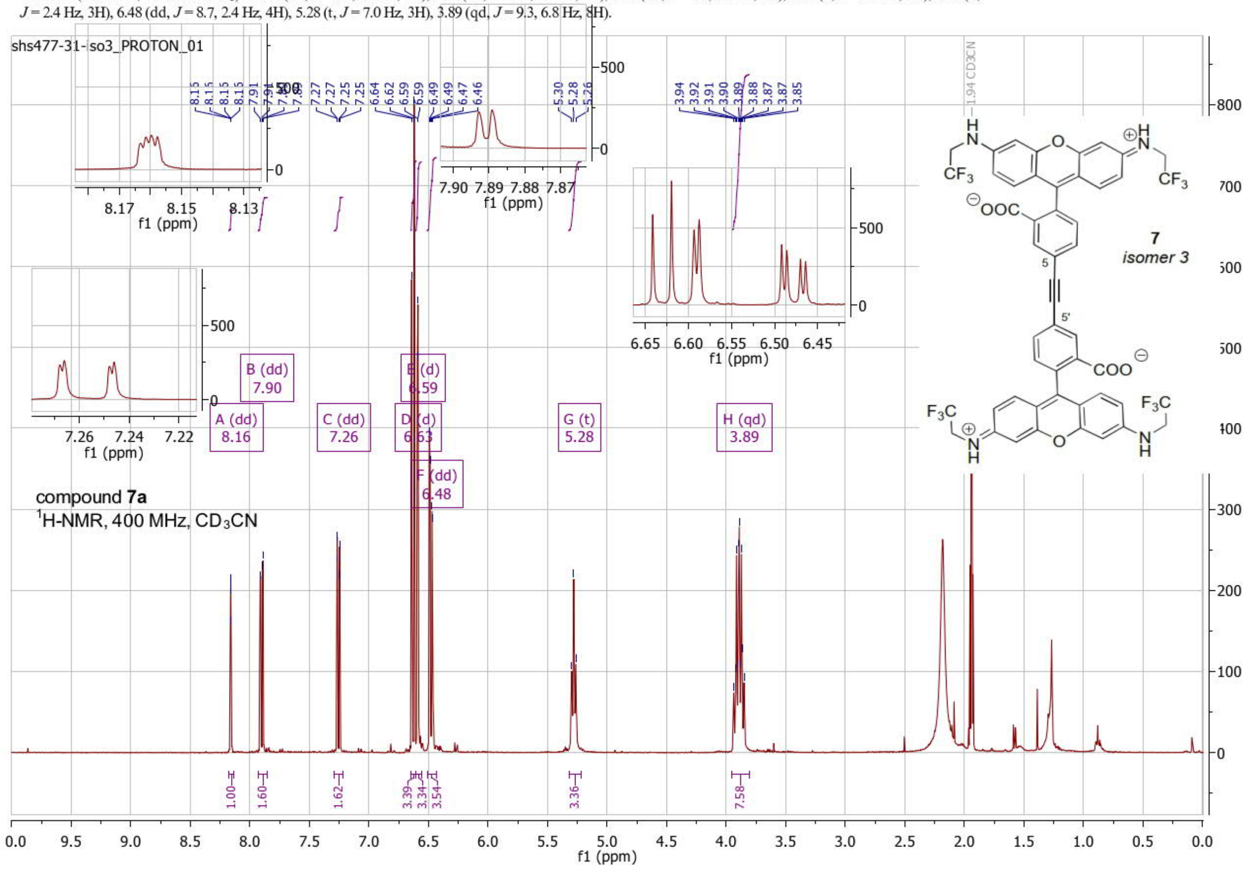

^{13}C NMR (101 MHz, Acetonitrile-d_3) δ 45.81 (q, J = 33.4 Hz).

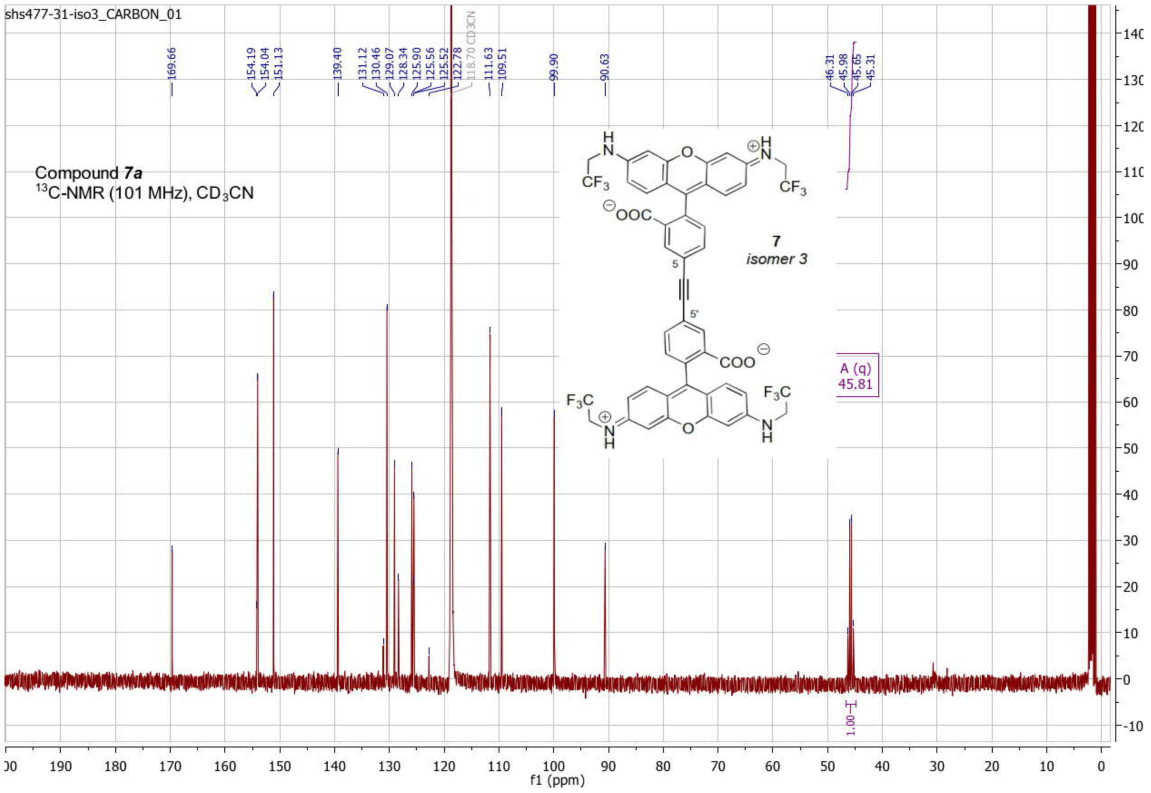

Compound **7b**

Probe :	shs477-31 iso4 prep Peak 2 RT=10,5 nach				
Lösungsmittel :	MeCN/H2O	Aufgabemenge:	3.0 µl		
Säule:	Phenomenex 2.6 µm	Länge:	75 mm	iO :	3.0 mm
Fluß (ml / Min) :	0.5	Temperatur :	25.0		
Detektor:	DAD-3000	Pumpe:	HPG-3200SD	Sampler:	WPS-3000
Laufmittel:	A = Acetonitril 0.1% FA			B = Wasser 0.1% FA	
Gradient:	A 20.0 % B 80.0 % ---->	A 100.0 % B 0.0 %	T = 10 Min.		

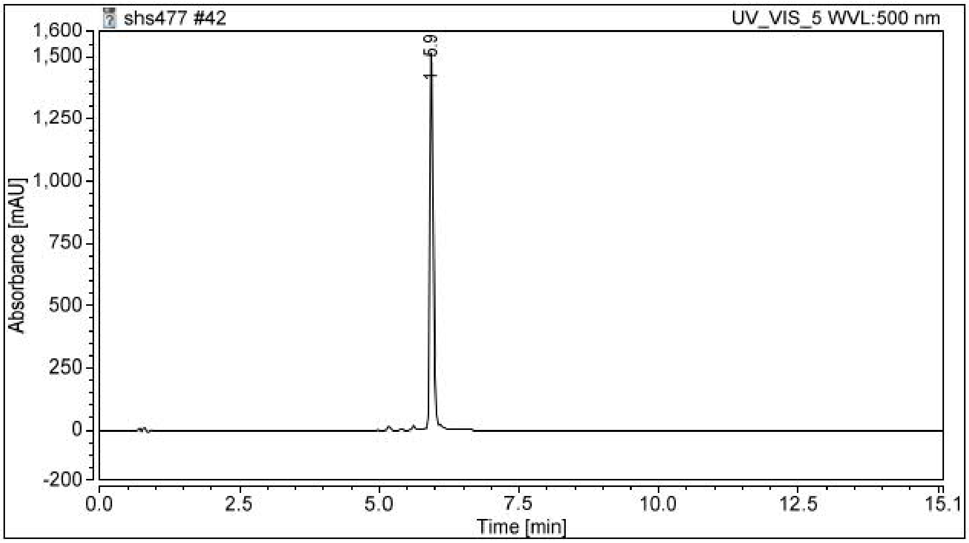

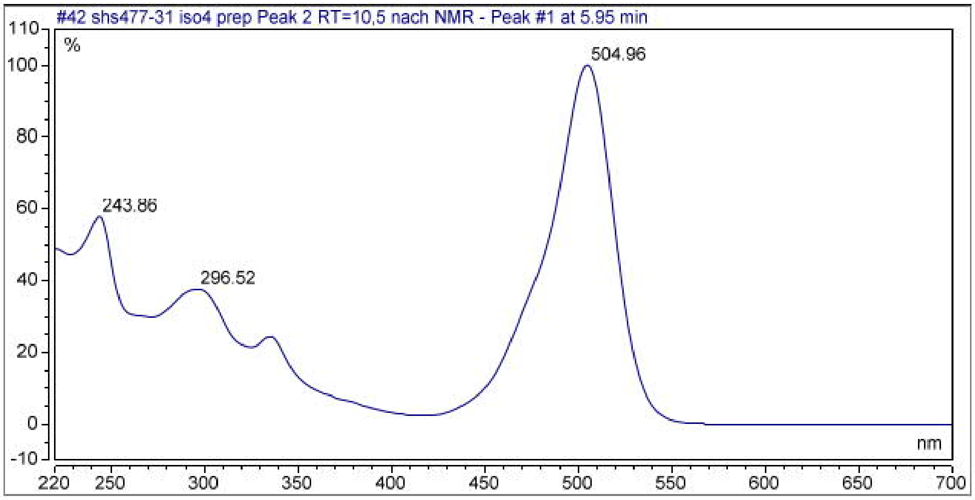

^{1}H NMR (400 MHz, Acetonitrile-d_3) δ 7.46 (dd, J = 1.4, 0.7 Hz, 1H), 7.25 (dd, J = 8.0, 0.7 Hz, 1H), 5.74 (s, 5H), 4.01 – 3.91 (m, 11H).

δ 8.17 (dd, J = 1.6, 0.7 Hz, 1H), 8.09 (dd, J = 8.0, 0.7 Hz, 1H), 7.89 (dd, J = 8.0, 1.4 Hz, 1H), 7.86 (dd, J = 8.0, 1.5 Hz, 1H).

δ 6.81 (d, J = 8.8 Hz, 1H), 6.75 (d, J = 8.8 Hz, 1H), 6.71 (dd, J = 8.4, 2.4 Hz, 2H), 6.62 (dd, J = 8.8, 2.4 Hz, 1H), 6.59 (dd, J = 8.8, 2.4 Hz, 1H).

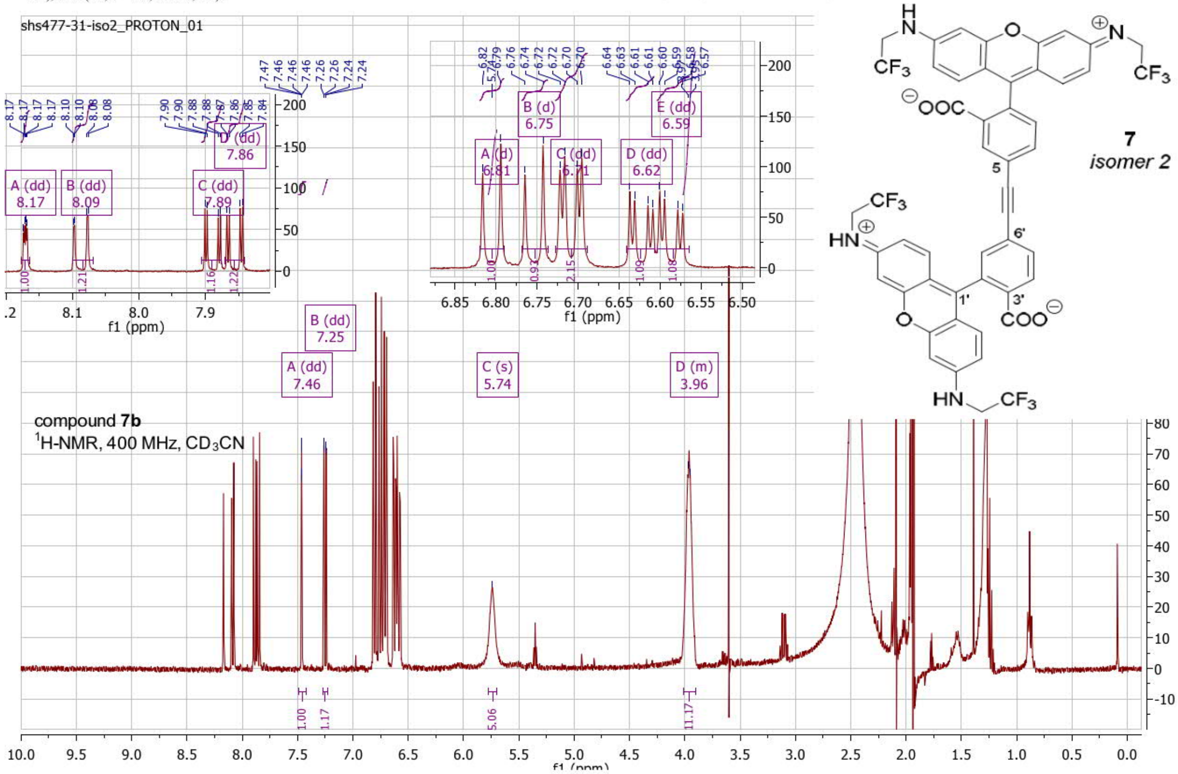

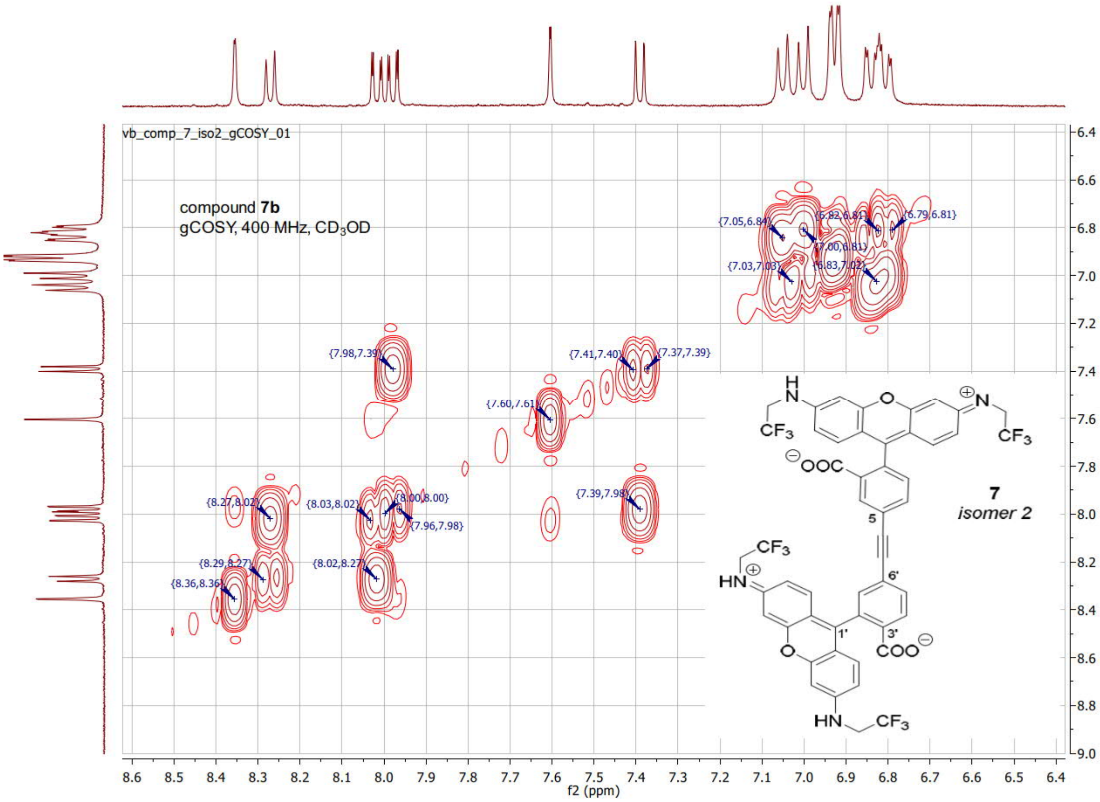

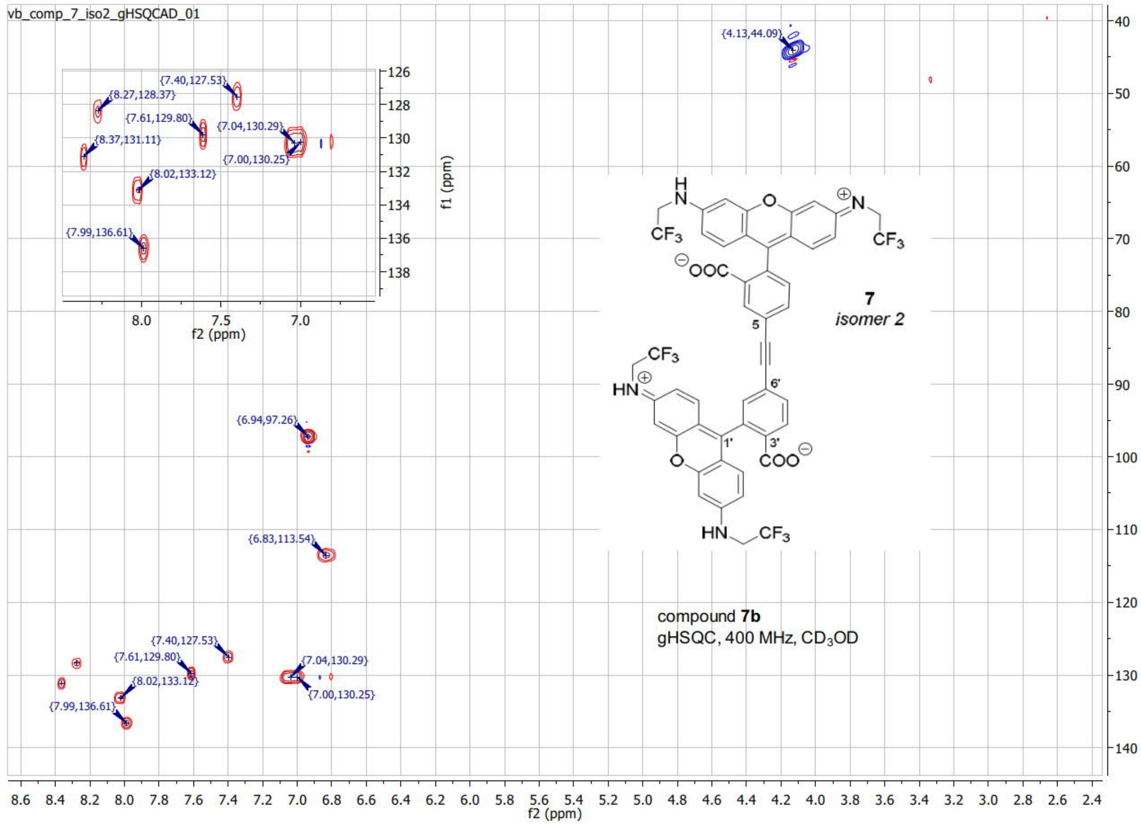
vb_comp_7_iso2_gHSQCAD_01
7
isomer 2
compound 7b
gHSQC, 400 MHz, CD3OD
f2 (ppm)
f1 (ppm)

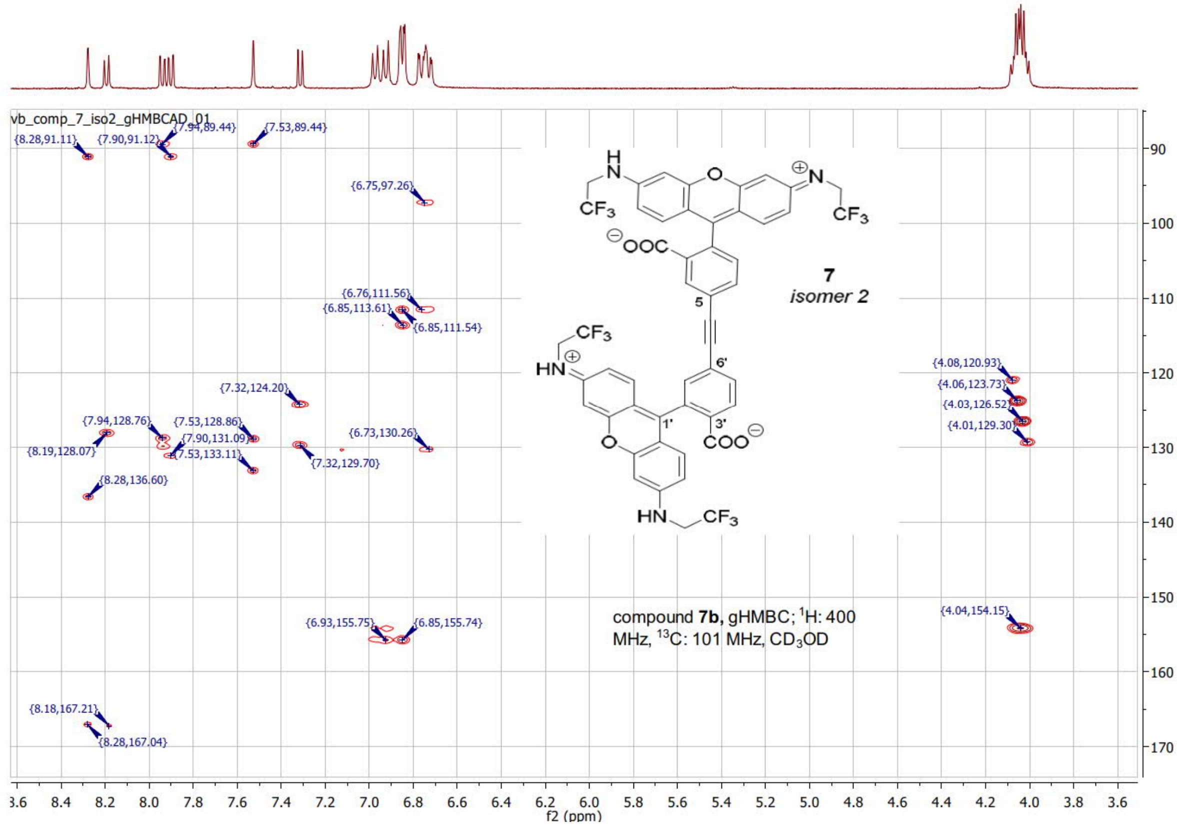
vb_comp_7_iso2_gHMBCAD_01
7
isomer 2
compound 7b, gHMBC; 1H: 400 MHz, 13C: 101 MHz, CD3OD
f2 (ppm)

Compound **7c**

Probe :	shs477-31 iso4 prep Peak 1 RT=9,8 nach N				
Lösungsmittel :	MeCN/H2O	Aufgabemenge:	3.0 µl		
Säule:	Phenomenex 2.6 µm	Länge:	75 mm	iO :	3.0 mm
Fluß (ml / Min) :	0.5	Temperatur :	25.0		
Detektor:	DAD-3000	Pumpe:	HPG-3200SD	Sampler:	WPS-3000
Laufmittel:	A = Acetonitril 0.1% FA			B = Wasser 0.1% FA	
Gradient:	A 20.0 % B 80.0 %	---->	A 100.0 % B 0.0 %	T = 10 Min.	

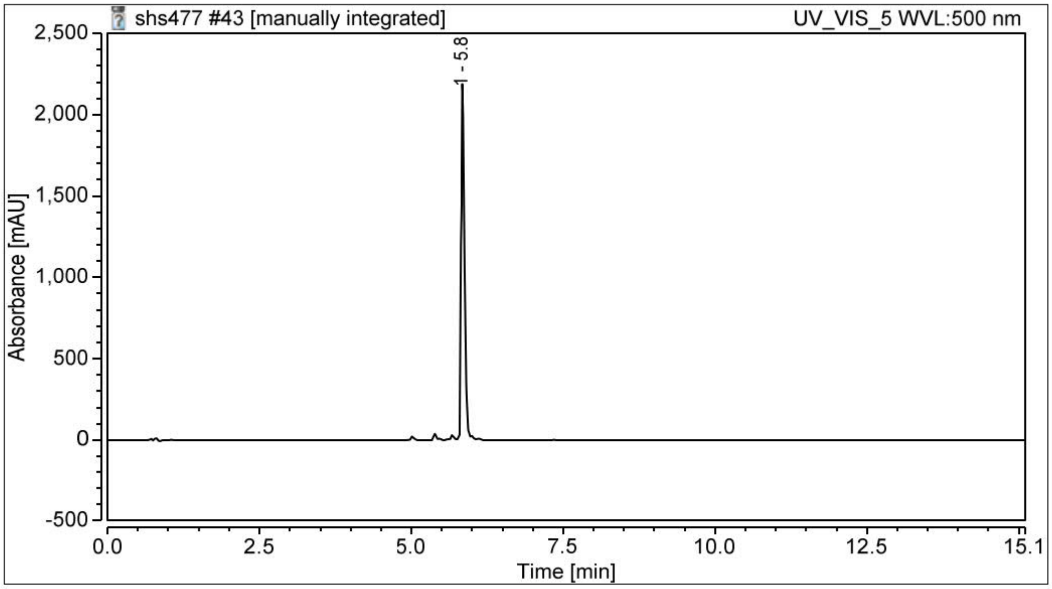

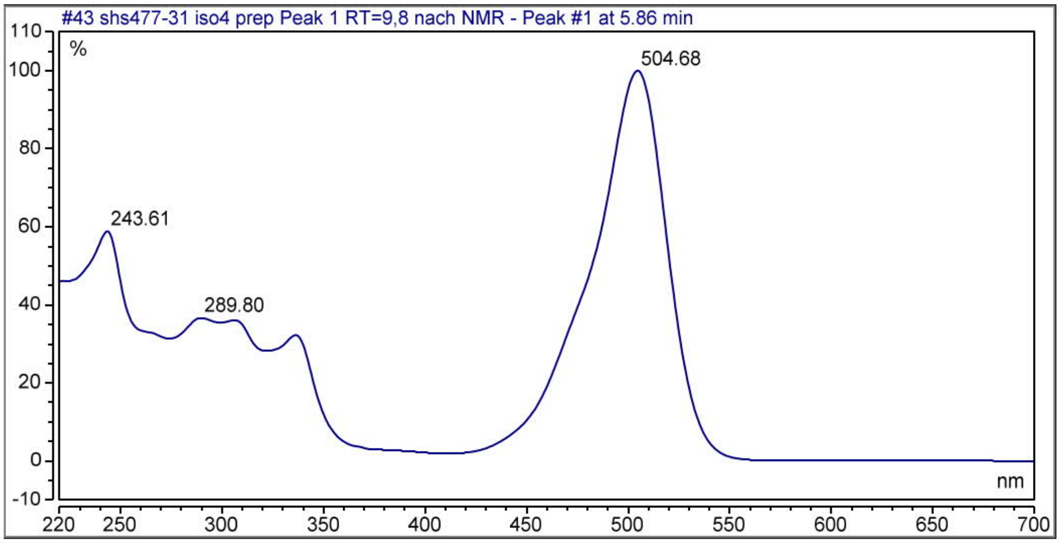

^{1}H NMR (400 MHz, Methanol-d_4) δ 8.32 (d, J = 7.9 Hz, 1H), 8.00 – 7.91 (m, 1H), 7.63 (d, J = 1.4 Hz, 1H), 7.19 (d, J = 9.2 Hz, 2H), 7.13 (d, J = 2.1 Hz, 2H), 7.00 (dd, J = 9.1, 2.2 Hz, 2H), 4.23 (q, J = 9.0 Hz, 4H).

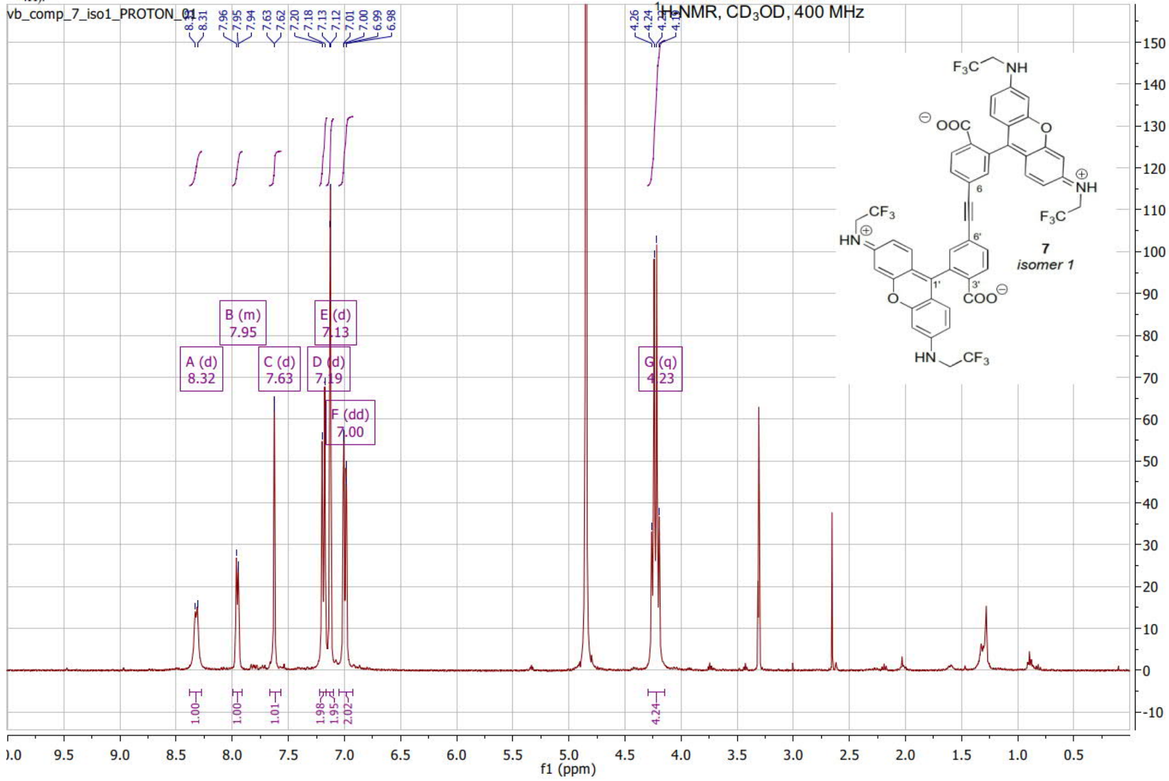

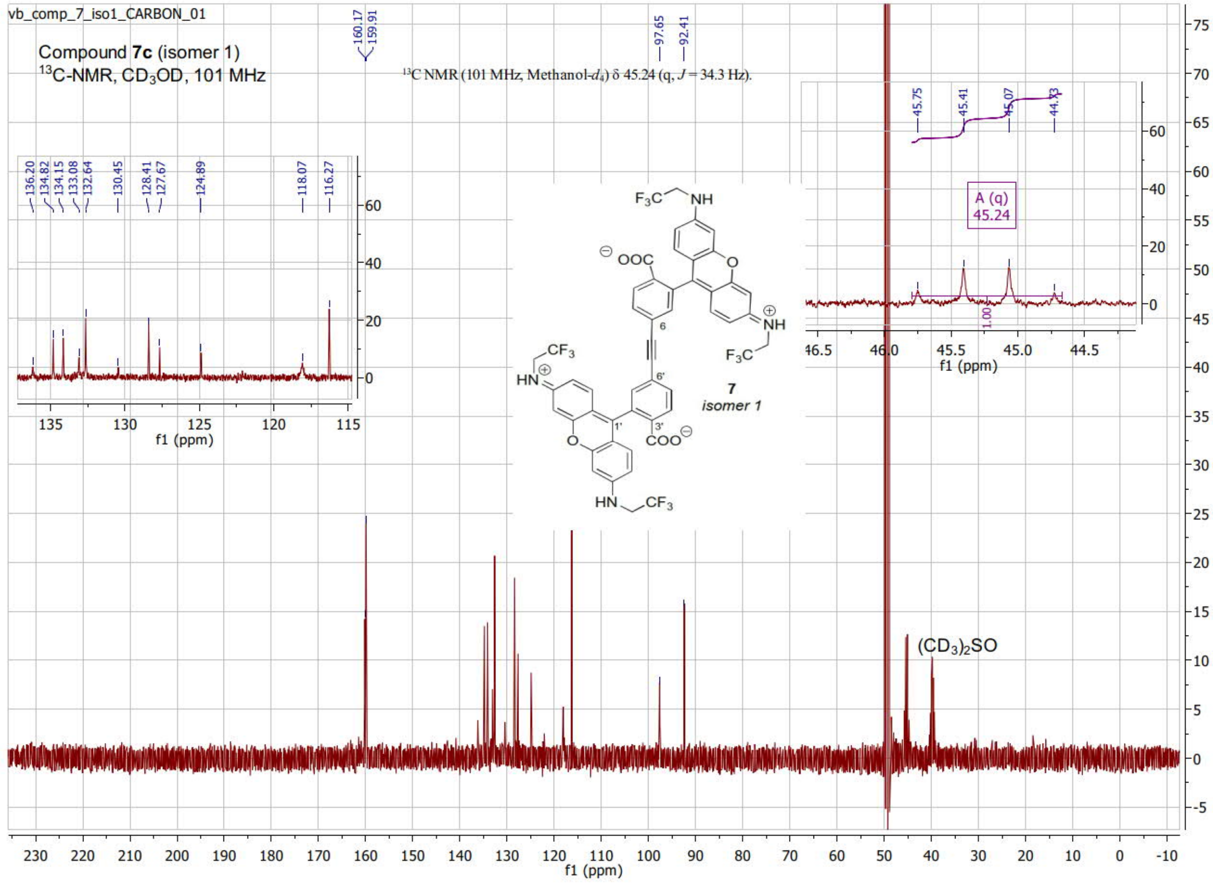

^{13}C NMR (101 MHz, Methanol-d_4) δ 45.24 (q, J = 34.3 Hz).

Bis-rhodamine ethanones **8a** [5(CH_2),5(CO)] (peak 4), **8b** [6(CH_2),5(CO)] (peak 3), **8c** [5(CH_2),6(CO)] (peak 2) and **8d** [6(CH_2),6(CO)] (peak 1)

HPLC trace of the mixture of regioisomers

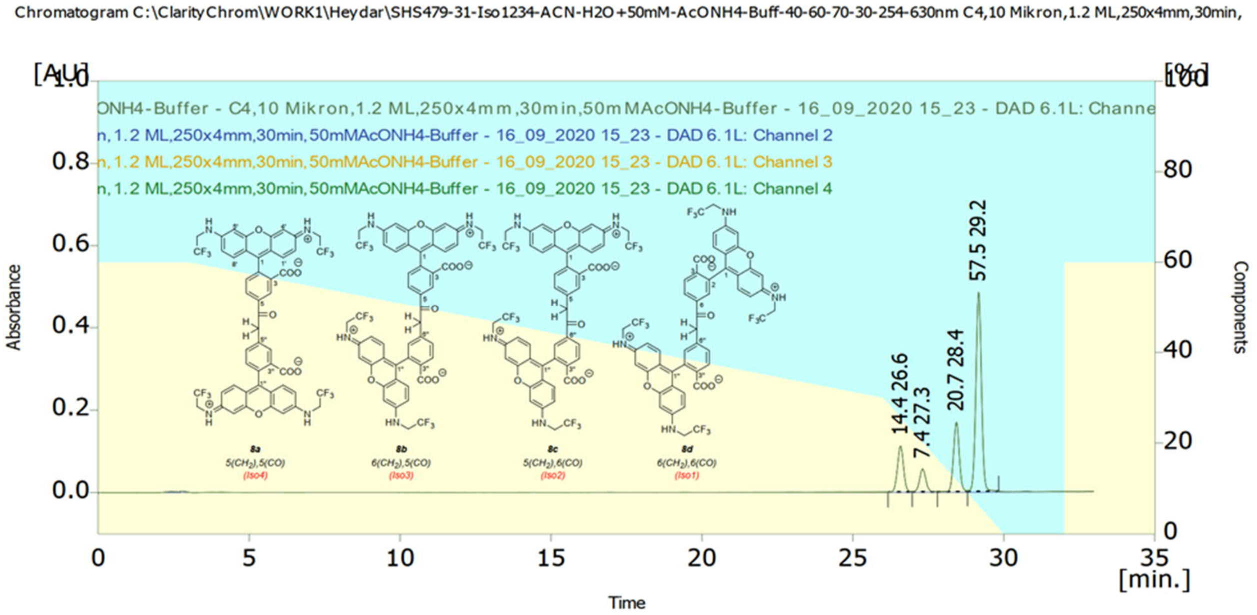

HPLC traces and NMR spectra of the isolated individual regioisomers **8a-d**

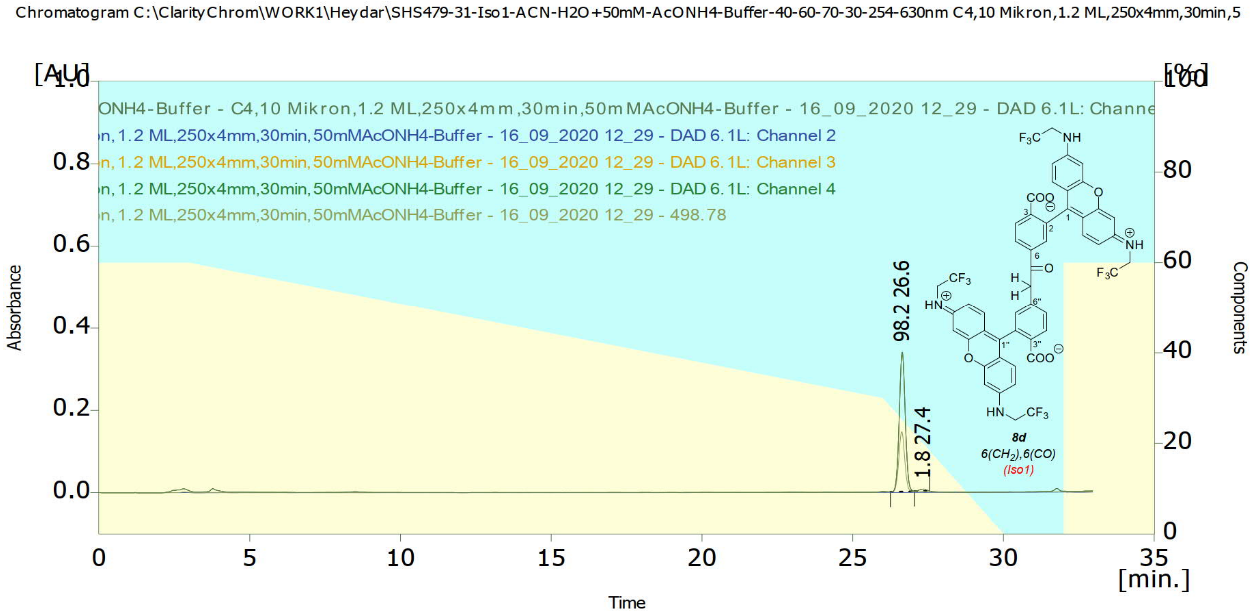

Chromatogram C:\ClarityChrom\WORK1\Heydar\SHS479-31-Iso2-ACN-H2O+50mM-AcONH4-Buffer-40-60-70-30-254-630nm C4,10 Mikron,1.2 ML,250x4mm,30min,5

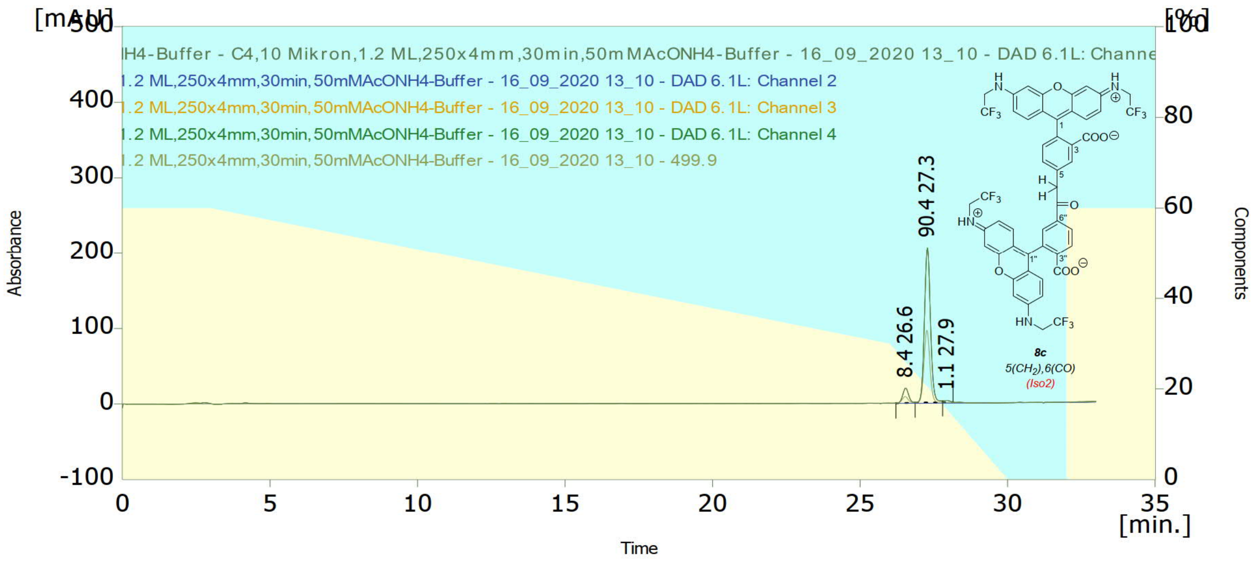

Chromatogram C:\ClarityChrom\WORK1\Heydar\SHS479-31-Iso3-ACN-H2O+50mM-AcONH4-Buffer-40-60-70-30-254-630nm C4,10 Mikron,1.2 ML,250x4mm,30min,5

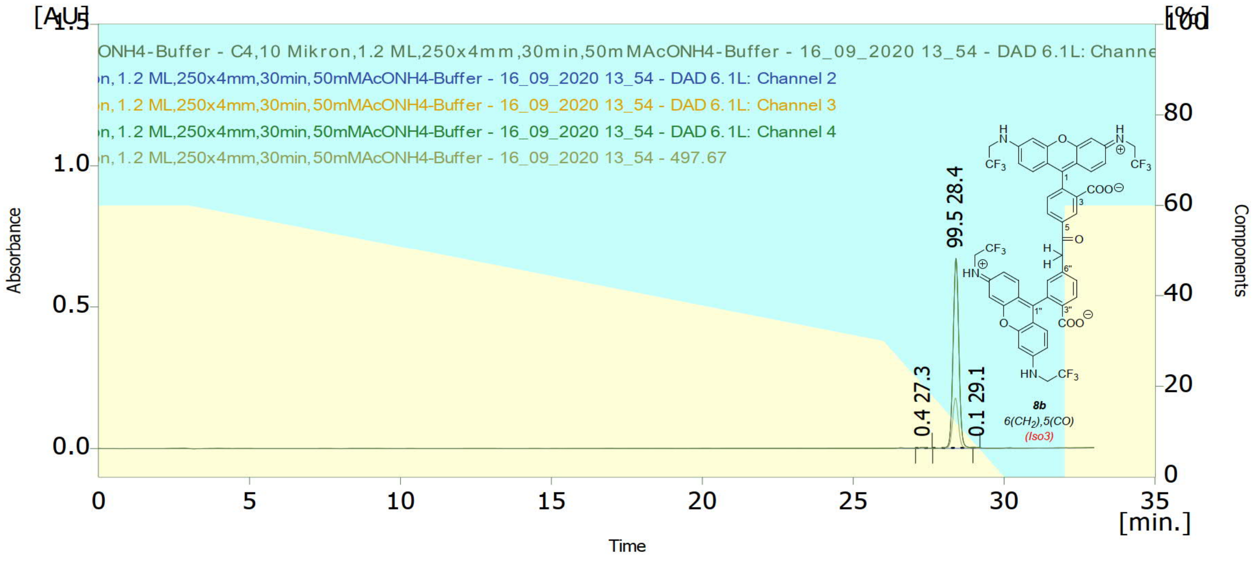

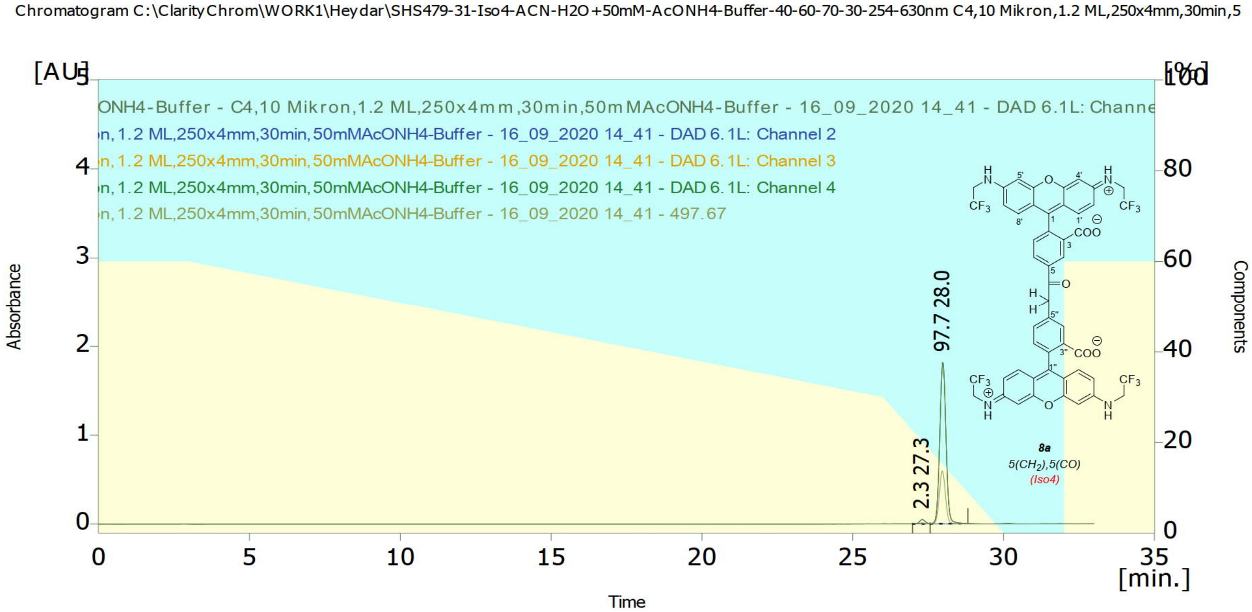

NMR spectra of compound **8a** [5(CH_2)-6(CO)]: ^{1}H (400 MHz) and ^{13}C{^{1}H} (101 MHz)

^{1}H NMR (400 MHz, Acetonitrile-d_3) δ 8.60 (dd, J = 1.6, 0.7 Hz, 1H), 8.37 (dd, J = 8.1, 1.6 Hz, 1H), 7.87 – 7.84 (m, 1H), 7.64 (dd, J = 7.9, 1.6 Hz, 1H), 7.36 (dd, J = 8.0, 0.7 Hz, 1H), 7.18 (dd, J = 7.9, 0.7 Hz, 1H), 6.63 – 6.55 (m, 8H), 6.47 (ddd, J = 8.7, 3.2, 2.4 Hz, 4H), 5.24 (dt, J = 12.1, 7.0 Hz, 4H), 3.89 (qdd, J = 9.4, 7.0, 2.2 Hz, 8H), 4.68 (s, 2H).

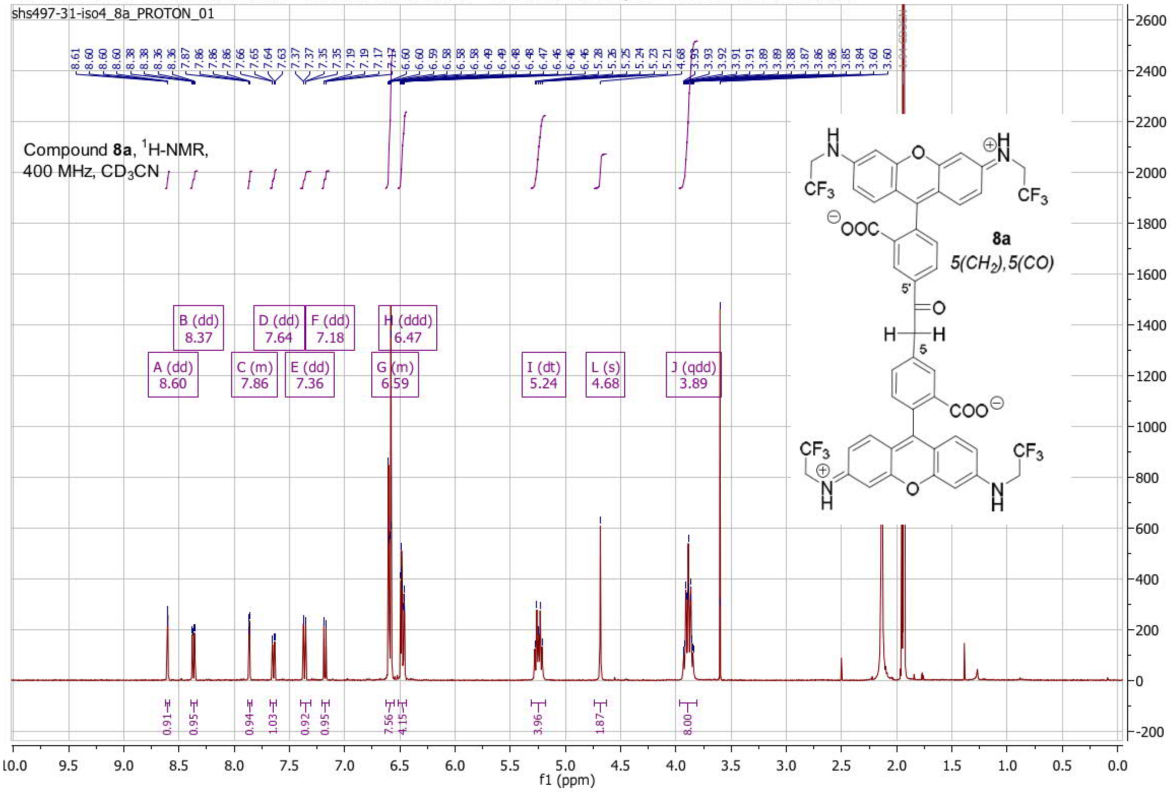

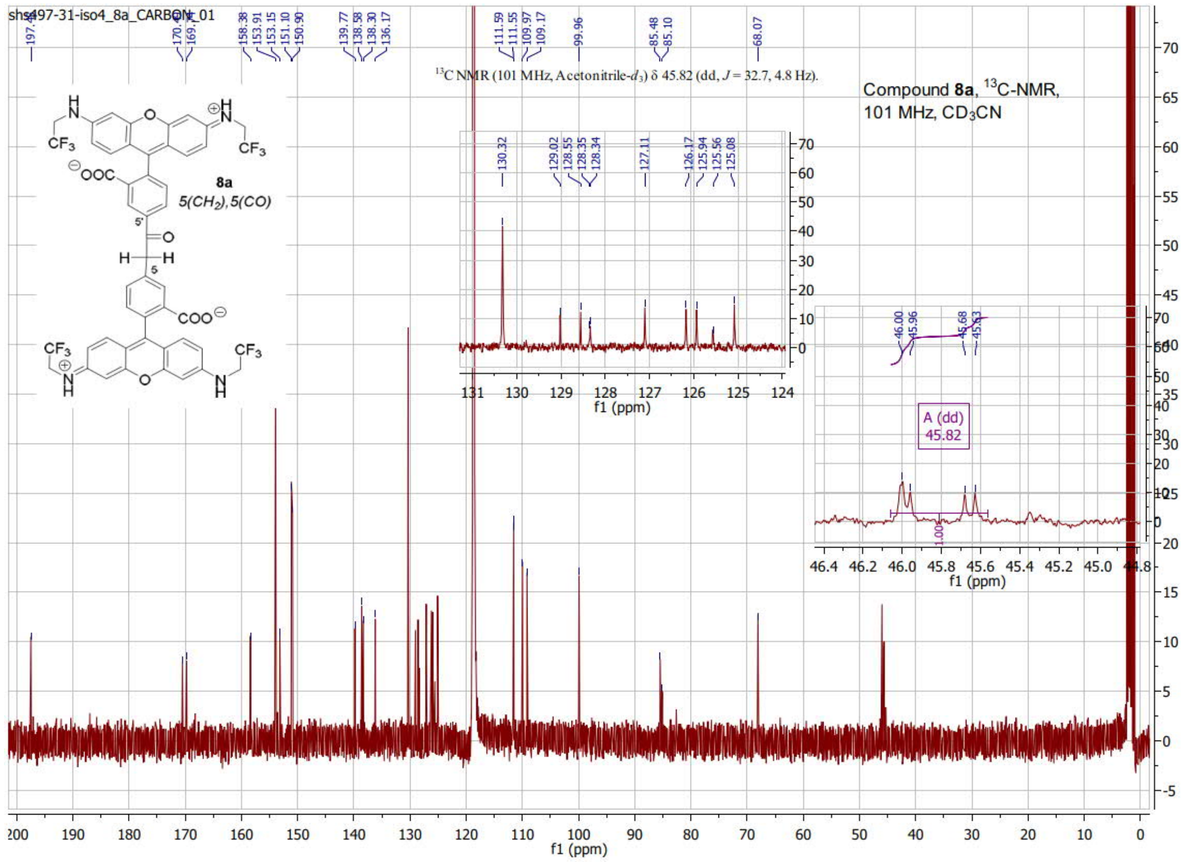

NMR spectra of compound **8b** [6(CH_2)-5(CO)]: ^{1}H (400 MHz) and ^{13}C{^{1}H} (101 MHz)

^{1}H NMR (400 MHz, CD_3CN) δ 8.48 (dd, J = 1.6, 0.8 Hz, 1H), 8.24 (dd, J = 8.1, 1.6 Hz, 1H), 7.93 (dd, J = 7.9, 0.7 Hz, 1H), 7.56 (dd, J = 7.9, 1.4 Hz, 1H), 7.28 (dd, J = 8.1, 0.7 Hz, 1H), 7.11 (dd, J = 1.4, 0.7 Hz, 1H), 6.61 (d, J = 8.7 Hz, 2H), 6.58 – 6.57 (m, 3H), 6.56 (d, J = 7.6 Hz, 2H), 6.52 (s, 1H), 6.45 (ddd, J = 8.9, 6.7, 2.4 Hz, 4H), 5.23 (dt, J = 11.7, 7.0 Hz, 4H), 3.95 – 3.79 (m, 8H) 4.56 (s, 2H).

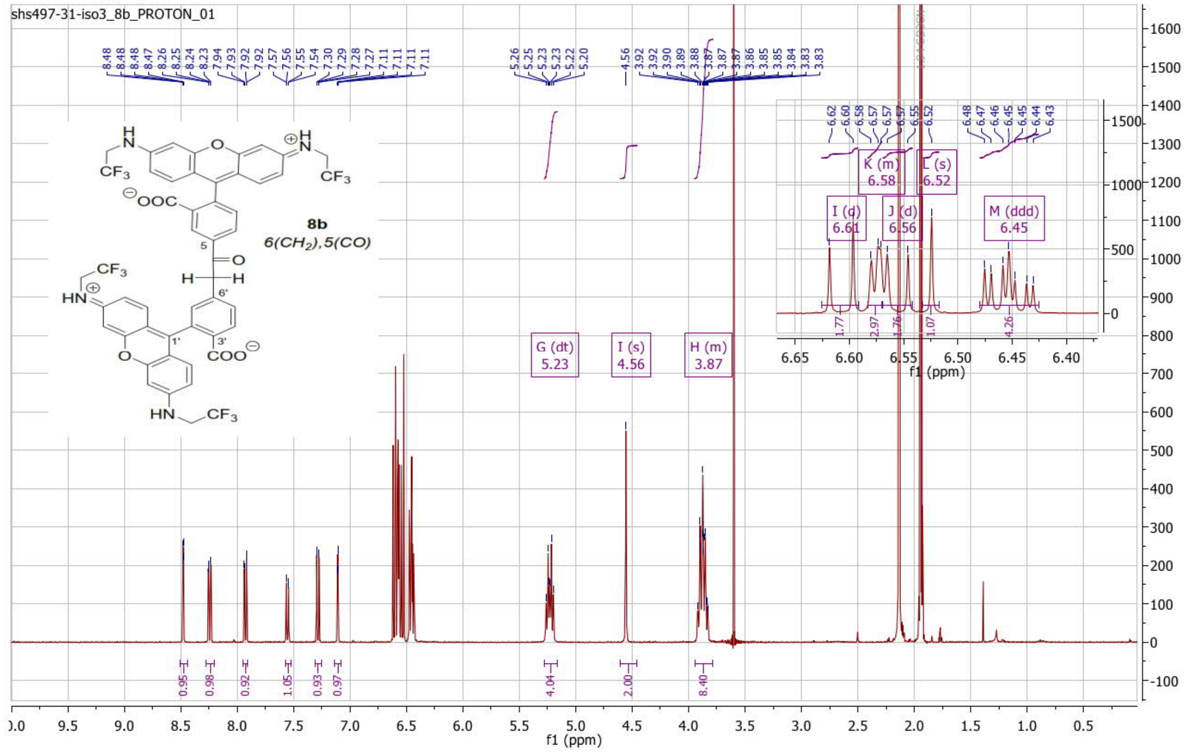

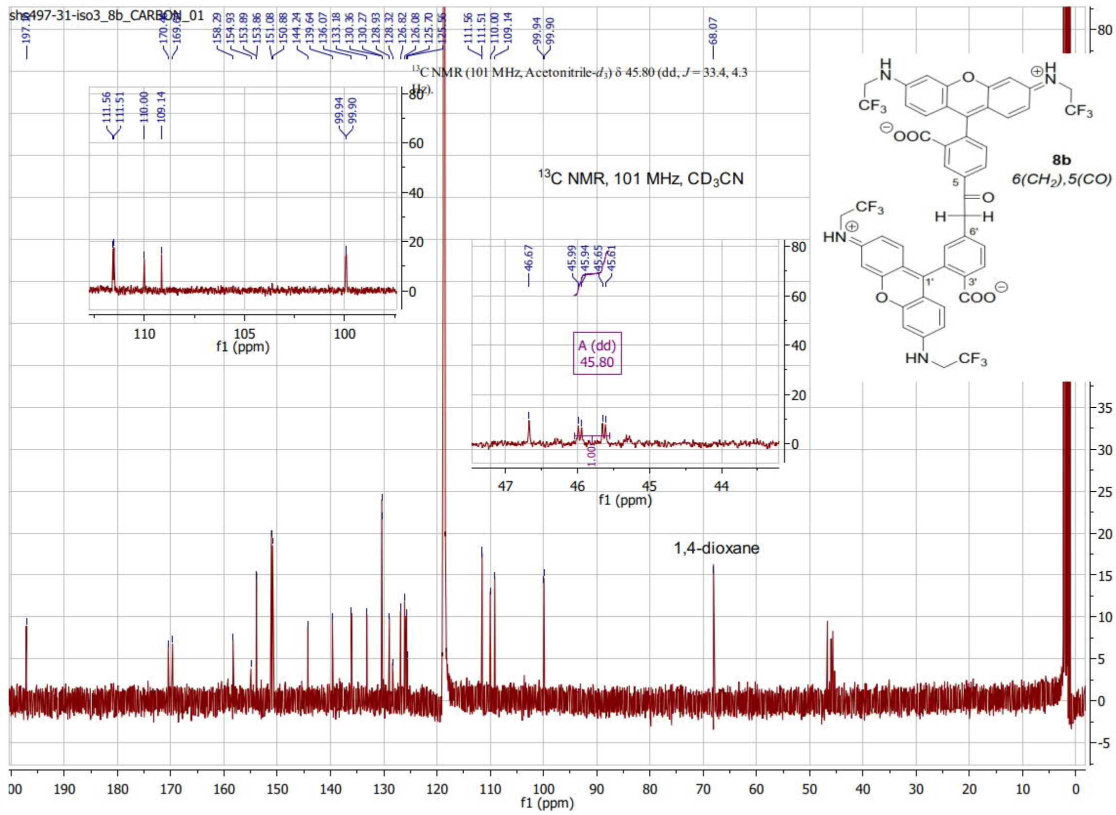

NMR spectra of compound **8c** [5(CH$_2$)-6(CO)]: ^{1}H-, gCOSY, ^{13}C{^{1}H}-, gHSQC and gHMBC.

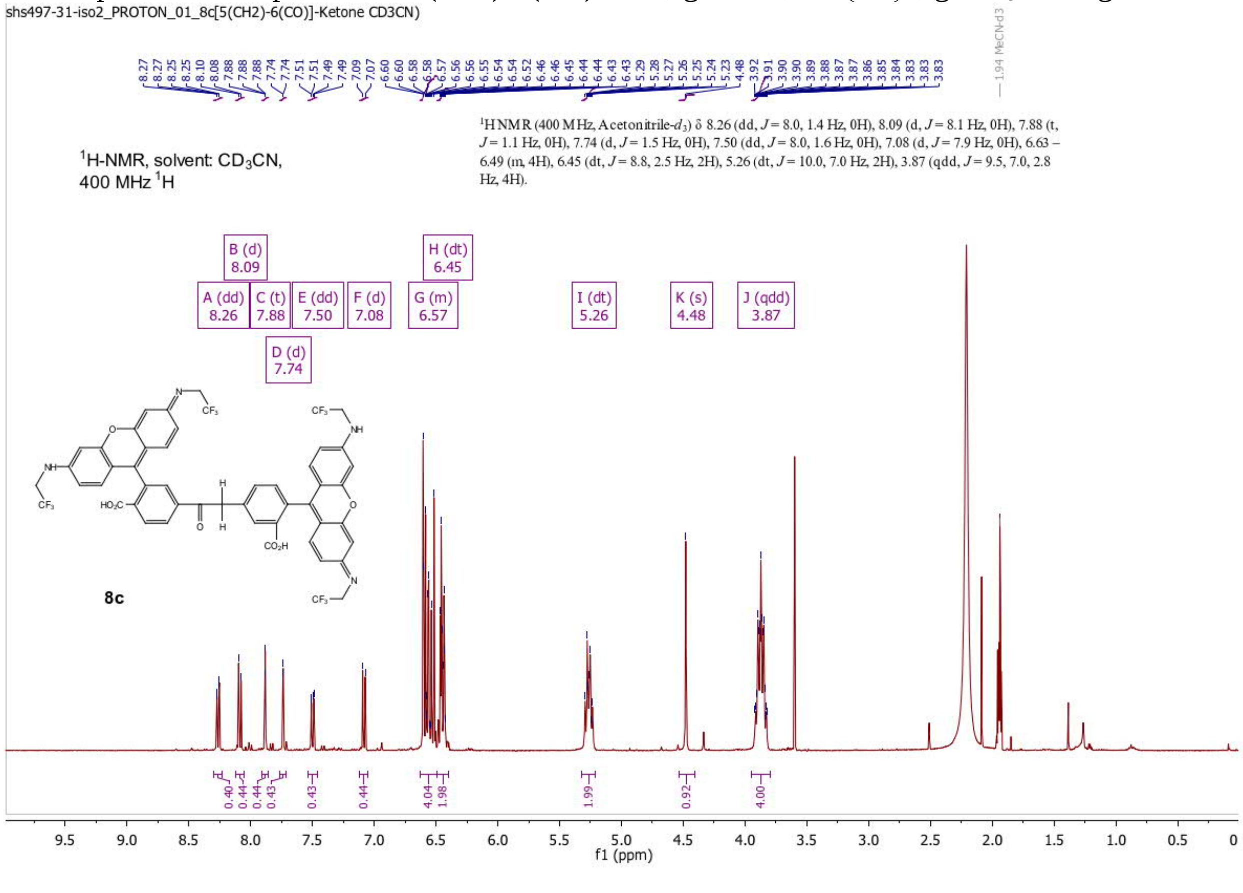

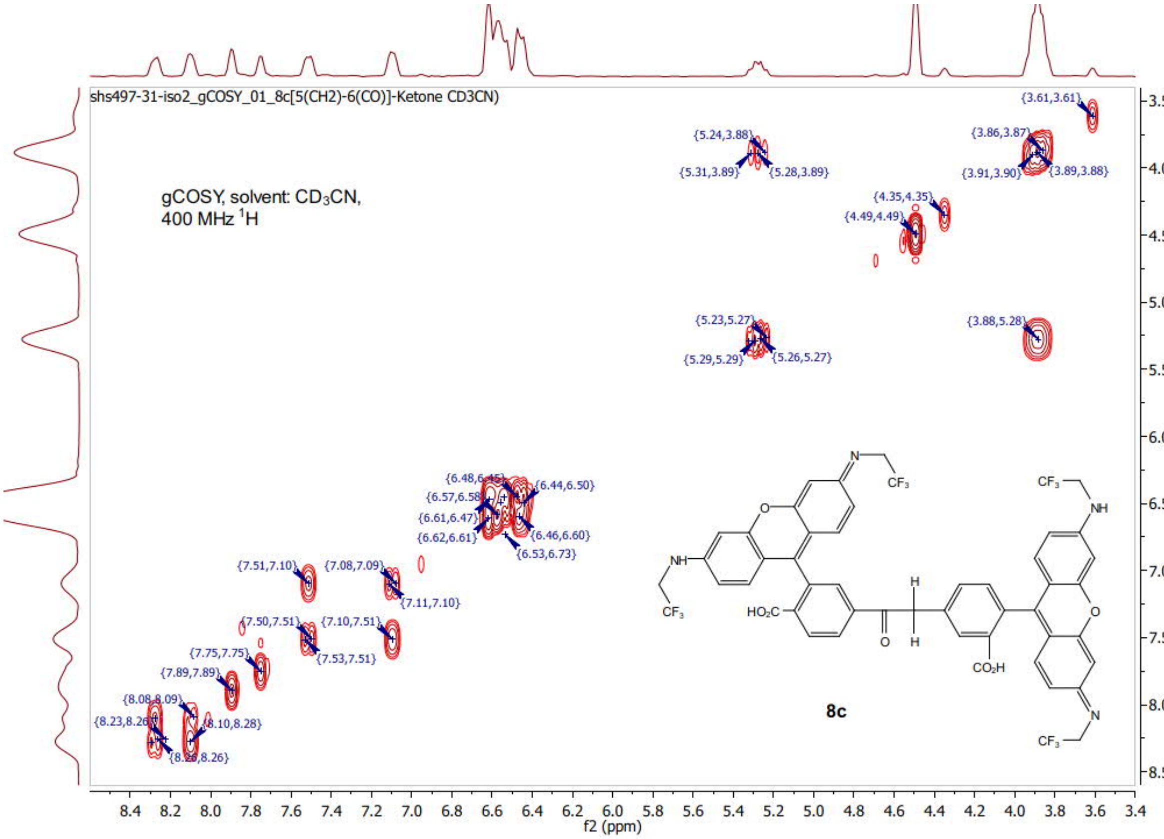
shs497-31-iso2_gCOSY_01_8c[5(CH2)-6(CO)]-Ketone CD3CN)
gCOSY, solvent: CD_3CN,
400 MHz ^1H
8c
f2 (ppm)

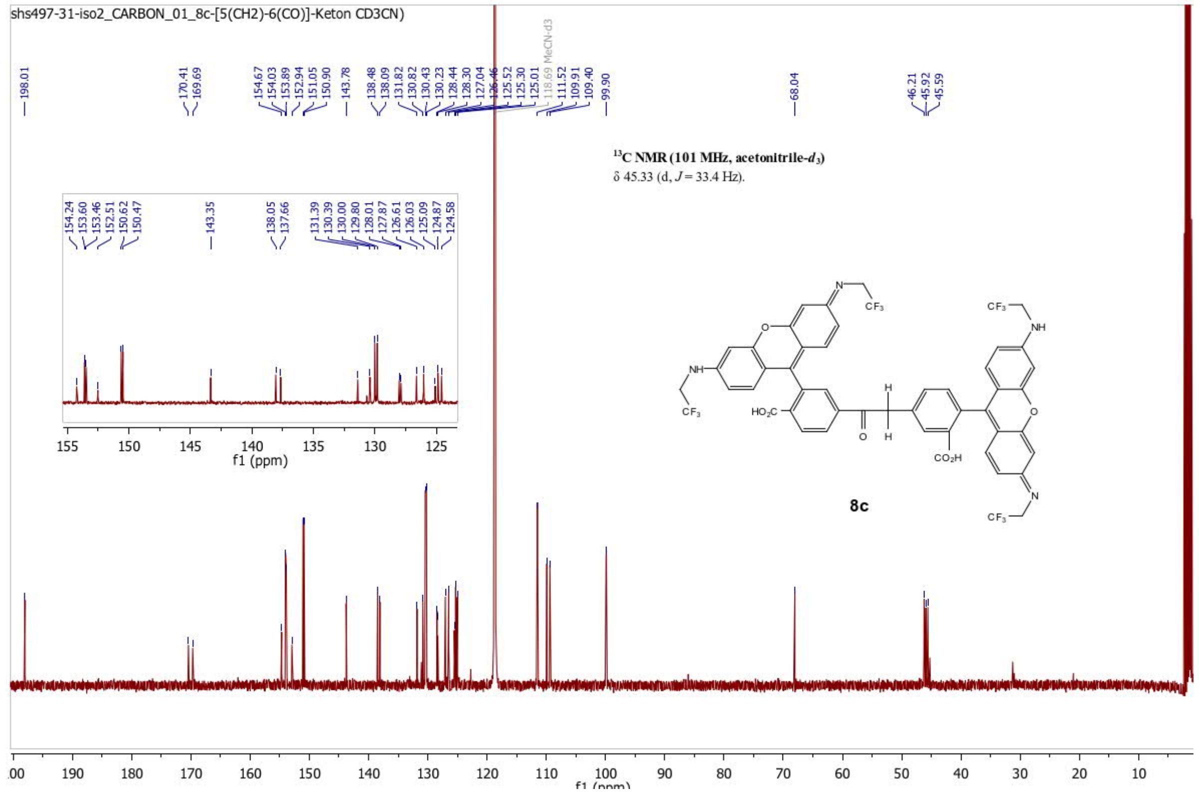
shs497-31-iso2_CARBON_01_8c-[5(CH2)-6(CO)]-Keton CD3CN)
^{13}C NMR (101 MHz, acetonitrile-d_3)
δ 45.33 (d, J = 33.4 Hz).
8c
f1 (ppm)

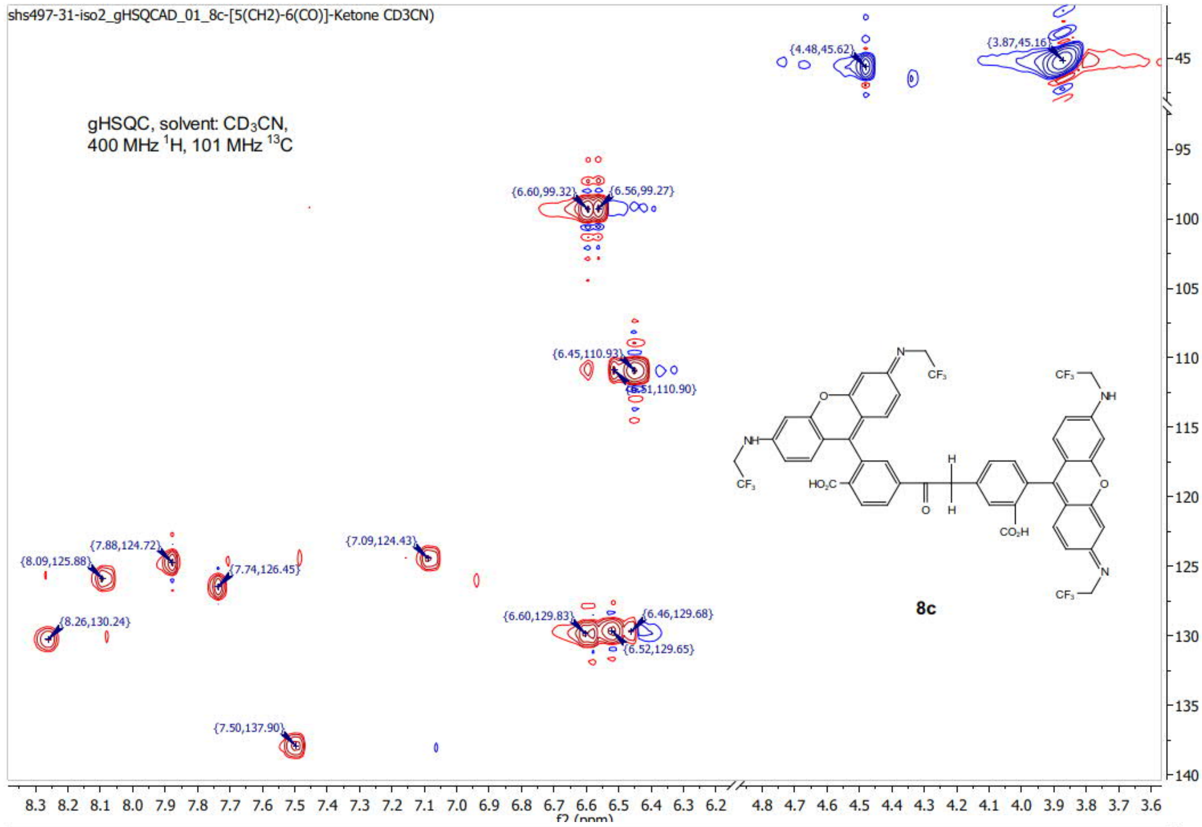
shs497-31-iso2_gHSQCAD_01_8c-[5(CH2)-6(CO)]-Ketone CD3CN)
gHSQC, solvent: CD3CN,
400 MHz 1H, 101 MHz 13C
8c
f2 (ppm)

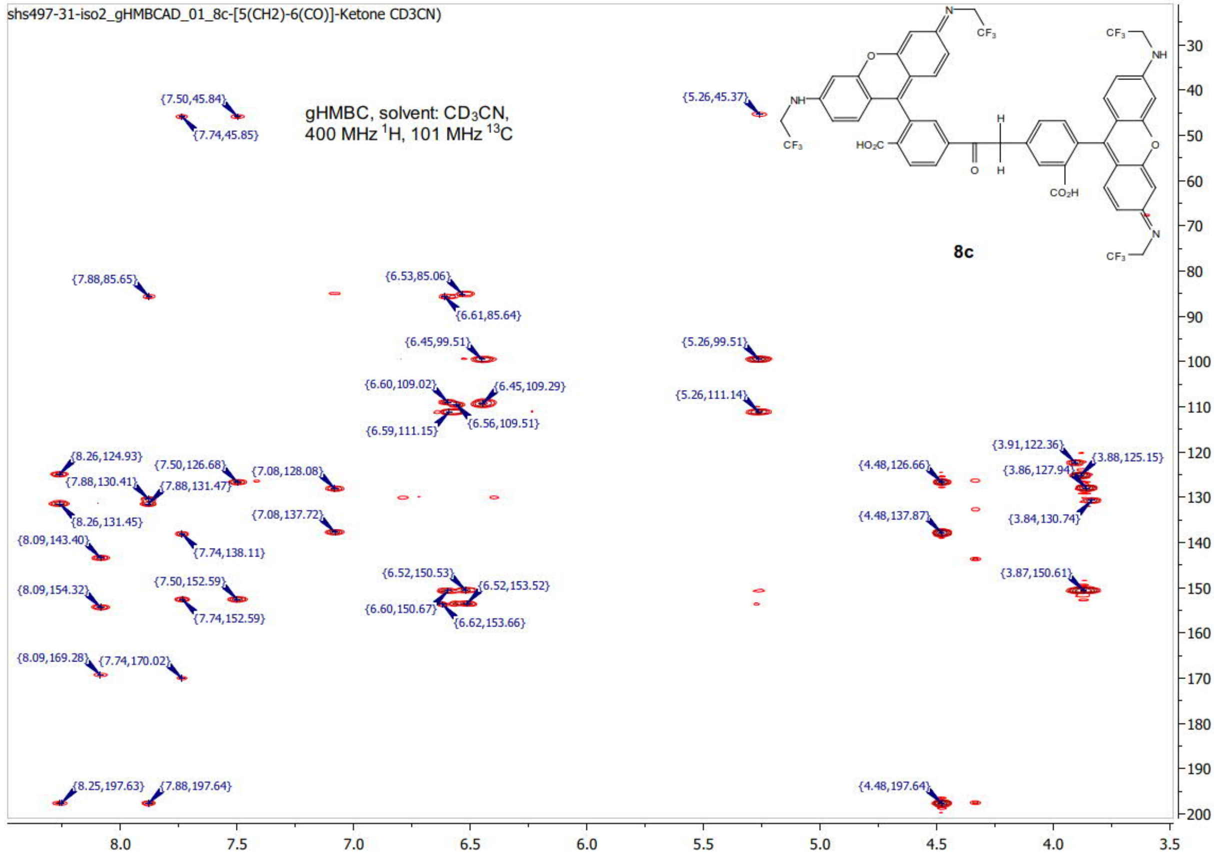
shs497-31-iso2_gHMBCAD_01_8c-[5(CH2)-6(CO)]-Ketone CD3CN)
gHMBC, solvent: CD3CN,
400 MHz 1H, 101 MHz 13C
8c

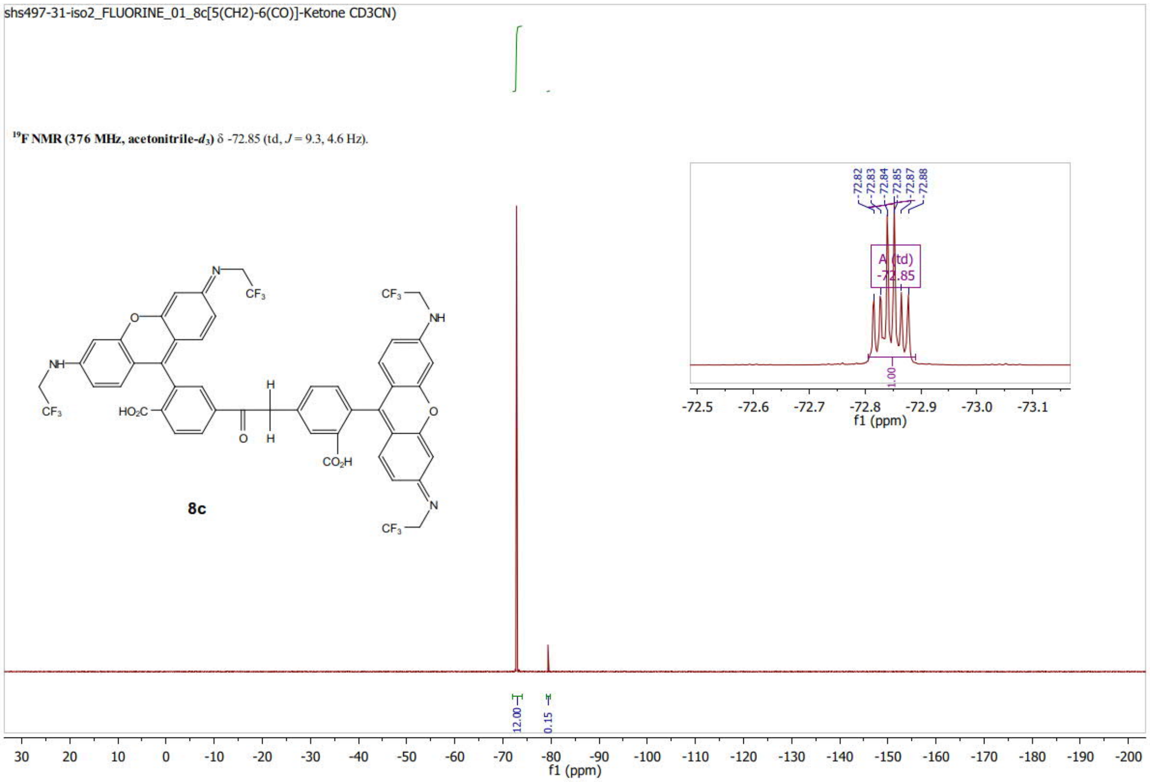

NMR spectra of compound **8d** [6(CH_2)-6(CO)]: ^{1}H (400 MHz) and $^{13}C\{^{1}H\}$ (101 MHz)

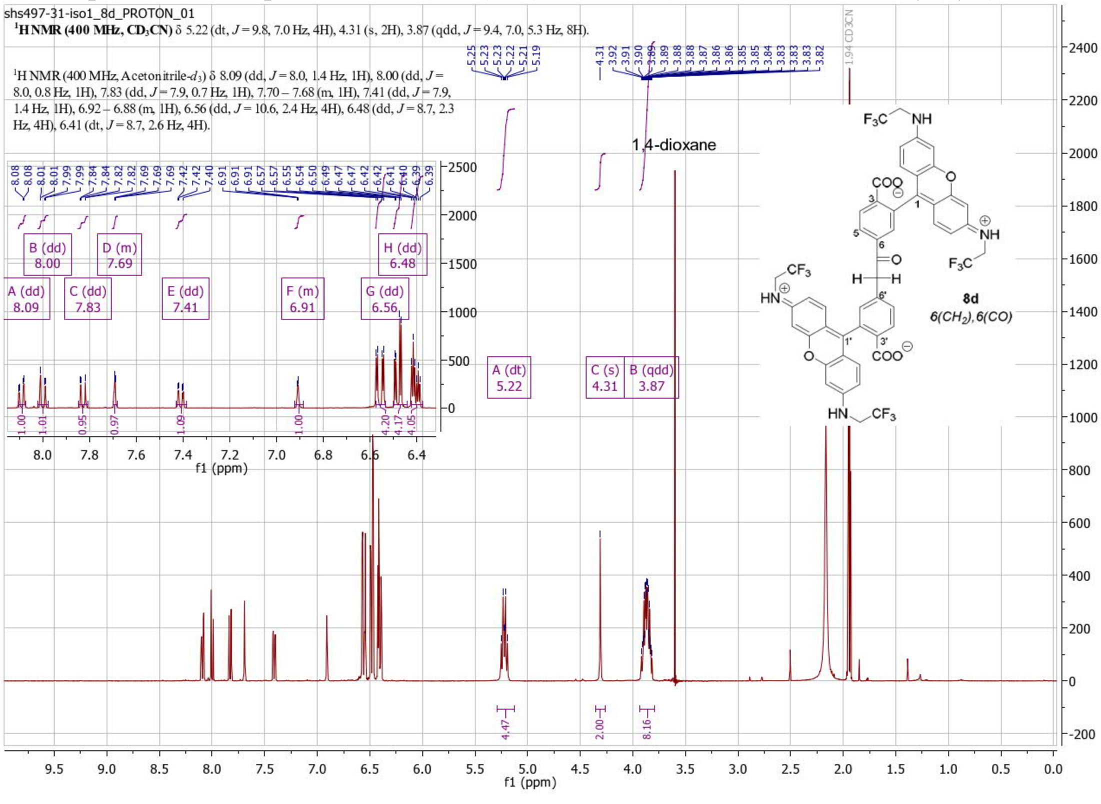

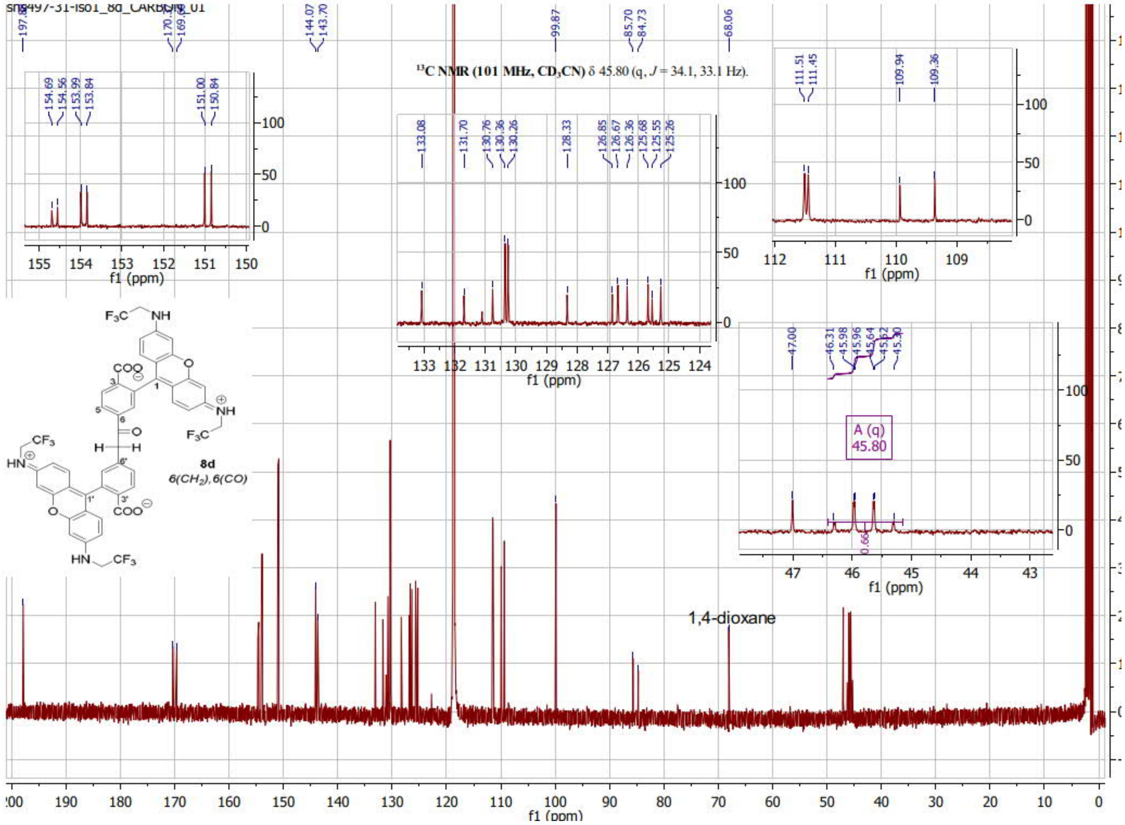

Bis-rhodamine diazoketones **1a** [5(N_2),5(CO)] (isomer 4), **1b** [6(N_2),5(CO)] (isomer 3), **1c** [5(N_2),6(CO)] (isomer 1) and **1d** [6(N_2),6(CO)] (isomer 2)

HPLC trace, isomer 1, **1c** [5(N_2),6(CO)]

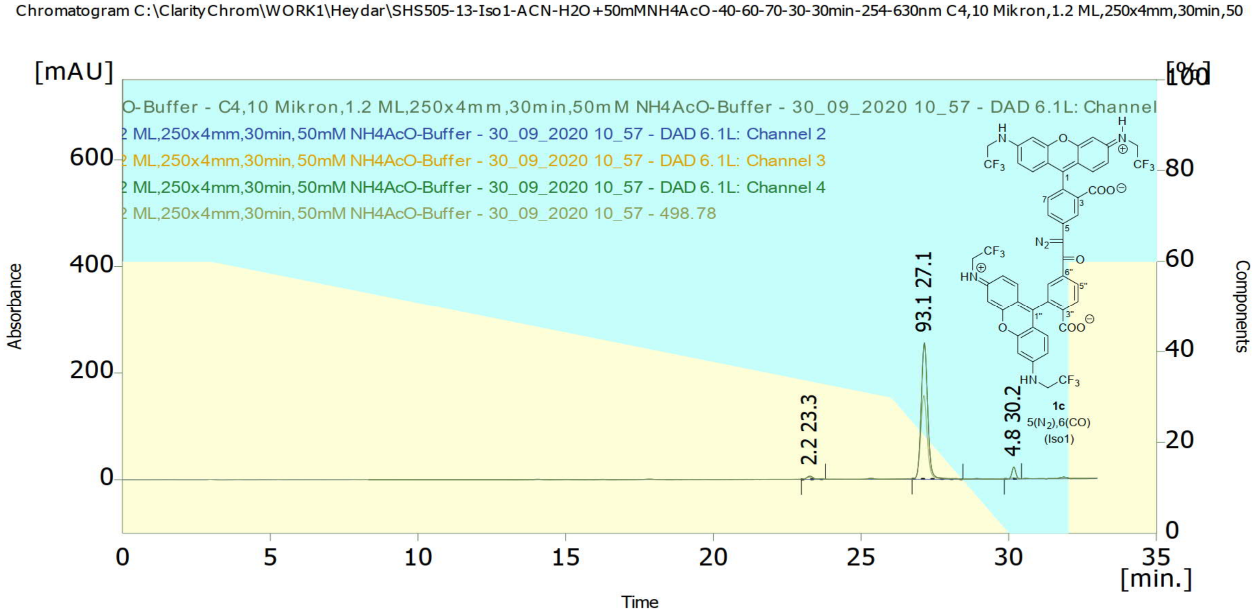

HPLC trace, isomer 2, **1d** [6(N_2),6(CO)]

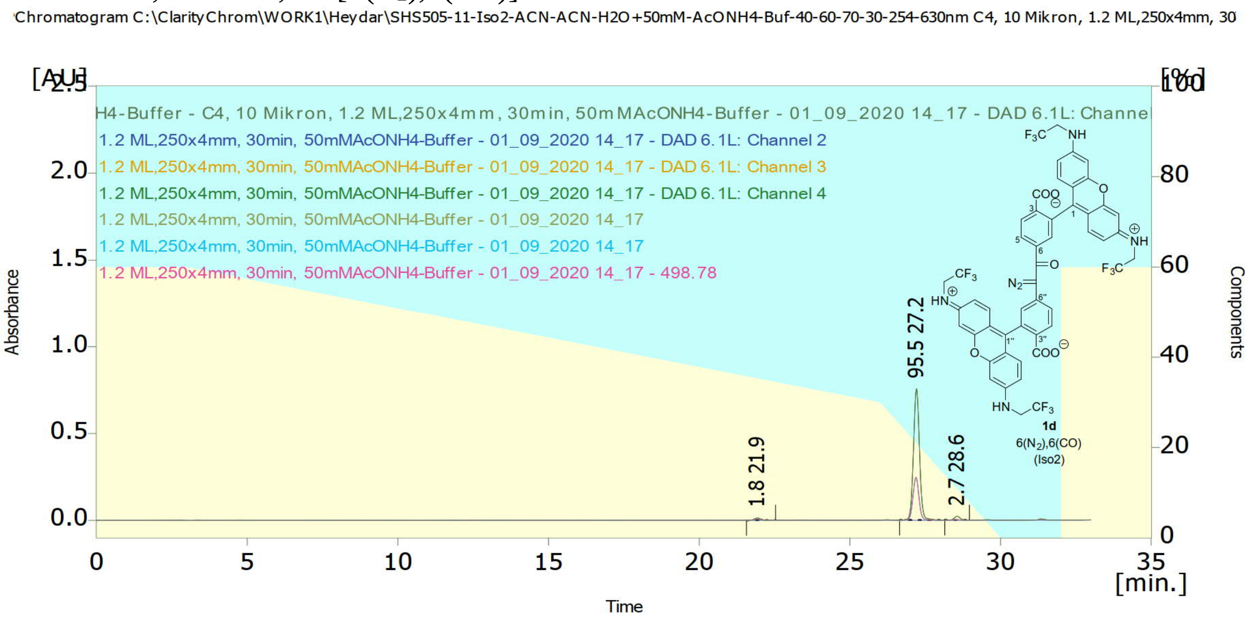

HPLC trace, isomer 3, **1b** [6(N_2),5(CO)]

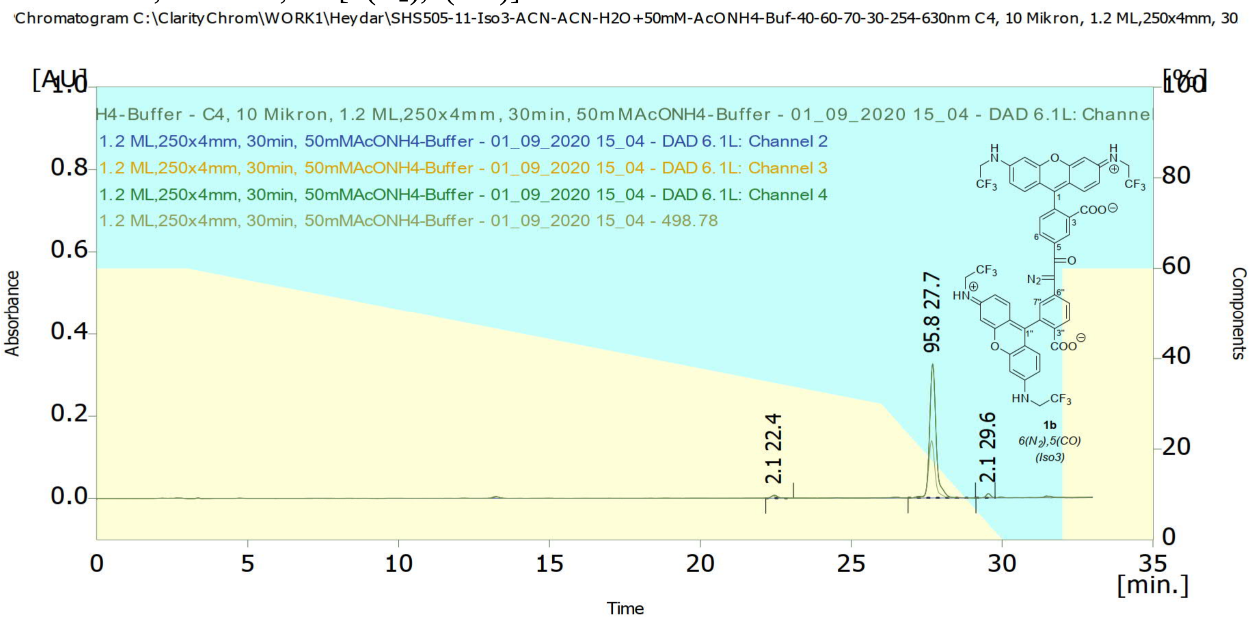

HPLC trace, isomer 4, **1a** [5(N_2),5(CO)]

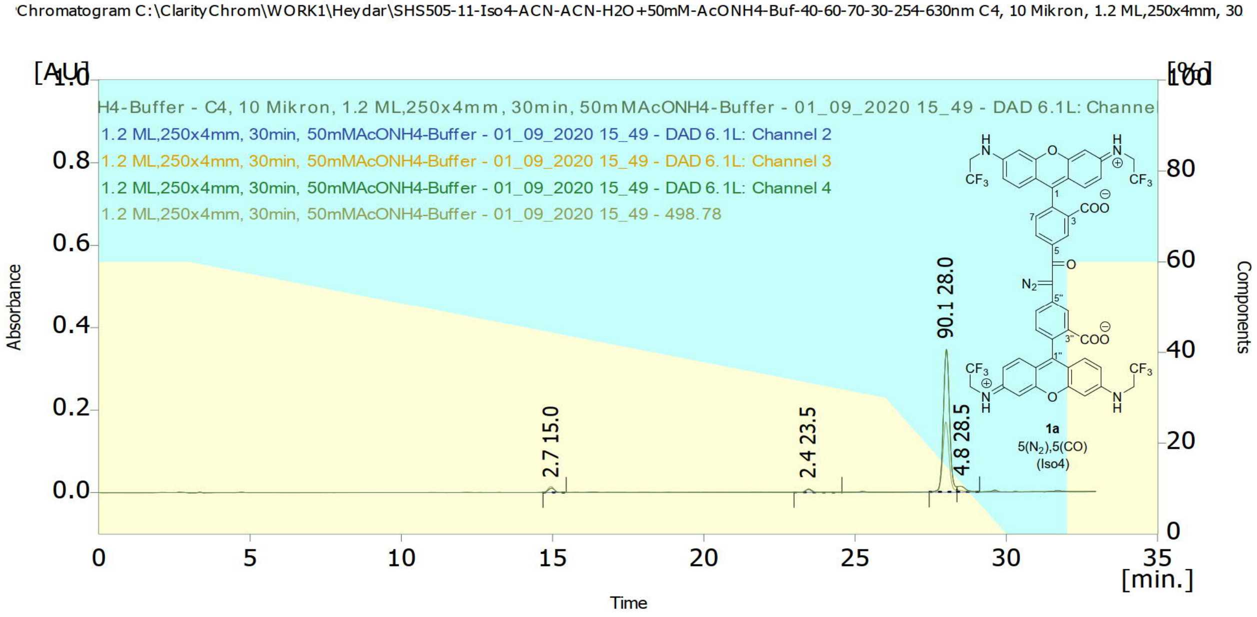

NMR spectra of compound **1a** [5(N_2), 5(CO)] (isomer 4)

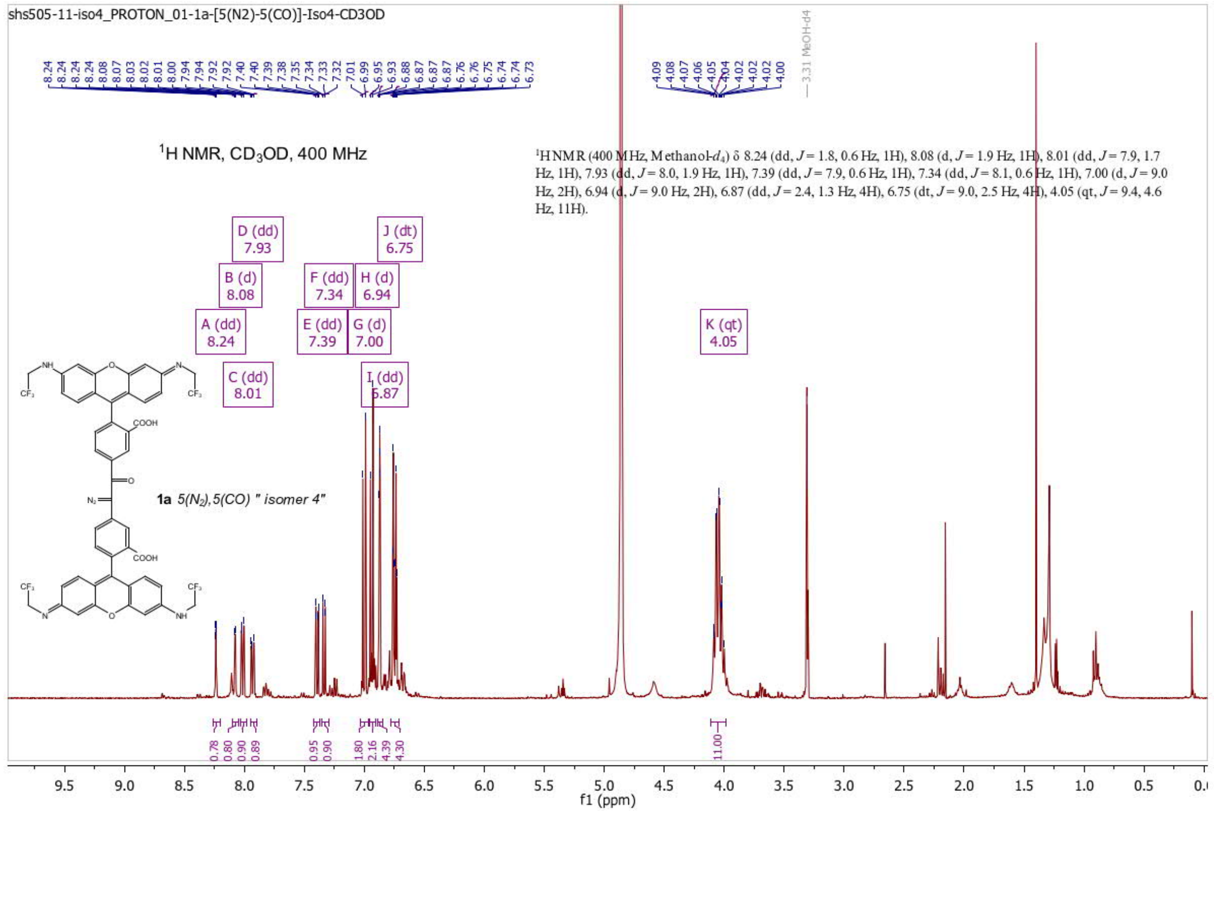

1a *5(N₂),5(CO) " isomer 4"*

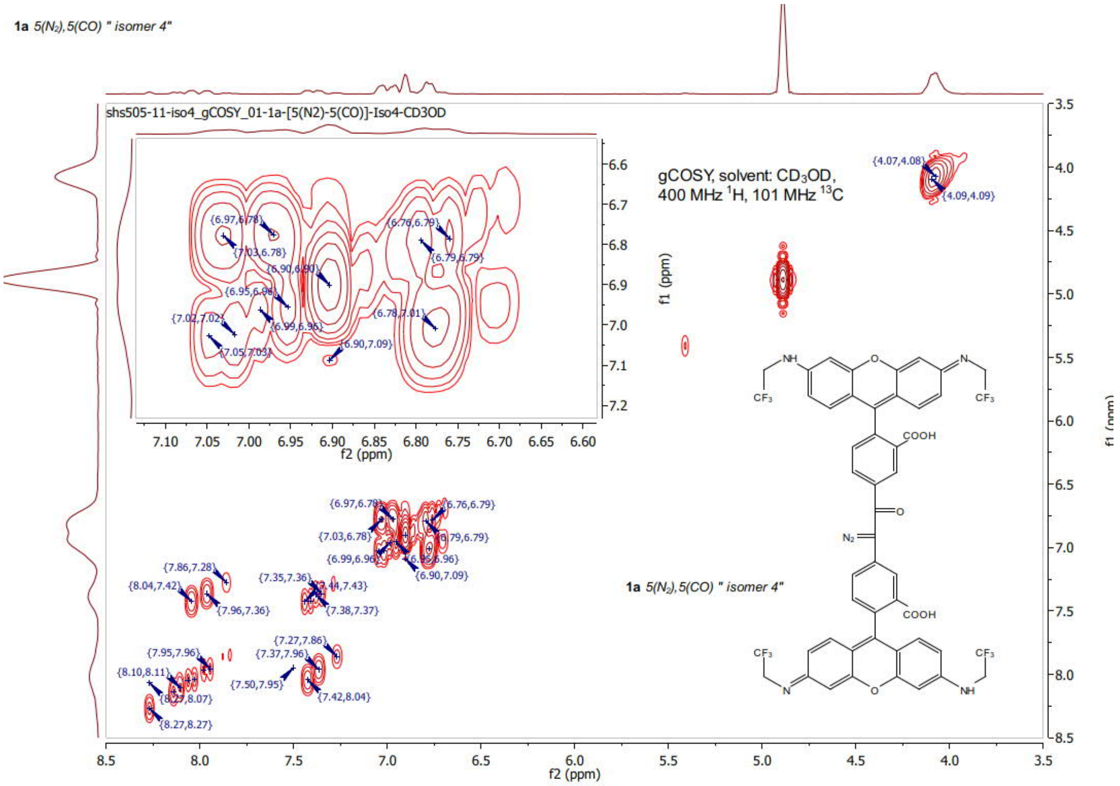

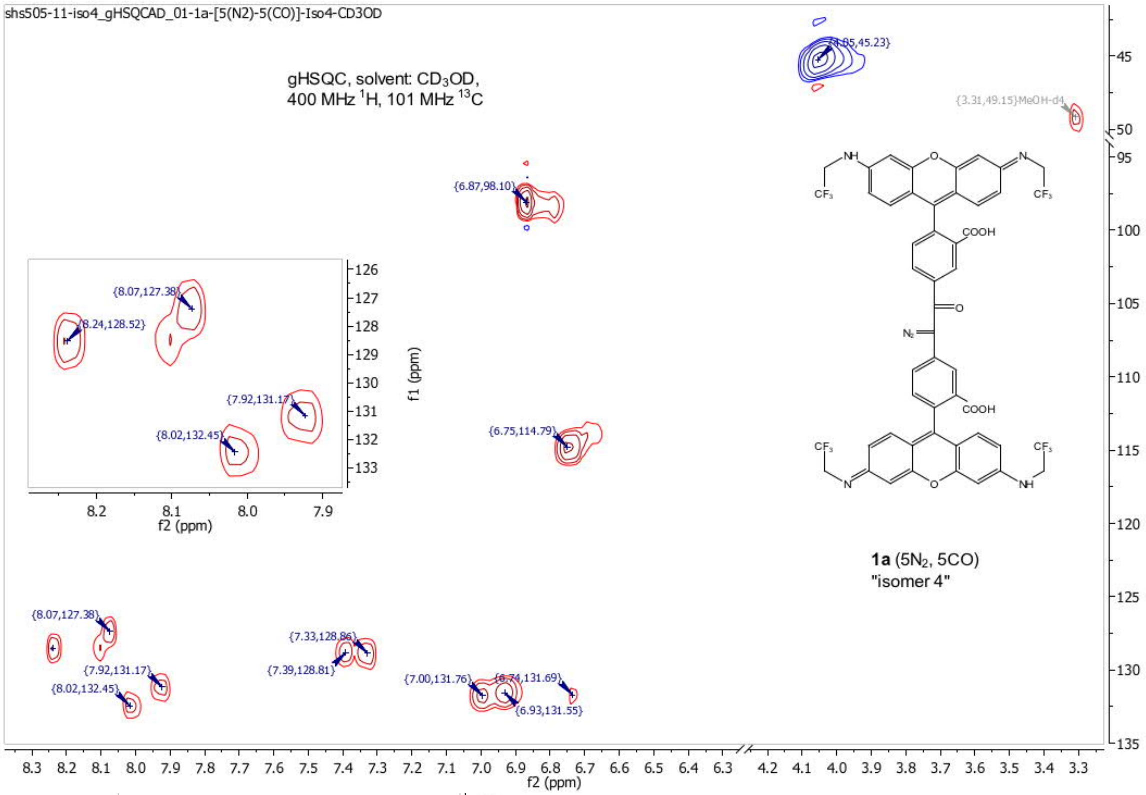
shs505-11-iso4_gHSQCAD_01-1a-[5(N2)-5(CO)]-Iso4-CD3OD
gHSQC, solvent: CD3OD,
400 MHz 1H, 101 MHz 13C
1a (5N2, 5CO)
"isomer 4"
f2 (ppm)
f1 (ppm)

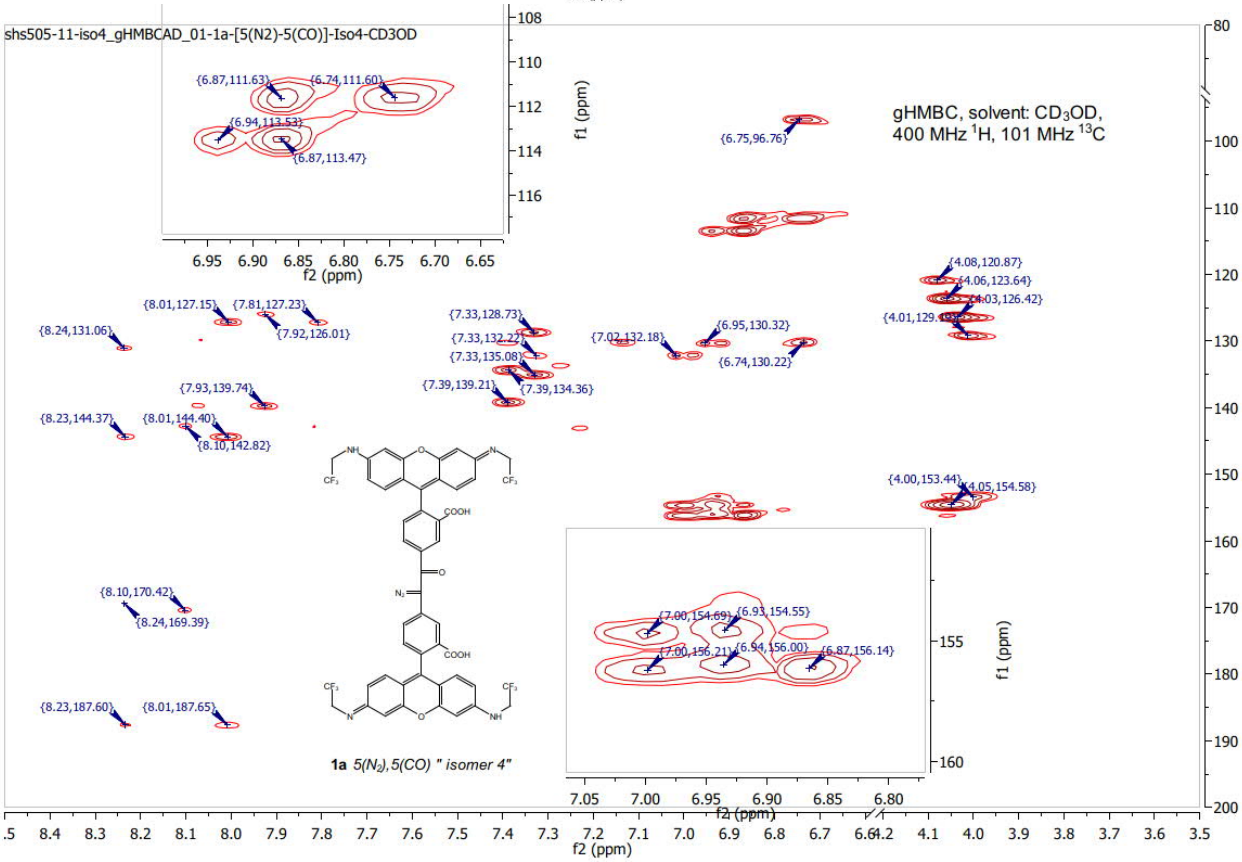
shs505-11-iso4_gHMBCAD_01-1a-[5(N2)-5(CO)]-Iso4-CD3OD
gHMBC, solvent: CD3OD,
400 MHz 1H, 101 MHz 13C
1a 5(N2),5(CO) " isomer 4"
f2 (ppm)
f1 (ppm)

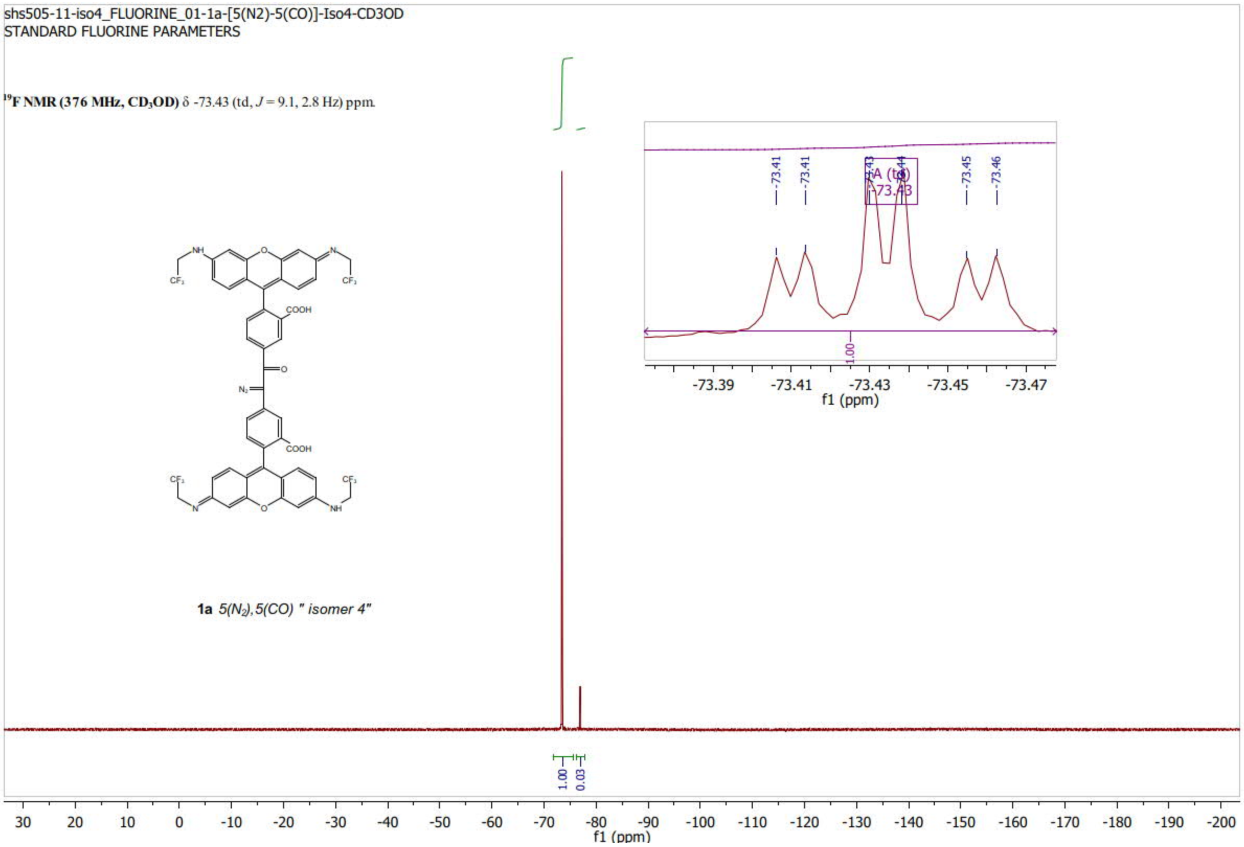

NMR spectra of compound **1b** [6(N_2),5(CO)] (isomer 3)

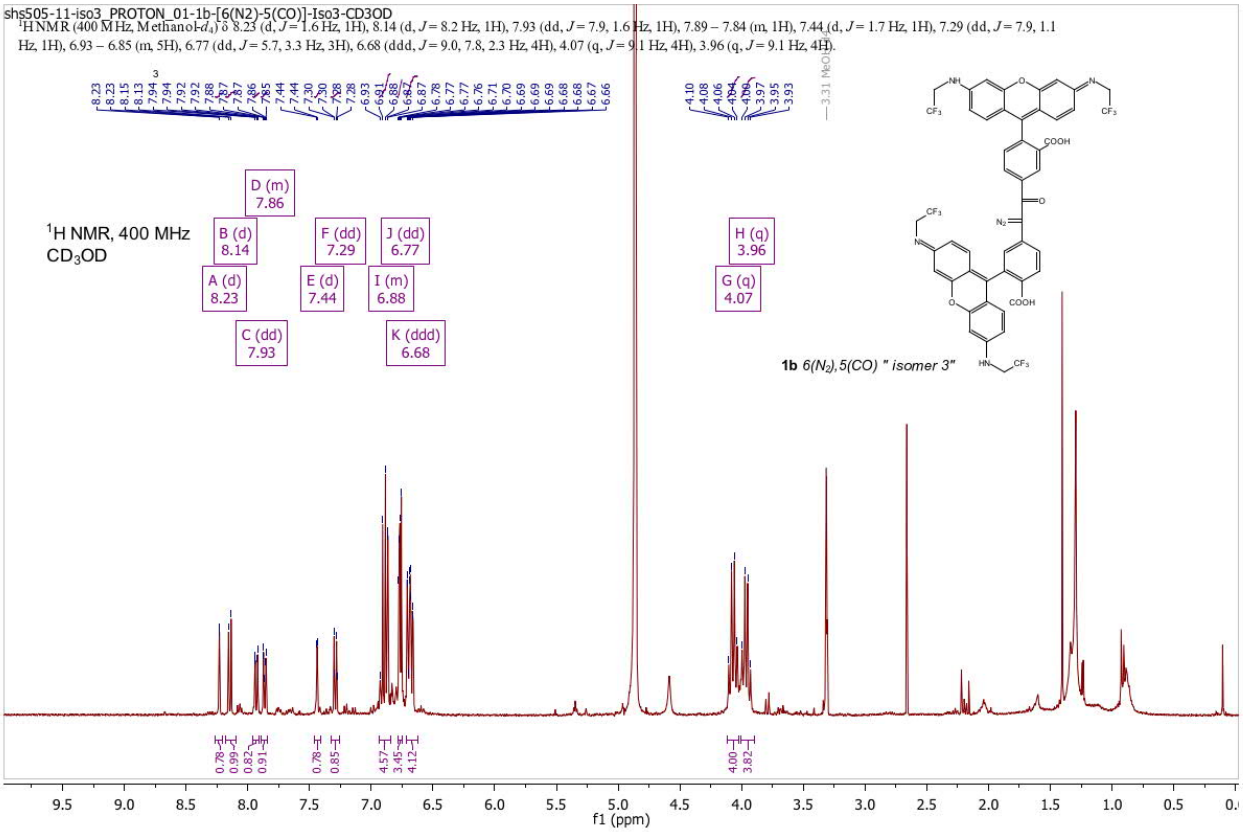

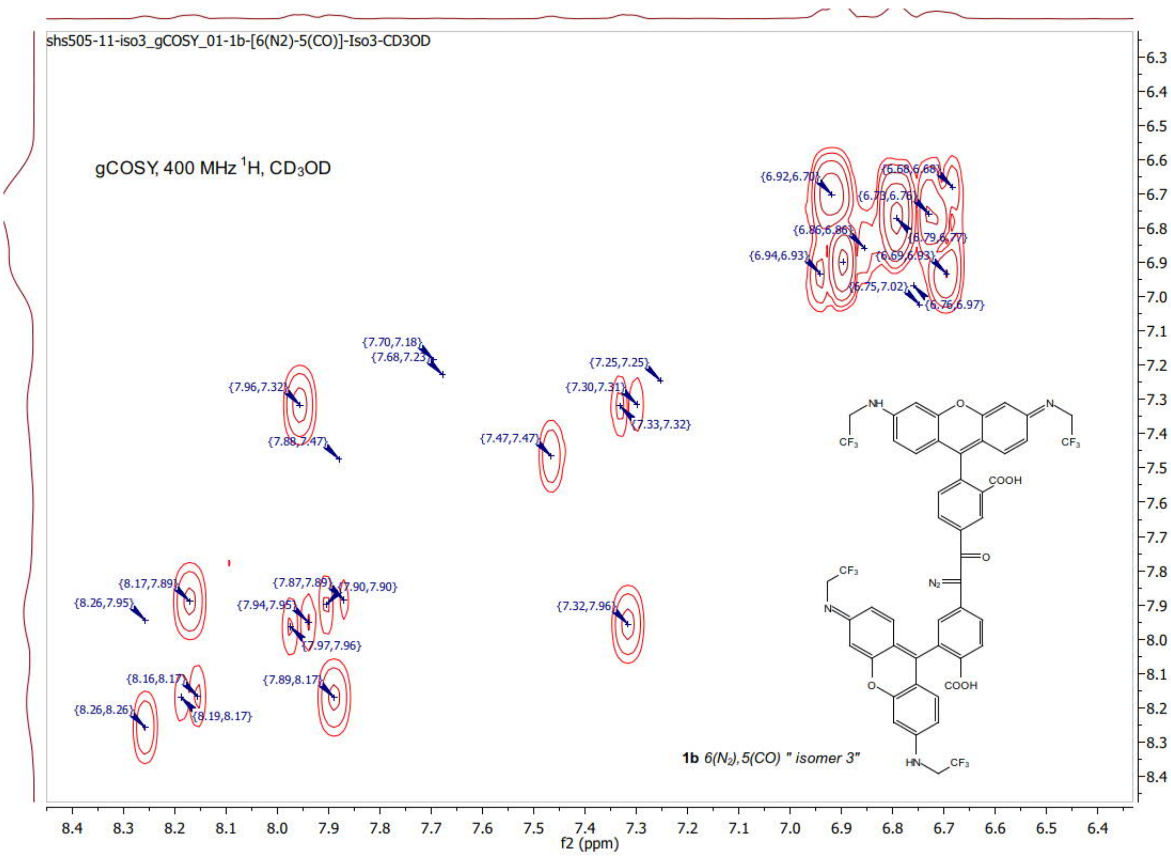

^{1}H-^{13}C NMR ((400, 101) MHz, cd$_3$od) δ (8.26 126.72), (8.17 127.60), (7.95 131.24), (7.89 126.44), (7.46 123.38), (7.31 127.24), (6.92 129.86), (6.90 96.86), (6.79 97.13), (6.79 129.91), (6.71 112.85), (4.09 43.82), (4.00 44.05) ppm.

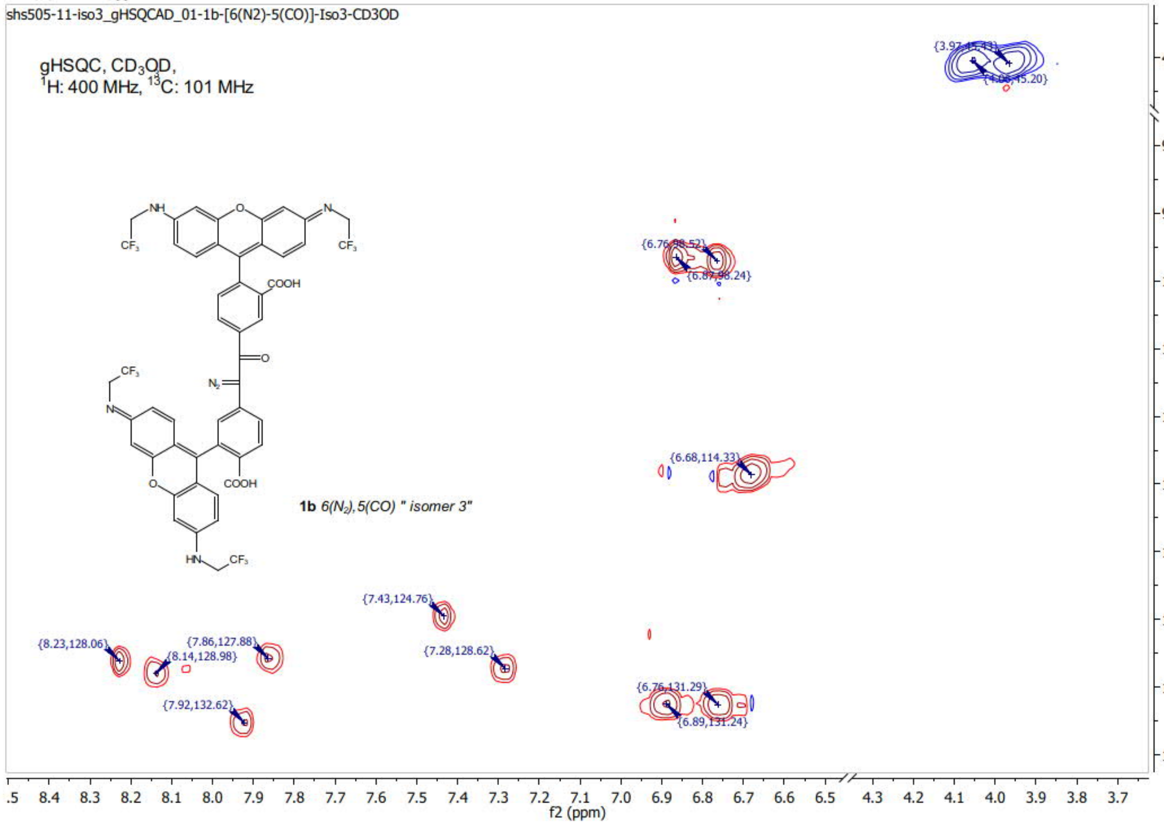

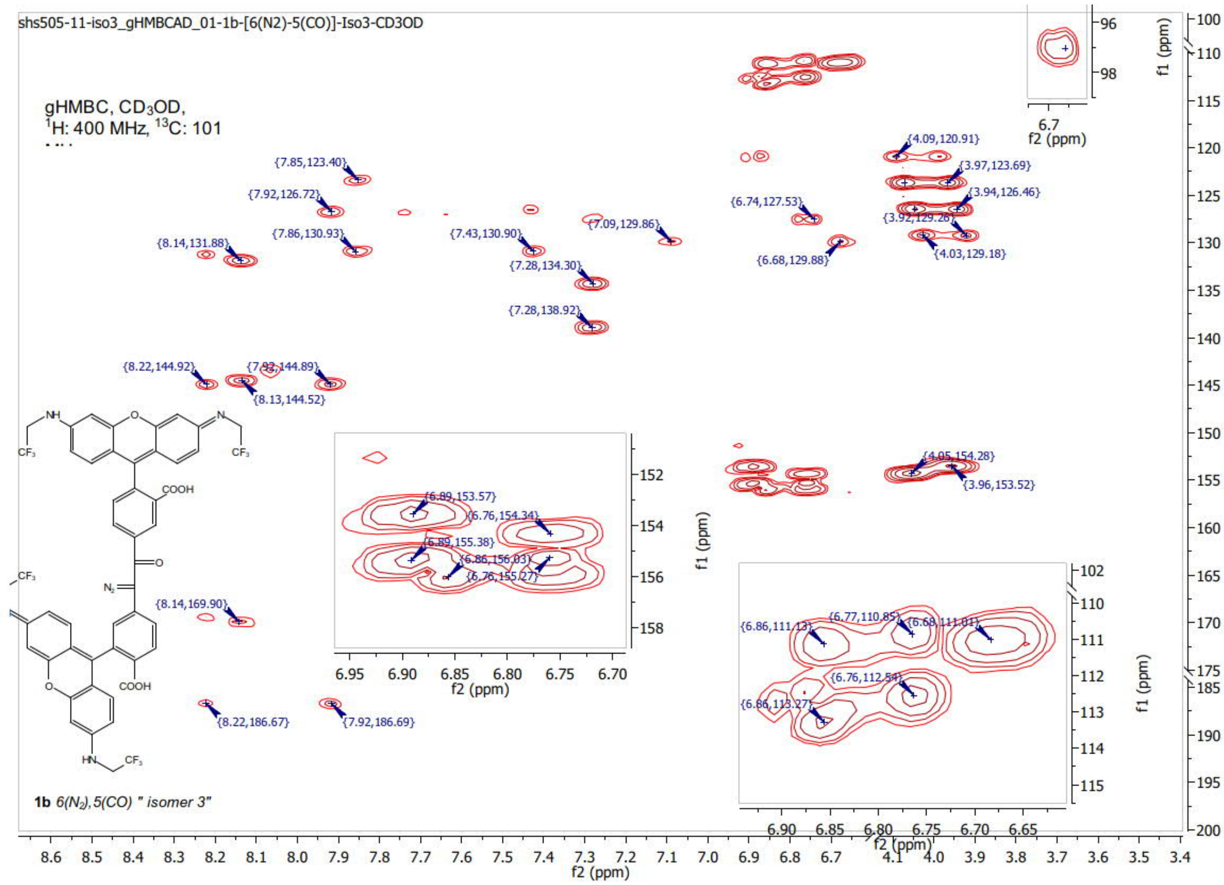
shs505-11-iso3_gHMBCAD_01-1b-[6(N2)-5(CO)]-Iso3-CD3OD
gHMBC, CD$_3$OD,
^{1}H: 400 MHz, ^{13}C: 101
1b 6(N$_2$),5(CO) " isomer 3"
f2 (ppm)
f1 (ppm)

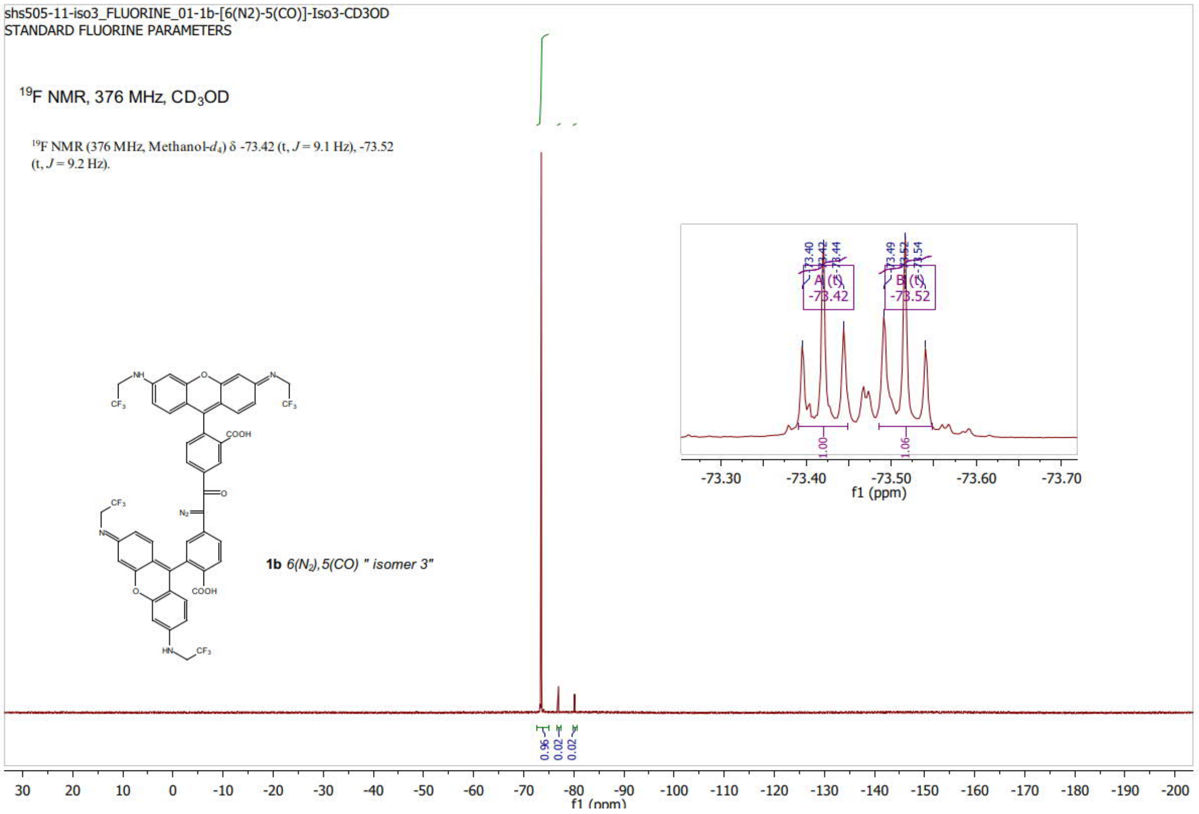
shs505-11-iso3_FLUORINE_01-1b-[6(N2)-5(CO)]-Iso3-CD3OD
STANDARD FLUORINE PARAMETERS
^{19}F NMR, 376 MHz, CD$_3$OD
^{19}F NMR (376 MHz, Methanol-d_4) δ -73.42 (t, J = 9.1 Hz), -73.52 (t, J = 9.2 Hz).
1b 6(N$_2$),5(CO) " isomer 3"
A (t) -73.42
B (t) -73.52
f1 (ppm)

Compound **1c** [5(N_2),6(CO)] (isomer 1)

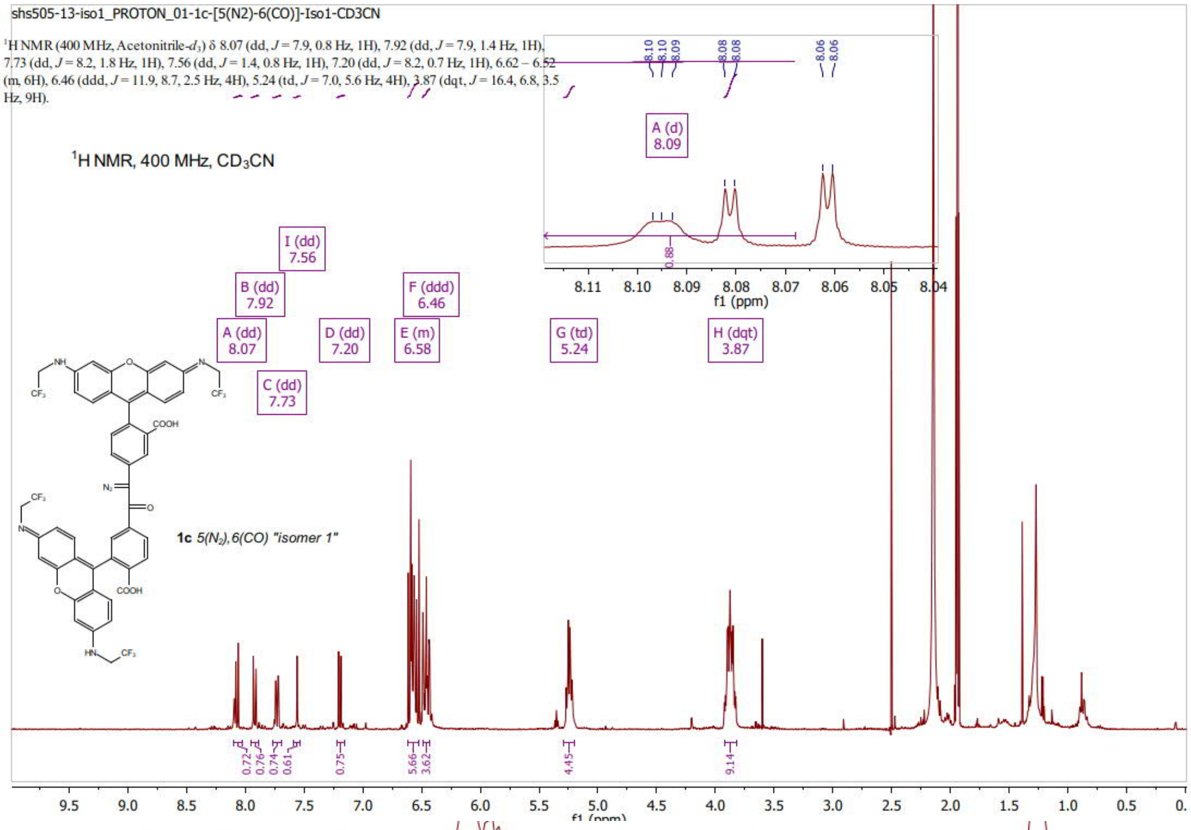

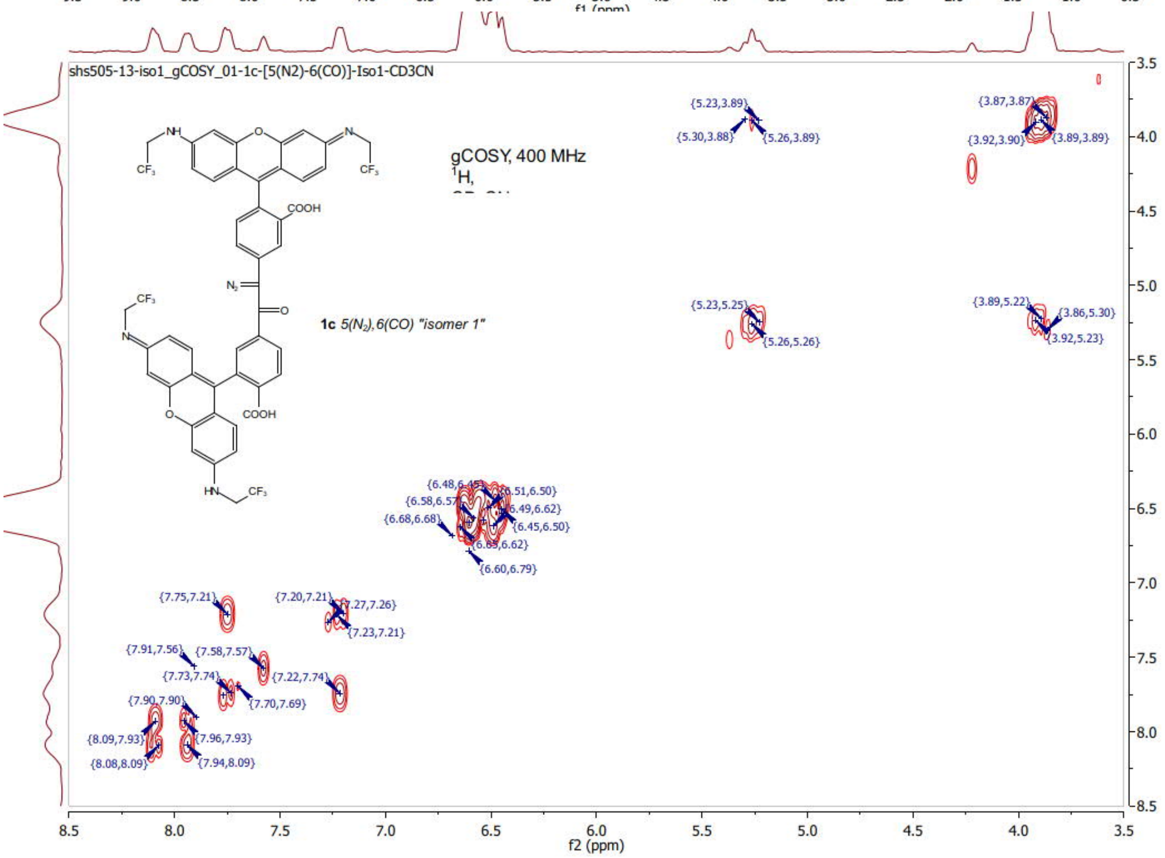

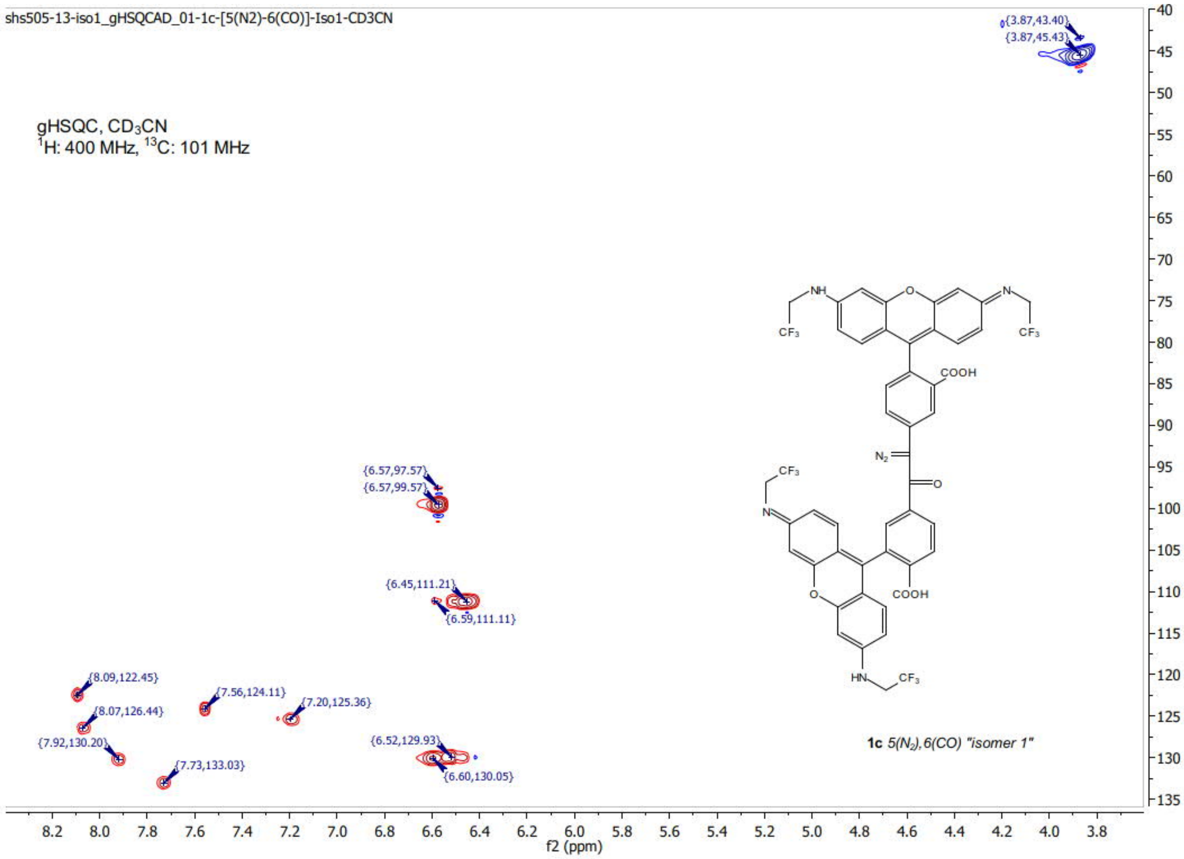

shs505-13-iso1_gHSQCAD_01-1c-[5(N2)-6(CO)]-Iso1-CD3CN
gHSQC, CD3CN
1H: 400 MHz, 13C: 101 MHz
1c 5(N2),6(CO) "isomer 1"
f2 (ppm)

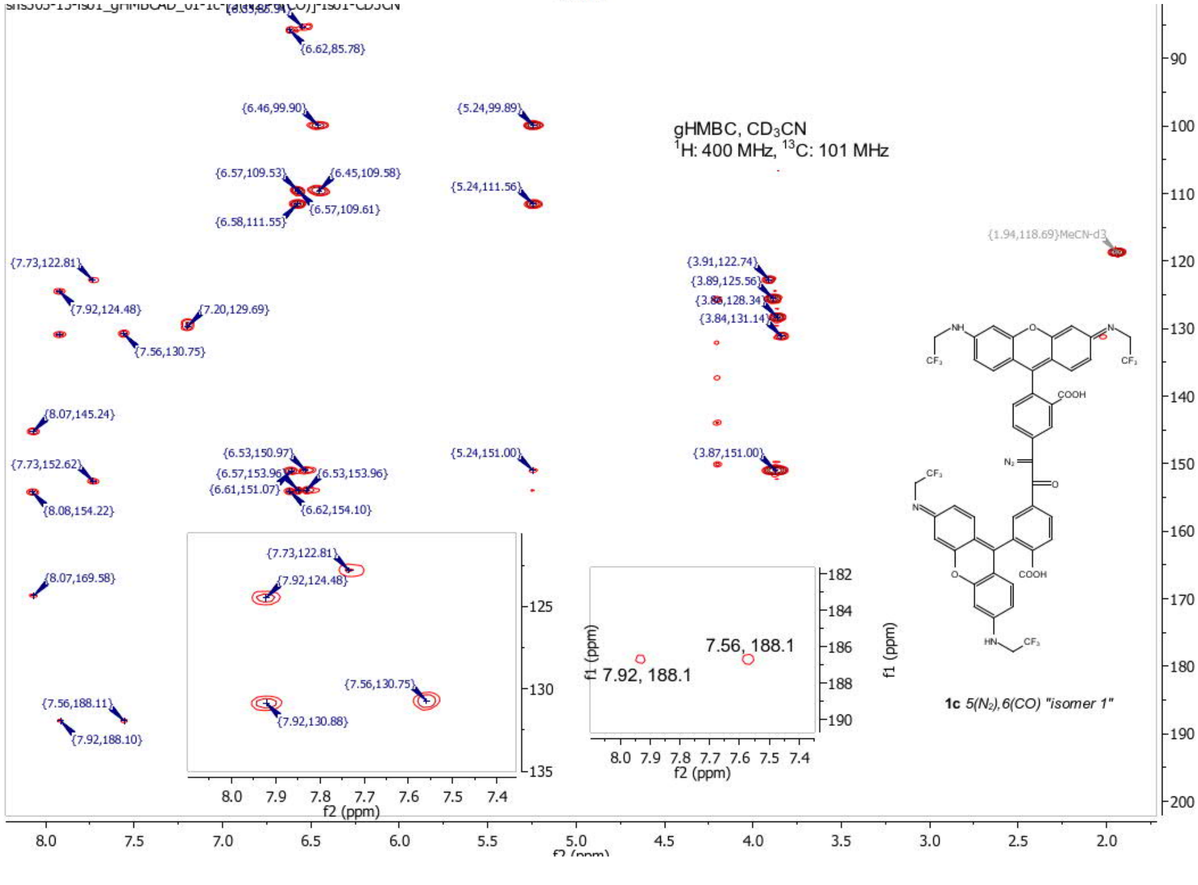

gHMBC, CD3CN
1H: 400 MHz, 13C: 101 MHz
1c 5(N2),6(CO) "isomer 1"
7.56, 188.1
7.92, 188.1
f1 (ppm)
f2 (ppm)

^{19}F NMR (376 MHz, Acetonitrile-d_3) δ -72.86 (td, J = 9.4, 2.1 Hz, 12F) ppm.

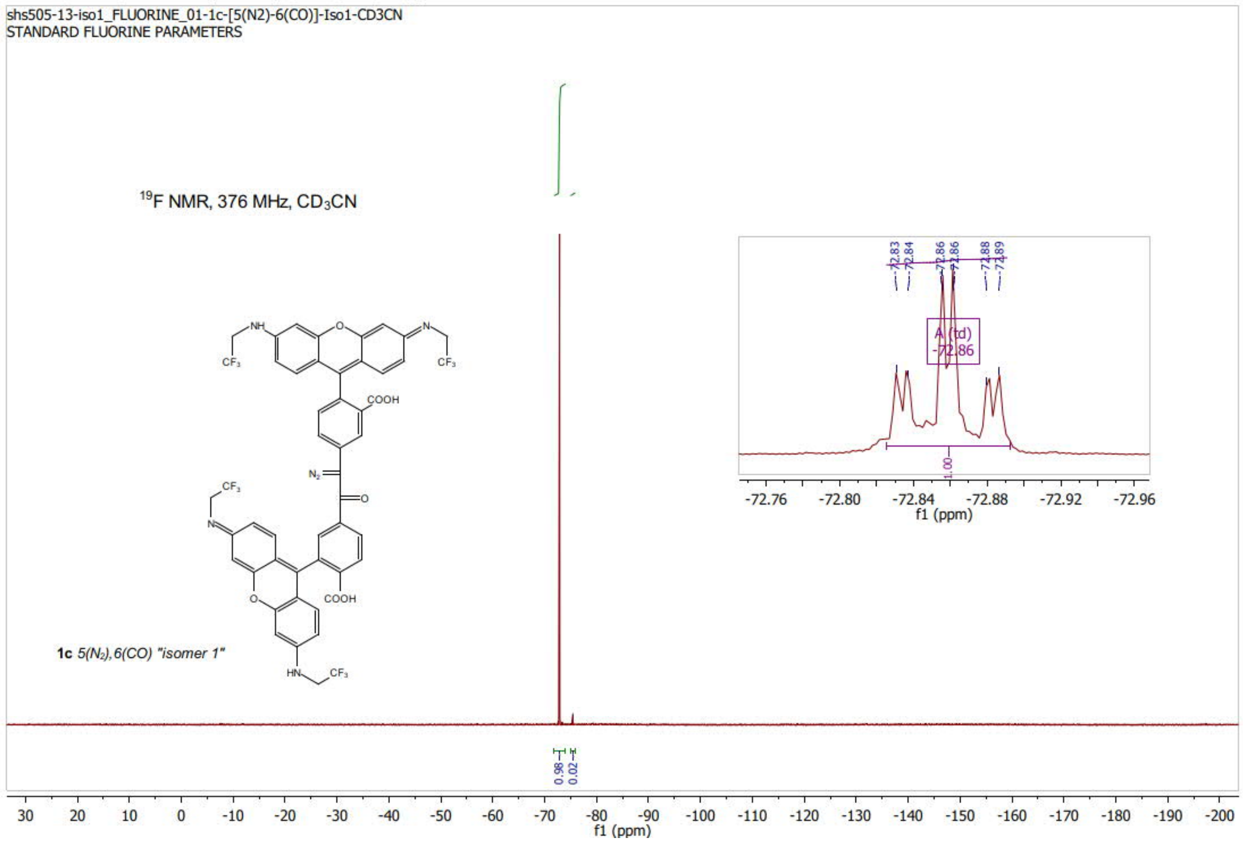

NMR spectra of compound **1d** [6(N_2),6(CO)] (isomer 2)

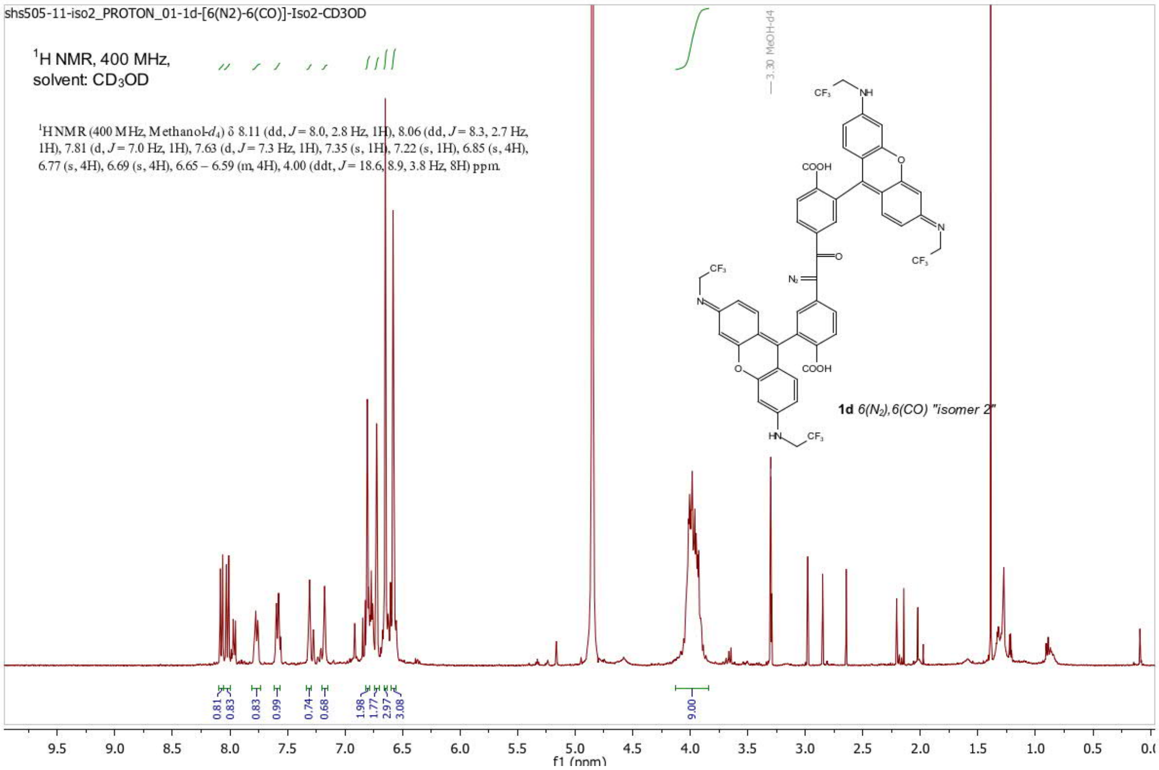

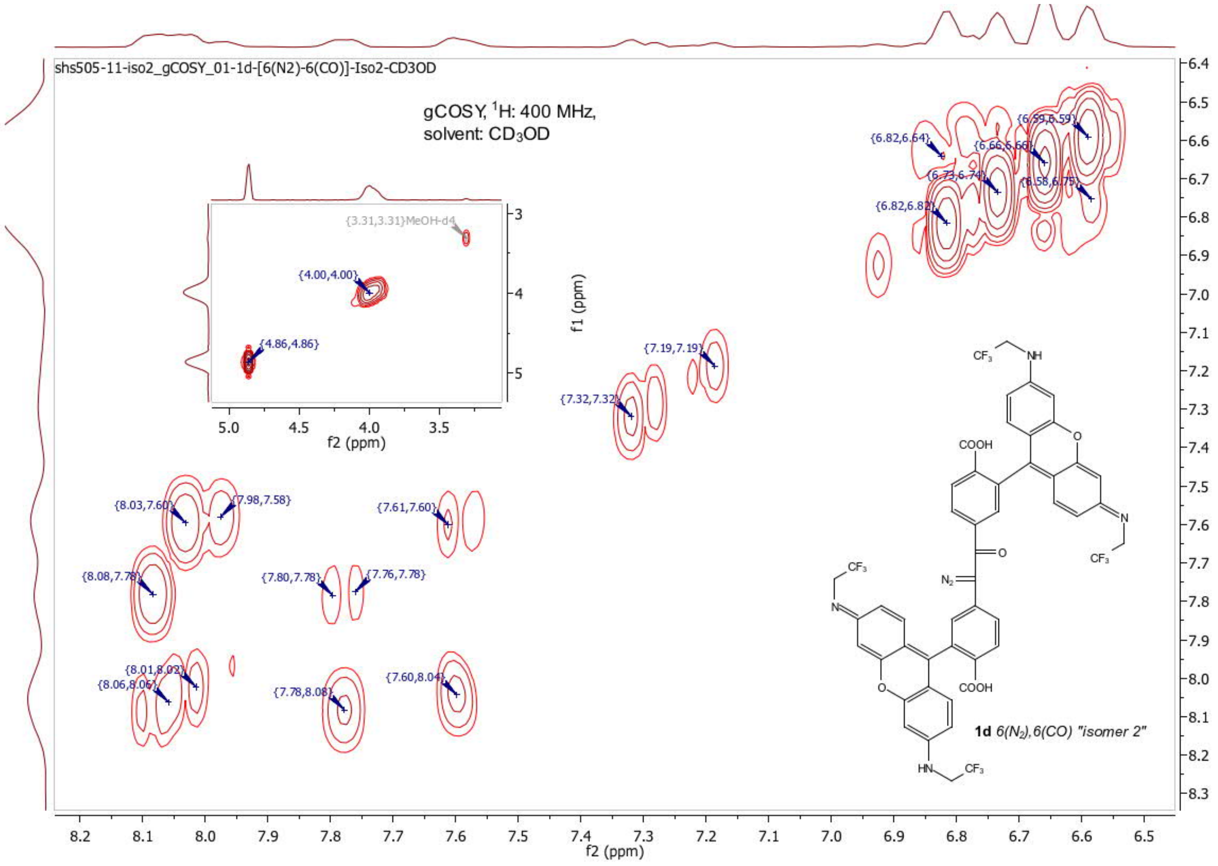
shs505-11-iso2_gCOSY_01-1d-[6(N2)-6(CO)]-Iso2-CD3OD
gCOSY, 1H: 400 MHz,
solvent: CD3OD
1d 6(N2),6(CO) "isomer 2"
f1 (ppm)
f2 (ppm)

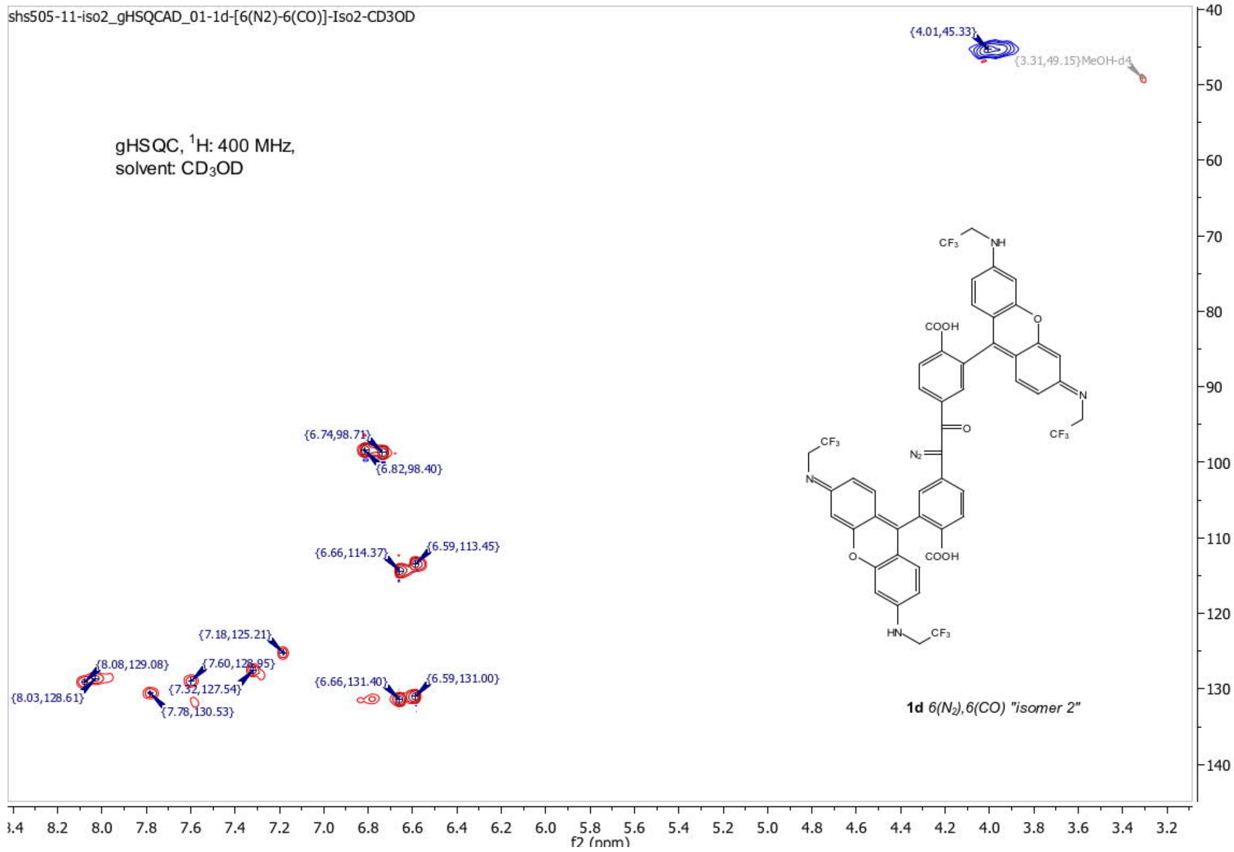
shs505-11-iso2_gHSQCAD_01-1d-[6(N2)-6(CO)]-Iso2-CD3OD
gHSQC, 1H: 400 MHz,
solvent: CD3OD
1d 6(N2),6(CO) "isomer 2"
f2 (ppm)

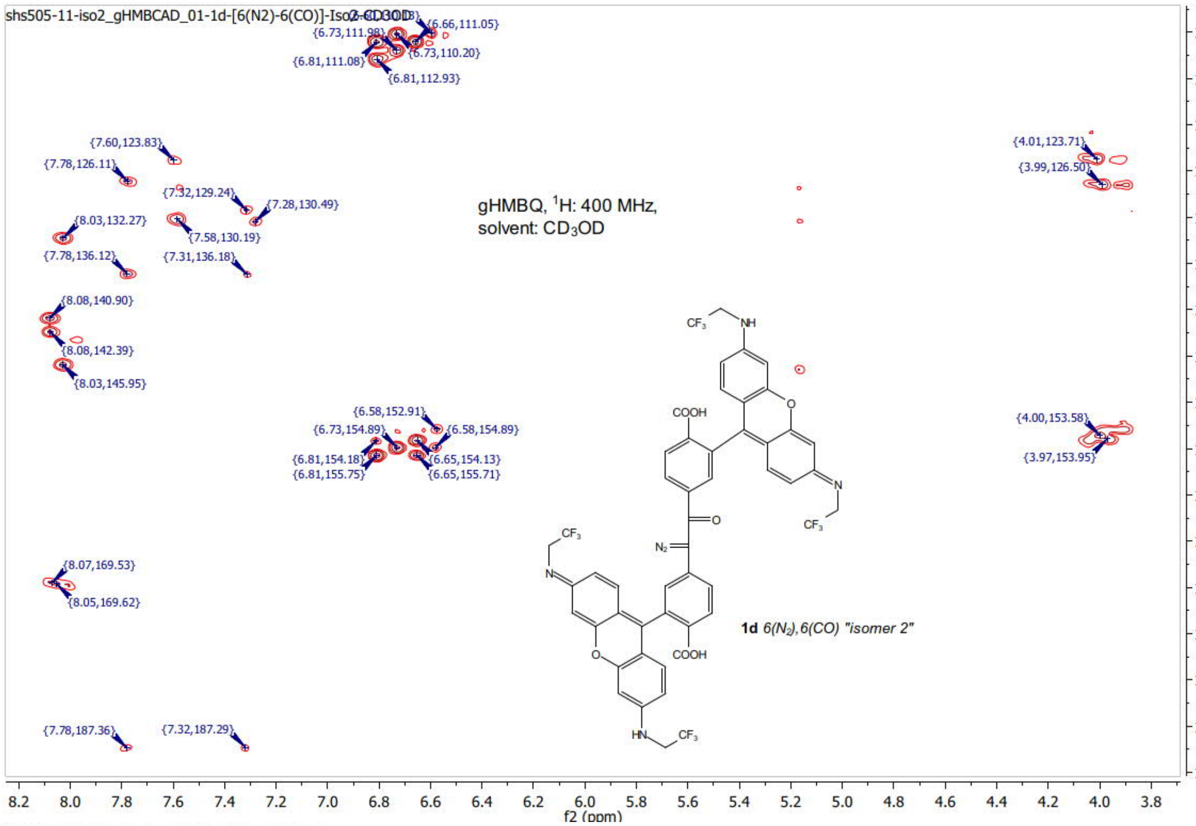

^{19}F NMR (376 MHz, Methanol-d_4) δ -71.80 – -74.71 (m) ppm.

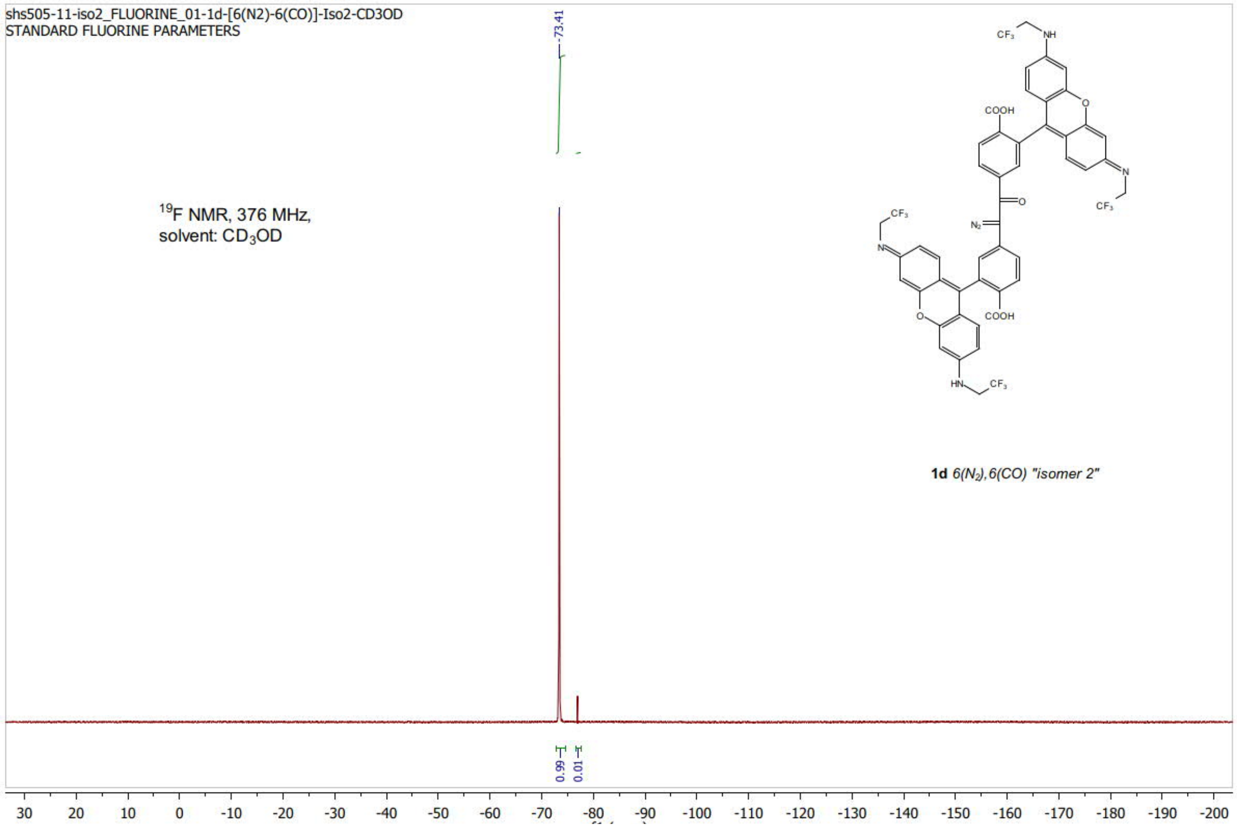

Supplement: Supplementary file 1 — jo1c01721_si_001.pdf [file jo1c01721_si_001.pdf]
